# Enzymatic synthesis of novel pyrrole esters and their thermal stability

**DOI:** 10.1186/s13065-023-01039-5

**Published:** 2023-09-23

**Authors:** Jingyi Hu, Meng Zhou, Yujie Zhang, Xi Zhang, Xiaoming Ji, Mingqin Zhao, Miao Lai

**Affiliations:** 1 Flavors and Fragrance Engineering & Technology Research Center of Henan Province, College of Tobacco Science, Henan Agricultural University, Zhengzhou, 450002 People’s Republic of China; 2 Technology Center, China Tobacco Hebei Industrial Co., Ltd., Shijiazhuang, 050051 People’s Republic of China; 3 Technology Center, China Tobacco Shanxi Industrial Co., Ltd., Xian, 710065 People’s Republic of China

**Keywords:** Pyrrolyl esters, Novozym 435, Transesterification, GC–MS–O, Py–GC/MS, TG

## Abstract

In the present work a simple enzymatic approach (Novozym 435) for transesterification to synthesize pyrrole esters was reported. To generate the best reaction conditions, which resulted in the optimum yield of 92%, the effects of lipase type, solvent, lipase load, molecular sieves, substrate molar ratio of esters to alcohol, reaction temperature, reaction duration, and speed of agitation were evaluated. The range of alcohols was assessed under optimal circumstances. The spectrum observations conclusively demonstrated that the compounds could be generated with high yield under the circumstances utilized for synthesis. The odor characteristics of the pyrrolyl esters obtained were examined by gas chromatography–mass spectrometry-olfactometry (GC–MS–O). Among them, compounds of benzhydryl 1*H*-pyrrole-2-carboxylate (**3j**), butyl 1*H*-pyrrole-2-carboxylate (**3k**) and pentyl 1*H*-pyrrole-2-carboxylate (**3l**) present sweet and acid aroma. In addition, the thermal degradation process was further studied using the Py–GC/MS (pyrolysis–gas chromatography/mass spectrometry), TG (thermogravimetry), and DSC (differential scanning calorimeter) techniques. The outcomes of the Py–GC/MS, TG, and DSC techniques show that they have excellent thermal stability.

**Supplementary Information:**

The online version contains supplementary material available at 10.1186/s13065-023-01039-5.

## Introduction

Esters are often utilized in the food, fragrance, cosmetic, coating, and pharmaceutical sectors as flavoring and aroma ingredients [1–3]. Heteroaryl esters, which are essential building blocks for organic synthesis and could create interesting building blocks for the creation of diverse functionalized products, are described in more detail below [4–9]. Particularly, pyrrole esters, which could be characterized by special aromatic organoleptic qualities, have found use in bioactive compounds. As a result, making pyrrole esters has garnered more interest [10]. Thus, there has been a growing interest in preparing pyrrole esters. Chemical synthesis is an alternate technique for producing pyrrole esters, however it has disadvantages including high temperature, poor yield, and catalyst residue. However, biocatalytic synthesis of flavor esters is becoming more and more popular in the flavor and fragrance industries, due to the advantages of selectivity and specificity, reusability, mild conditions and much purer products [11–15]. Esterification, interesterification, thioesterification, and transesterification processes are produced by enzymes such lipases and esterases and are catalyzed by the ping-pong bibi mechanism, ternary complex ordered bibi mechanism, or ternary complex random bibi mechanism [16–19]. A variety of functional groups, halides, and linear as well as cyclic alkyl alcohols are allowed under this procedure. For instance, several citronellyl esters [20, 21], propyl benzoate [22], adipate ester [15], ethyl hexanoate [23], monoterpenic esters [24], aliphatic esters [25], benzyl benzoate [26], pentyl valerate [27] were efficiently synthesized with the assistant of enzymes catalysis. Immobilized lipases are harmless, biodegradable catalysts that speed up chemical processes at low temperatures and pressures. Herein we report a new approach to obtain a series of pyrrole esters using methyl pyrrole-carboxylate and simple alcohols via Novozym 435-catalyzed esterification with *n*-hexane as the solvent. A variety of functional groups, halides, and linear as well as cyclic alkyl alcohols are allowed under this approach. In furthermore, gas chromatography–mass spectrometry-olfactometry (GC–MS–O) might be used to evaluate the olfactory properties and potential of the compounds as flavors or perfumes. In addition, the thermal behaviors of the pyrrole esters under high temperatures were investigated using the pyrolysis–gas chromatography/mass spectrometry (Py–GC/MS), thermogravimetry (TG), and differential scanning calorimeter (DSC) techniques [28–30].

In this paper, we provide the enzymatic production, odor characteristic, and thermal behaviors of new pyrrole ester derivatives. The effects of several variables such as kind of lipase, different solvents, molecular sieves load, biocatalyst load, substrate concentration, reaction temperature, time, speed of agitation and rate of reaction were systematically investigated to generate the best reaction conditions. The compatibility of various alcohols with this protocol is evaluated. GC–MS–O was also used to analyze the pyrrole esters' olfactory properties. By using Py–GC/MS and TG, the heat stability of the flavoring compounds was evaluated. These findings provide direction for the creation of efficient pyrrole flavoring agents.

## Results and discussion

### Effect of the lipase on lipase-catalyzed transesterification

**Fig. 1:**
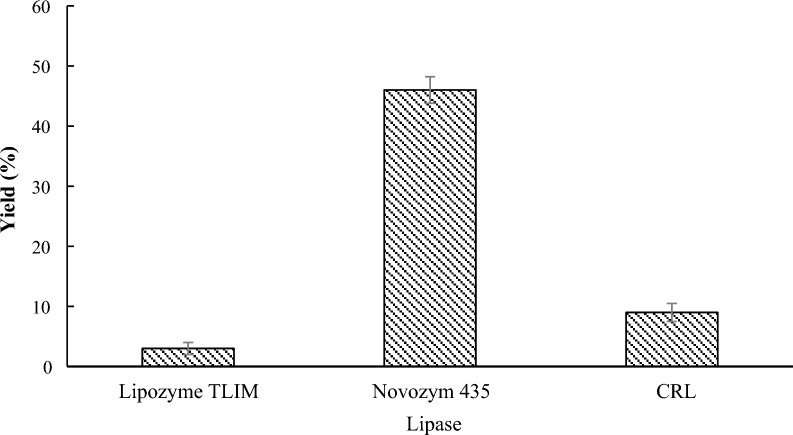
Effect of the lipase on lipase-catalyzed transesterification. Reaction conditions: methyl 1*H*-pyrrole-2-carboxylate **1a** (1.0 mmol), benzyl alcohol **2a** (0.2 mmol), 60 mg lipase, 10 mL toluene, 1.0 g molecular sieves, 40 °C, 150 rpm, 24 h

To identify the optimal conditions, methyl 1*H*-pyrrole-2-carboxylate (**1a**) and benzyl alcohol (**2a**) were designed as model substrates for the initial investigation. For the first lipase-catalyzed transesterification of **1a** with **2a**, three available commercially encapsulated lipases, namely Lipozyme TLIM, Novozym 435, and CRL, were used. Other input investigations were 5:1 (**1a**/**2a**) reactant molar ratio, lipase load of 6 mg/mL, stirrer speed of 150 r/min, molecular sieve load of 1 g, 40 °C reaction temperature, and a 24 h reaction time. According to Fig. 1, Lipozyme TLIM provided the lowest contents of benzyl 1*H*-pyrrole-2-carboxylate (**3a**), accounting for less than 3%. Compound **3a** has a lower amount of 9% according to CRL. Under the catalysis of Novozym 435, compound **3a** was generated in the maximum concentration which showed much higher activity (46% yield) in toluene than Lipozyme TLIM and CRL. It suggested that Novozym 435 was efficient for catalyzing the transesterification of methyl 1H-pyrrole-2-carboxylate with alcohol.

### Effect of the solvent on lipase-catalyzed transesterification

The activity of three lipases is influenced by the various catalytic characteristics [28]. Candida antarctica lipase B (CALB) functioned as the source of Novozym 435. It was formerly believed that Novozym 435 was an all-purpose lipase [31]. The efficiency and stability of enzymes are substantially impacted by the reaction medium [32]. In various solvent media, lipases frequently displayed varying positional selectivity and catalytic activity, which were frequently connected to the solvent characteristic [33]. Here, the lipase Novozym 435 was used to study the effects of solvents, eight kinds of solvent, including toluene, 1,4-Dioxane, CH_3_CN, DMF, *n*-Hexane, ethanol, isooctane and DMSO, were used as the reaction medium to carry out this reaction (Table 1). Table 1 demonstrates that the *n*-Hexane significantly outperformed the competition, yielding the required product **3a** in a yield of 61% isolated (Table 1, entry 5). The target compound **3a** was produced by the isooctane in a 44% isolated yield (Table 1, entry 8). Unfortunately, 1,4-Dioxane, CH_3_CN, DMF, ethanol and DMSO cannot be used in this reaction, product **3a** was not being found.

**Table 1:** Effect of the solvent on lipase-catalyzed transesterification of methyl 1H-pyrrole-2-carboxylate with benzyl alcohol in *n*-Hexane

Entry	Lipase	Solvent	**3a** (%)
1	Novozym 435	Toluene	46
2	Novozym 435	1,4-Dioxane	0
3	Novozym 435	CH_3_CN	0
4	Novozym 435	DMF	0
5	Novozym 435	*n*-Hexane	61
6	Novozym 435	Ethanol	0
7	Novozym 435	DMSO	0
8	Novozym 435	Isooctane	44

Reaction conditions: methyl 1*H*-pyrrole-2-carboxylate **1a** (1.0 mmol), benzyl alcohol **2a** (0.2 mmol), 60 mg Novozym 435, 10 mL solvent, 1.0 g molecular sieves, 40 °C, 150 rpm, 24 h

### Effect of the lipase load on lipase-catalyzed transesterification

**Fig. 2:**
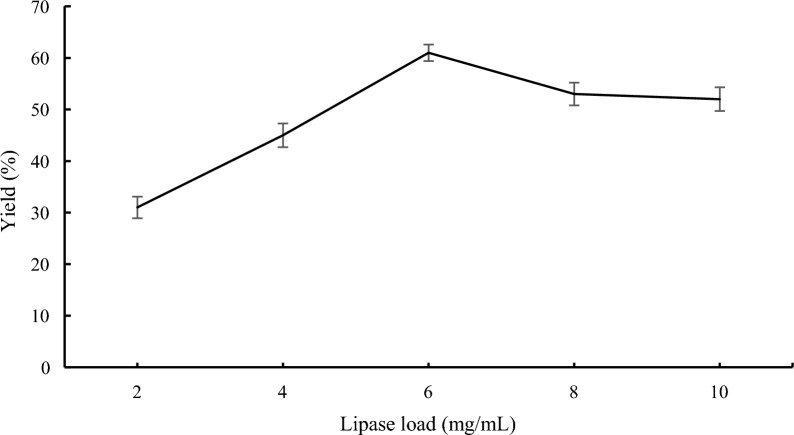
Effect of the lipase load on lipase-catalyzed transesterification. Reaction conditions: methyl 1*H*-pyrrole-2-carboxylate **1a** (1.0 mmol), benzyl alcohol **2a** (0.2 mmol), 1.0 g molecular sieve, 150 rpm, 40 °C, 24 h, *n*-Hexane (10 mL)

For an industrial application to be effective, lipase load is a critical component. High lipase load may shorten reaction time and increase the reaction rate. Nevertheless, the cost of lipase increased as its supply increased and was high. So, the impact of Novozym 435 load from 2 to 10 mg/mL on the composition of compound **3a** was evaluated under 5:1 (**1a**/**2a**) reactant molar ratio, stirrer speed of 150 r/min, molecular sieves of 1 g, 40 °C reaction temperature, and a 24 h reaction time in *n*-Hexane. It can be seen from the Fig. 2 that the transformation rose from 31 to 61% when the catalyst amount was raised from 2 to 6 mg/mL. If the lipase load was from 8 to 10 mg/mL, the amount of compound **3a** was slightly reduced. The lipase aggregation at the reaction interface reduced the effective lipase concentration and indeed the region of electrode contact area, may be the origin of this occurrence [34]. The ideal lipase load for Novozym 435 was determined to be 6 mg/mL after considering the price of lipase and the presence of component **3a**.

### Effect of the molecular sieves on lipase-catalyzed transesterification

**Fig. 3:**
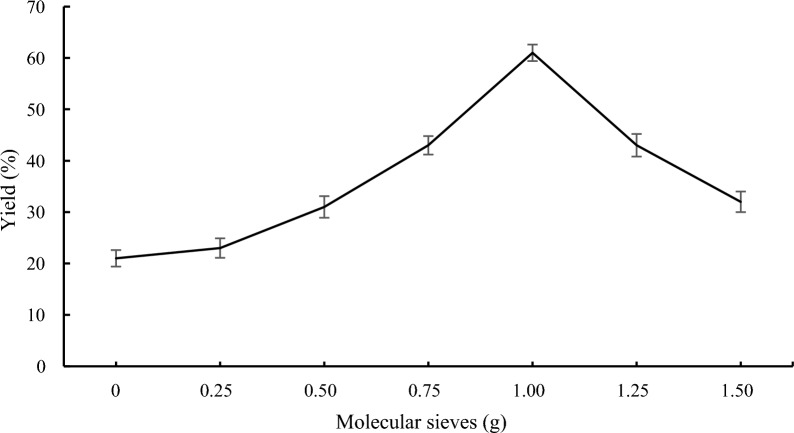
Effect of the molecular sieves on lipase-catalyzed transesterification. Reaction conditions: methyl 1*H*-pyrrole-2-carboxylate **1a** (1.0 mmol), benzyl alcohol **2a** (0.2 mmol), 60 mg Novozym 435, *n*-Hexane (10 mL), 40 °C, 150 rpm, 24 h

The reaction mixture including alcohol and methyl 1H-pyrrole-2-carboxylate was made using dehydrated n-hexane. Therefore, the effect of molecular sieves from 0 to 1.5 g on the content of compound **3a** was investigated. As shown in Fig. 3, the conversion increased from 22 to 61% when the molecular sieves were raised from 0 to 1.0 g. The synthesis of pyrrole ester then gradually decreased when the molecular sieve concentration was raised further. The addition of molecular sieves often increases the conversion of the equilibrium [35]. However, there have also been several reports of unfavorable consequences, such as the breakdown of unstable compounds [36, 37].

### Effect of the molar ratio on lipase-catalyzed transesterification

**Fig. 4:**
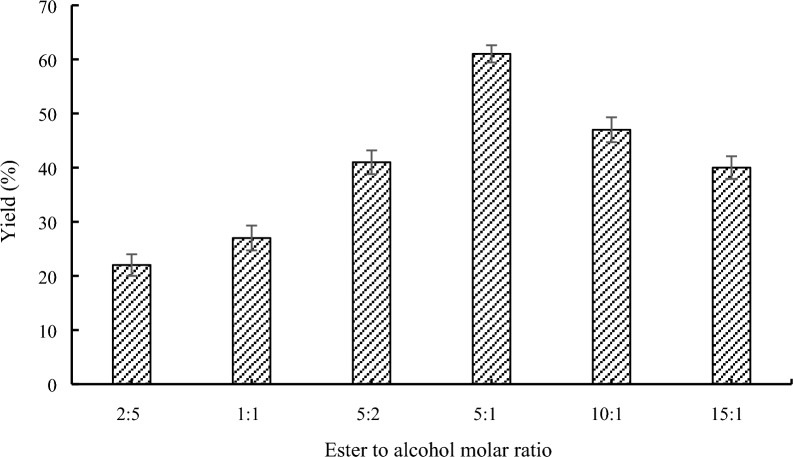
Effect of the molar ratio on lipase-catalyzed transesterification. Reaction conditions: 60 mg Novozym 435, 1.0 g molecular sieve, 40 °C, 150 rpm, 24 h, *n*-Hexane (10 mL)

The influence of substrate molar ratio was frequently cited as a notable feature in the behavior of enzymes involved in synthesis [38]. Hence, at various molar ratios (**1a**/**2a**), the transesterification of methyl 1*H*-pyrrole-2-carboxylate (**1a**) with benzyl alcohol (**2a**) was investigated. Figure 4 shows the impact of molar ratio on the transesterification of **1a** with **2a**. The yield of compound **3a** was 22% at the molar ratio of 2:5. After changing the molar ratio was adjusted from 2:5 to 5:1, the production of compound **3a** raised from 22 to 61%, demonstrating that an increase in **1a** might cause the reaction thermodynamic equilibrium to move in favor of **3a** pyrrole ester, and enhance the iterate of **1a**. The 5:1 molar ratio, which had the greatest conversion rate, was determined as the best molar ratio for further research. But with the subsequent rise in the molar ratio to 10:1 and 15:1, the yield dropped slightly to 47% and 40%, respectively. This is so that when the molar ratio reached a 5:1 ratio, the reactions equilibria would be equivalent. As a result, a rise in **1a** may cause the reaction equilibrium to shift in the other way, decreasing conversion [39]. Consequently, the following tests were optimized using a molar ratio of 5:1.

### Effect of the reaction time on lipase-catalyzed transesterification

**Fig. 5:**
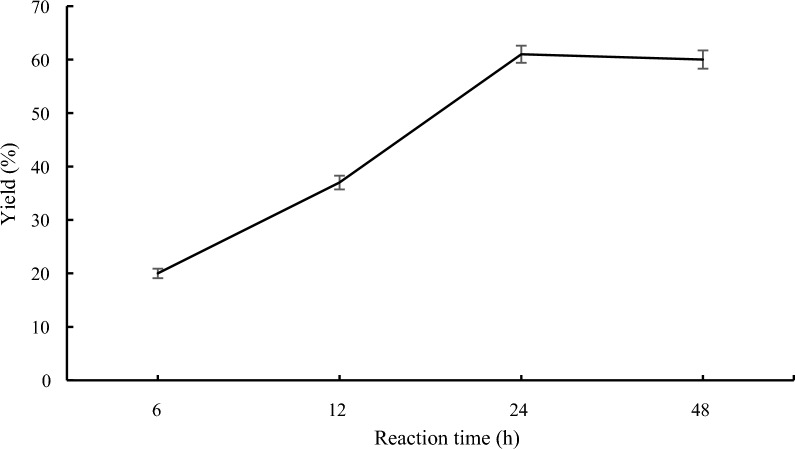
Effect of the reaction time on lipase-catalyzed transesterification. Reaction conditions: methyl 1*H*-pyrrole-2-carboxylate **1a** (1.0 mmol), benzyl alcohol **2a** (0.2 mmol), 60 mg Novozym 435, 1.0 g molecular sieve, 40 °C, 150 rpm, *n*-Hexane (10 mL)

The optimum ester synthesis was time-dependent, as originally described. The yield of ethyl ferulate under Novozym’s catalysis steadily rose as the reaction period was extended [39]. In spite of this, the hydrogel-bound lipase of *P. aeruginosa* MTCC-4713 generated the greatest amount of ester during the 6 h synthesis of methyl acrylate, before steadily declining after that [40]. Therefore, the transesterification of pyrrole ester with benzyl alcohol was studied at different reaction time. It can be seen from the Fig. 5, when the reaction time was raised from 6 to 24 h, the translation improved from 20 to 61%. The yield significantly decreased to 60% with the additional increase in reaction time to 48 h. Therefore, the reaction time for the ideal reaction circumstances was determined to be 24 h.

### Effect of the temperature on lipase-catalyzed transesterification

**Fig. 6:**
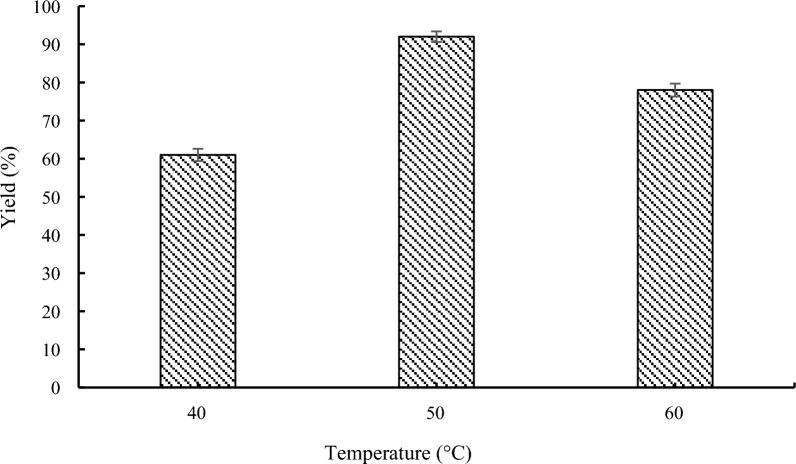
Effect of the temperature on lipase-catalyzed transesterification. Reaction conditions: methyl 1*H*-pyrrole-2-carboxylate **1a** (1.0 mmol), benzyl alcohol **2a** (0.2 mmol), 60 mg Novozym 435, 1.0 g molecular sieve, 150 rpm, 24 h, *n*-Hexane (10 mL)

Lipase activity and reaction rate were both impacted by reaction temperature. Although increasing temperature sped up reaction rate and lowered reaction time, but it also decreased lipase activity [28]. As shown in Fig. 6, the translation rate has risen from 61 to 92% as the temperature rose from 40 to 50 °C. When the temperature rose from 50 to 60 °C, the conversion was dramatically reduced. The yield of compound **3a** was 78% at 60 °C. This suggested that because of the lengthy reaction time at high temperatures, the enzyme may undergo thermal denaturation. For the progressively advanced, the reaction temperature was maintained at 50 °C to prevent thermal inactivation of the enzyme and to achieve high conversion.

### Effect of the agitation speed on lipase-catalyzed transesterification

**Fig. 7:**
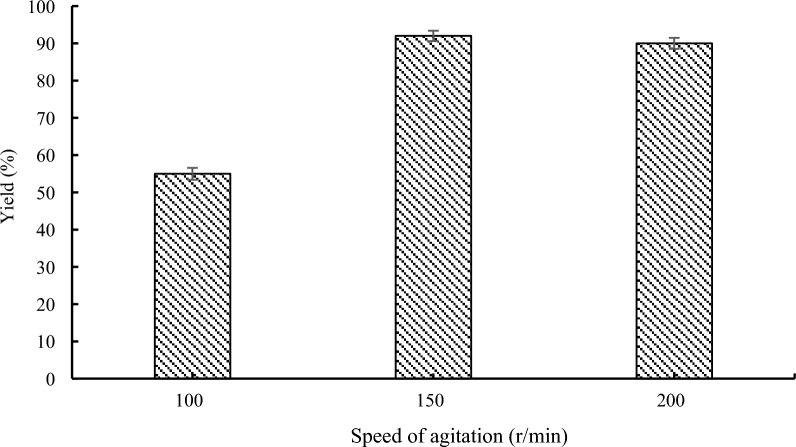
Effect of the agitation speed on lipase-catalyzed transesterification. Reaction conditions: methyl 1*H*-pyrrole-2-carboxylate 1a (1.0 mmol), benzyl alcohol 2a (0.2 mmol), 60 mg Novozym 435, 1.0 g molecular sieve, 50 °C, 24 h, n-Hexane (10 mL)

Reaction time and rate were altered by appropriate agitation occurring at an ideal pace. Experiments were conducted at agitation rates ranging from 100 to 200 rpm to evaluate the impact of agitation speeds. As shown in Fig. 7, when converting between 100 and 150 rpm, the conversion progressively rises from 55 to 92%. The layer surrounding the solid lipase particles was reduced with an increase in agitation speed, which reduced the mass transfer resistance. Even yet, the speed was only slightly reduced when it was raised to 200 rpm. Since there was only a slight difference between stirrer speeds of 150 and 200 rpm, 150 rpm was chosen to minimize energy usage.

### Reusability of enzymes

**Fig. 8:**
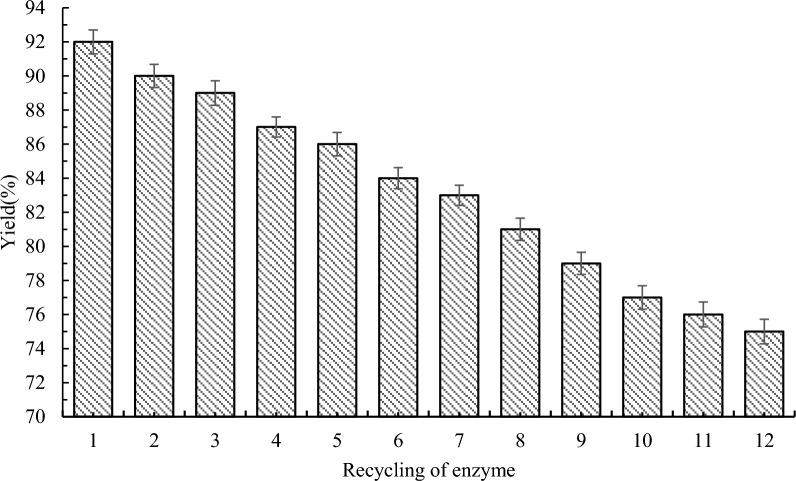
Reusability of enzymes. Reaction conditions: methyl 1*H*-pyrrole-2-carboxylate **1a** (1.0 mmol), benzyl alcohol **2a** (0.2 mmol), 60 mg Novozym 435, 1.0 g molecular sieve, 50 °C, 150 rpm, 24 h, *n*-Hexane (10 mL)

It’s common knowledge that one of the most important advantages or benefits of lipases is the reusability of enzymes. In this part, the product **3a** was continuous obtained by using lipase Novozym 435, which we can analyze its economic viability and potential for reuse. Then we used filter paper to collect enzyme particles under the optimized conditions. After that, *n*-Hexane was used to clean the enzyme particles. Recycling enzyme particles were utilized to synthesize **3a** after drying. The recycling of Novozym 435 in the esterification process was studied by reusing the immobilized enzyme in 12 cycles in *n*-Hexane at 50 °C. And the experiment was repeated three times. As shown in Fig. 8, the bar chart indicated that the yield of **3a** was reduced with the times of increasingly using by Novozym 435. Interestingly, we found that the yield of desired product **3a** was still at 75%, even though Novozym 435 had been used 12 cycles. Thus, the using of enzymatic esterification process shows excellent economic property and potential for reutilizing.

### Substrate scope

With the optimized circumstances, we assessed the scope of the substrate. It can be seen from the Table 2 that different aromatic and primary alcohols (**2b**–**2o**) were chosen to react with (**1a**) with optimal reaction conditions. With moderate to good yields of 75–92%, the reaction of **1a** and benzylic alcohols having alkyl, methoxy and halo substituents at the *o-*, *m-*, and *p-* positions led to the formation of the desired compounds **3a**–**3h**. Unfortunately, the reaction of **1a** and **2d** *p*-nitrobenzyl alcohol cannot give product **3d**. The yield of products **3c**, **3d 3f** and **3g** with electron withdrawing substituents on the benzene ring were lower than that of products **3a**, **3b** and **3e** with electron donating substituents. It might be electron withdraw groups have a negative on reaction yield. Importantly, (R)-( +)-1-phenylethanol reacted smoothly with **1a**, 52% of the target product, **3i**, was successfully created. But the yield of **3i** was lower than **3a**, it might because the spatial hindrance of alpha-substituted benzyl alcohols resulted low yield. Gratifyingly, the reaction could also be applied to a range of cyclic and acyclic aliphatic alcohols with **1a**, which gave products **3j**–**3n** with 72–98% isolated yields. In addition, methallyl alcohol was well tolerated, giving **3o** in 71% yield. It is interesting that when methyl 1*H*-indole-2-carboxylate (**1b**) and benzyl alcohol were combined, the desired product **3p** was produced in 81%.

**Table 2:**
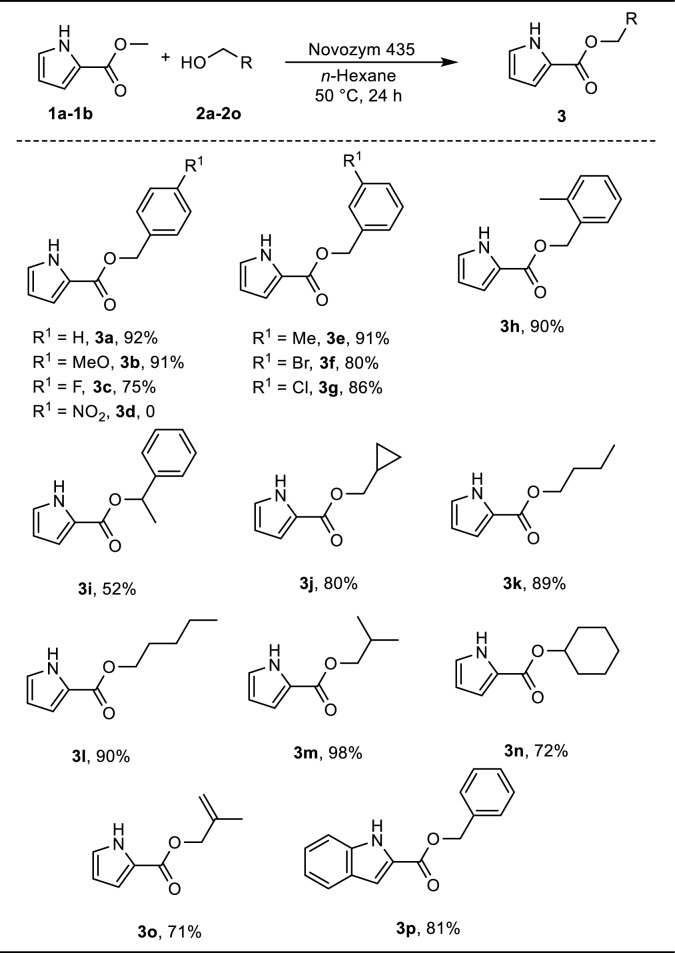
Substrate scope of alcohols for transesterification reactions

Reaction conditions: methyl 1*H*-pyrrole-2-carboxylate **1a** (1.0 mmol), benzyl alcohol **2a** (0.2 mmol), Novozym 435 60 mg, 1.0 g molecular sieves, and *n*-Hexane (10 mL) for 24 h, 50 °C

**Scheme 1:**
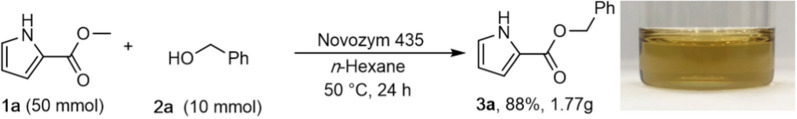
Gram scale transesterification

As shown in Scheme 1, large-scale (10 mmol) experiments between methyl 1*H*-pyrrole-2-carboxylate **1a** (50 mmol, 6.25 g) and benzyl alcohol **2a** (10 mmol, 1.08 g) were conducted to further evaluate the superlative scalability of this established method. These experiments produced the desired **3a** with an excellent yield of 88%. (1.77 g).

### The thermal behaviors of compounds

**Fig. 9:**
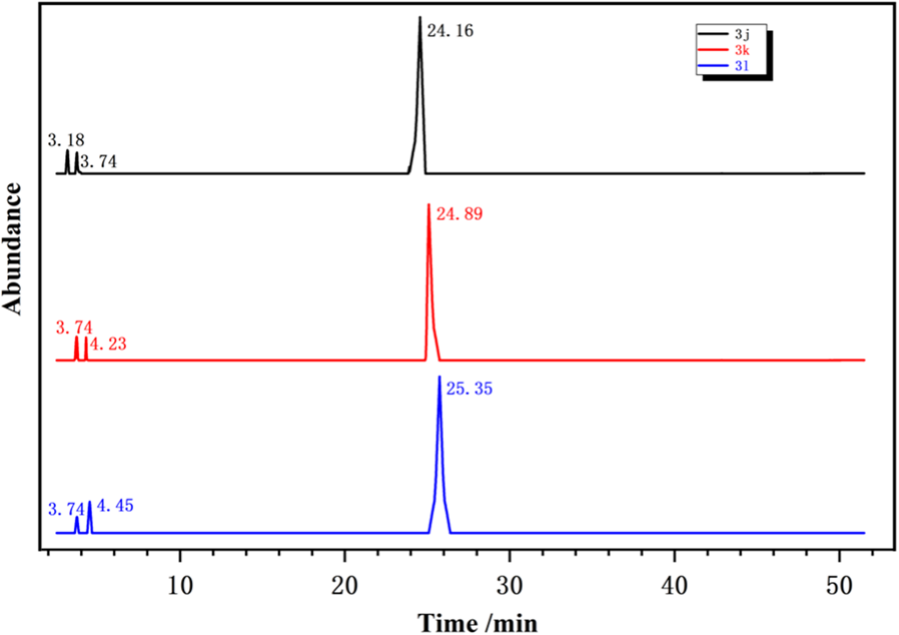
Pyrolysis-GC/MS total ion chromatogram of **3j**, **3k** and **3l** at 30–900 °C

As shown in Table 3, compounds **3j**, **3k** and **3l** possessed strong characteristic aroma such as sweet, herbs, fruity and acid. Studying the chemicals produced under heated conditions was therefore also crucial. The pyrolysis conditions included an oxidizing atmosphere (91% nitrogen, 9% oxygen) and temperatures between 30 and 900 °C. Furthermore, the pyrolysis gas chromatograms obtained under influence of heat are shown in Fig. 9 and the peak position areas of the pyrolysis products are listed in Table 4. As shown in Table 4, the evaporative yield of compound **3j** in an oxidizing environment at high temperatures is 96.48%. Compound **3h** was also the most stable material tested, releasing just 3.52% of its breakdown at high temperatures in oxidizing environments. When the volatile pyrolysis product of **3j** was examined, the main byproducts were cyclopropyl carbinol (1.97%) and pyrrole (1.55%). Under extreme heat in an oxidizing atmosphere, the evaporative yields of compound **3k** and compound **3l** are 95.15% and 94.93%. Pyrrole (1.87%) and 1-butanol (2.98%) made up most of the pyrolysis products of **3k**. Pyrole (1.91%) and 1-pentanol (3.16%) made up most of the pyrolysis products of **3l**. It is important to note that several pyrolysis byproducts of the compound **3k** and **3l** contained the distinguishable flavour components, including pyrrole, 1-butanol and 1-pentanol. They are all appropriate to use as flavor and aroma mediators. For more characterization data of compounds see Additional file 1.

**Table 3:** Odor description of compounds

Entry	Compound	Odor description
1	benzhydryl 1*H*-pyrrole-2-carboxylate (**3j**)	Sweet, acid
2	butyl 1*H*-pyrrole-2-carboxylate (**3k**)	Sweet, herbs, acid
3	pentyl 1*H*-pyrrole-2-carboxylate (**3l**)	Sweet, fruity, acid

**Table 4:** Pyrolysis products of **3j**, **3k** and **3l** (TIC area %)

Sample	Time	Compound	Area %
benzhydryl 1*H*-pyrrole-2-carboxylate (**3j**)	3.18	Cyclopropyl carbinol	1.97
	3.74	pyrrole	1.55
	24.16	**3j**	96.48
butyl 1*H*-pyrrole-2-carboxylate (**3k**)	3.74	pyrrole	1.87
	4.23	1-butanol	2.98
	24.89	**3k**	95.15
pentyl 1*H*-pyrrole-2-carboxylate (**3l**)	3.74	pyrrole	1.91
	4.45	1-pentanol	3.16
	25.35	**3l**	94.93

**Fig. 10:**
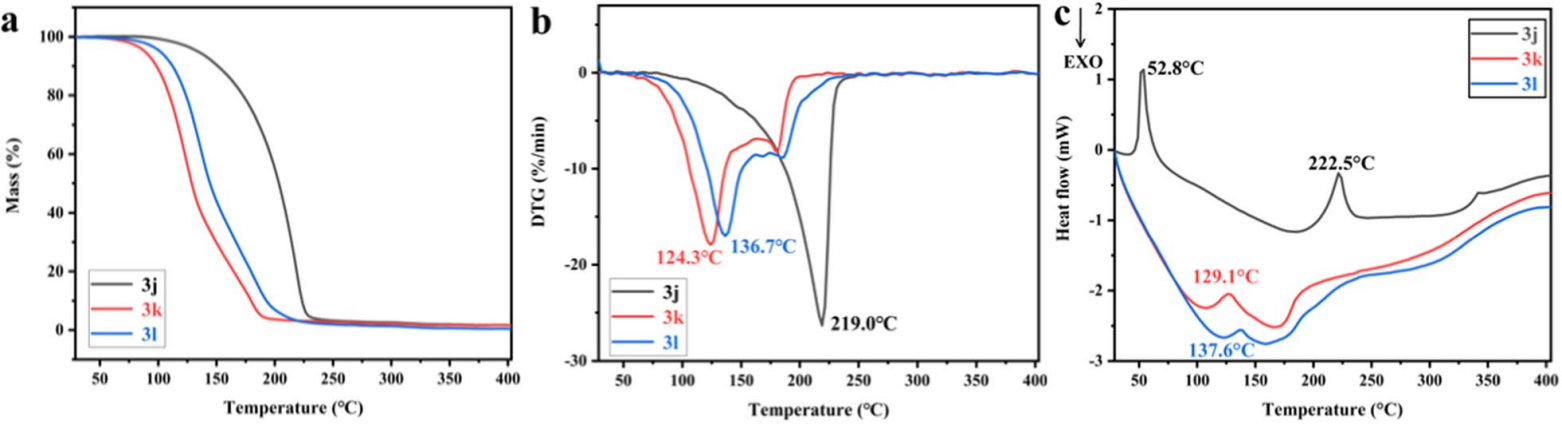
Thermal degradation curve of compounds **3j**, **3k** and **3l**, **a** TGA, **b** DTG and **c** DSC

Figure 10a, b shows the TG, DTG, and DSC profiles of the title compounds with 30 and 400 °C at a rate of heating of 10 °C min^−1^. Compounds **3j**, **3k**, and **3l** decomposed at conditions varying from 70.9 to 250.0, 62.2 to 220.0, and 65.7 to 230.0 °C, respectively, as shown in Fig. 10a, b. And it shows that **3k** and **3l** had the greatest rate of disintegration at 124.3 and 136.7 °C with starting mass reductions of 41.92% and 38.16%. The samples then showed a consistent and uniform mass loss pattern, with overall weight losses of 98.43% and 99.65%. Most of the thermal mass loss (80.42%) occurred at 219.0 °C, with an ultimate total weight loss of 98.41%, based to both the TG and DTG curves of **3j**. Due to the chemical structure of **3j** includes cyclopropyl while the structures of **3k** and **3l** contain straight-chain carbon, most of the thermal mass loss in **3j** was higher than in **3k** and **3l**, which may have been due to distinct stabilizations. These three compounds were found to be steady at ambient temperature by the TG and DTG testing.

Figure 10c shows the DSC individuals for **3j**–**3l** that were examined in an environment of air. Equipment was used to capture the highest temperature of DSC curves and the change in enthalpy of the samples. The T_onset_, T_peak_, and T_end_ temperatures were similar to those discovered using DTG graphs. With the DSC graph of **3j**, there is a clear, significant endothermic peak at 52.8 °C. The sharp peak indicates a melting-point of **3j**, as there is no weight loss at 52.8 °C, on the other hand. Additionally, it demonstrated that the T_peak_ of **3j**–**3l** in DSC curves occurred in the major mass loss areas there, suggesting that the samples may have vaporized or broken down during the endothermic phase. Overall, the DSC and TG-DTG results agreed with the other and showed that the samples were steady at room temperature. The information regarding the outcomes based on the graphs shown in Table 5.

**Table 5:** Thermal analysis data of compounds **3j**, **3k** and **3l**

Compound	DSC					TG-DTG		Mass loss/%
	T_melt_/°C	T_onset_/°C	T_peak_/°C	T_end_/°C	∆H/kJ mol^−1^	T_p_/°C	T_range_/°C	
**3j**	52.8	206.9	222.5	229.2	92.6	219.0	70.9–250.0	98.41
**3k**	–	111.5	129.1	144.5	54.4	124.3	62.2–220.0	98.43
**3l**	–	124.5	137.6	146.9	25.81	136.7	65.7–230.0	99.65

## Conclusion

In conclusion, a simple enzymatic approach to synthesis pyrrole esters with commercially accessible alcohols was developed. The effects of lipase type, solvent, lipase load, molecular sieves, reaction time, substrate molar ratio, reaction temperature and speed of agitation on the amount of product were investigated to give optimal reaction conditions. The optimized conditions were set as molar ratio of 5:1 (methyl 1*H*-pyrrole-2-carboxylate/ benzyl alcohol, **1a**/**2a**), Novozym 435 of 6 mg/mL, stirrer speed of 150 r/min, molecular sieves of 1 g, 50 °C reaction temperature, and a 24 h reaction time in *n*-Hexane leading to a high yield of 92%. A wide variety of alcohols are well tolerated during the transformation using Novozym 435, and the characterization of their structure was determined by ^1^H NMR, ^13^C NMR, HRMS, and IR. The findings of the odor study revealed that compounds **3j**, **3k**, and **3l** exhibit scents that are typically distinct from those of the related methyl 1*H*-pyrrole-2-carboxylate and alcohols, including sweet, herbal, fruity, and acidic notes. The Py–GC/MS was used to examine the pyrolysis byproducts of the three flavoring compounds **3j**, **3k**, and **3l** in oxidative environments. The evaporative yields of compounds **3j**,** 3k** and **3l** were 96.48%, 95.15% and 94.93%, respectively. The TG analysis result showed that the main mass change stage of **3j**,** 3k** and **3l** were between 62.2 and 250.0 °C with mass decreased by 98.41%, 98.43% and 99.65% of the total mass, respectively. As seen by the T_peak_ of **3j**,** 3k** and **3l** in DSC curves, either evaporation or breakdown of the compounds **3j**,** 3k** and **3l** took place during in the thermal decomposition phase. They have good thermal stability under certain temperature.

### Supplementary Information

**Additional file 1:** 1. Experimental. 2. Optimization details. 3. General procedure for the transesterification. 4. Characterization data for the transesterification products. 5. Reference. 6. Copy of ^1^H and ^13^C NMR spectra of compounds **3a**-**3p**. 7. Copy of Gas chromatography-mass spectrometry ion flow chromatograms of compounds **3a**-**3p**.

## Data Availability

All data generated or analyzed during this study are included in this published article.

## Abbreviations

GC–MS–O: Gas chromatography–mass spectrometry–olfactometry
Py–GC/MS: Pyrolysis–gas chromatography/mass spectrometry
TG: Thermogravimetry
DSC: Differential scanning calorimeter
CRL: *Candida Rugosa* lipase
TLIM: *Thermomyces lanuginosus*, and immobilized on a non-compressible silica gel carrier
DMF: N,N-dimethylformamide
DMSO: Dimethyl sulfoxide
NMR: Nuclear magnetic resonance
