# Supplementary material for: Enzymatic synthesis of novel pyrrole esters and their thermal stability
Source: BMC Chem. 2023 Sep 23;17(1):123. doi: 10.1186/s13065-023-01039-5 (PMC10518093; doi:10.1186/s13065-023-01039-5)
Supplement: Supplementary file 1 — Additional file 1: 1. Experimental. 2. Optimization details. 3. General procedure for the transesterification. 4. Characterization data for the transesterification products. 5. Reference. 6. Copy of 1H and 13C NMR spectra of compounds 3a-3p. 7. Copy of Gas chromatography-mass spectrometry ion flow chromatograms of compounds 3a-3p. [file 13065_2023_1039_MOESM1_ESM.docx]

**Additional file**

**Enzymatic synthesis of novel pyrrole esters and their thermal stability**

Jingyi Hu ^1^, Meng Zhou ^1^, Yujie Zhang ^2^, Xi Zhang ^3^, Xiaoming Ji ^1^, Mingqin Zhao ^1^

and Miao Lai [[1]](#footnote-1), *

^1^ Flavors and Fragrance Engineering & Technology Research Center of Henan Province, College of Tobacco Science, Henan Agricultural University, Zhengzhou 450002, China

*^2^ Technology Center, China Tobacco Hebei Industrial Co.,Ltd., Shijiazhuang, 050051, China*

^3^ Technology Center, China Tobacco Shanxi Industrial Co., Ltd., Xian 710065, China

laimiao@henau.edu.cn

Table of Contents

[1. Experimental 1](#_Toc142559646)

[2. Optimization details 2](#_Toc142559647)

[3. General procedure for the transesterification 3](#_Toc142559648)

[4. Characterization data for the transesterification products 3](#_Toc142559649)

[5.Reference 7](#_Toc142559650)

[6. Copy of ^1^H and ^13^C NMR spectra 9](#_Toc142559651)

[7. Copy of Gas chromatography-mass spectrometry ion flow chromatograms 23](#_Toc142559652)

# 1. Experimental

**1.1 General information**

Lipozyme TLIM (*Thermomyces lanuginosus*, and immobilized on a non-compressible silica gel carrier) and Novozym 435 (*Candida antarctica* lipase B immobilized on an acrylic resin-based hydrophobic carrier). *Candida Rugosa* lipase were purchased from Novozymes A/S. The Shanghai Meryer Chemical Technology Co., Ltd. (China) was the source of all the commercial-grade chemicals and solvents employed in this work. ^1^H NMR and ^13^C NMR spectra data were recorded by a BRUKER AVANCE III 400 MHz spectrometer (^1^H 400 MHz, ^13^C 100 MHz), using CDCl_3_ as the solvent with tetramethylsilane (TMS) as the internal standard at room temperature. ^1^H NMR spectral data are given as chemical shifts in ppm followed by multiplicity (s- singlet; d- doublet; t- triplet; q- quartet; m- multiplet), number of protons and coupling constants. ^13^C NMR chemical shifts are expressed in ppm. Infrared spectra were recorded with a Thermo Scientific Nicolet 6700 FT-IR Spectrometer. HRMS data were obtained using AB SCIEX Triple TOF 5600+ high resolution mass spectrometer (USA). The products listed below were determined by ^1^H and ^13^C NMR spectra. Melting points were determined using melting point X-4 (Gongyi Kerui) apparatus.

**1.2 Synthesis of target compounds**

Methyl 1H-pyrrole carboxylate (1.0 mmol), corresponding alcohols (0.2 mmol), lipase (60 mg) and molecular sieves (3A, 1.0 g) were added to a 50 mL tube that had a magnetic stirring bar at air condition. The mixture was then given 10 mL of *n*-Hexane, and it was stirred for 24 h in constant temperature water bath oscillator (Honour Instrument Co., Ltd, Tianjin) at 50 °C. 10 mL of ethyl acetate was incorporated into the reaction mixture after cooling, and it was then washed with 10 mL of brine. Ethyl acetate was used to extract the aqueous layer twice. The combined organic solvent was filtered, concentrated in *vacuo*, and dried over anhydrous Na_2_SO_4_. The residue was purified to obtain products ranging from 52% to 98% yields using flash spectrophotometric method using zeolite with petroleum ether-EtOAc as the extraction solvent. In general, ^1^H and ^13^C NMR spectroscopy, HRMS and IR were used to establish the identities and purity of compounds. The supporting material file contains the remaining spectrum and spectral data.

**1.3 Sensory panel**

The sensory panels were chosen in accordance with the method by Feng [^[1]^](#reference). Five sane, non-smoking judges, two men and three women, all between the ages of 23 and 29, made up the panel. Members of the panel are all from Flavors and Fragrance Engineering & Technology Research Center of Henan Province. All of the panelists had undergone in-depth training and had competence evaluating different cuisines and flavors using their perceptions. And each participant had more than 100 hours of sensory experience rating the flavor of various substances. The assessors received training by identifying and characterizing the aroma characteristics of common odorants. The panelist was asked to describe the smell verbally when the peak emerged at the GC (Agilent, 7890B-5977A) at the same time. Prior to the publishing of this study, all participants provided their written, informed consent. GC-MS-O analysis was carried out using an Agilent 7890B-5977A with a capillary column DB-WAX (30 m × 0.25 mm × 0.25 μm) and a sniffing port (Gerstel OP3, Germany). The analytical conditions were as follows: the oven temperature was programmed from 50 °C to 280 °C at a rate of 10 °C min^-1^, then held at 280 °C for 5 min; carrier gas, helium; flow rate, 1.6 mL min^-1^; electron ionization, 70 eV; ion source temperature, 230 °C. The column effluent was divided (ratio 1:1) between the MSD detector and the sniffing port through one Y-shaped glass splitter. The effluent to the sniffing port was enclosed with a stream of humidified air of 16 mL min^-1^ and transferred to the glass detection cone by one length of capillary column at the temperature of 250 °C. All the samples with a concentration of 5 wt.% in CH_2_Cl_2_, and the injection volume was 1.0 μL.

**1.4 Py-GC/MS method**

The GC/MS (Agilent, 7890A/5975C) was used in collaboration with the Pyroprobe 5250T (CDS, Analytical Inc.). Py-GC/MS analysis used in this study has a Pyroprobe connected directly to a GC/MS (Agilent, 7890A/5975C). About 0.20 mg of each sample was centered in a 25 mm quartz tube and heated nominally at the rate of 30 °C s^-1^. The sample was pyrolyzed non-isothermally at 30-900 °C for 10 s. The chromatographic separation was performed using a DB-5MS fused silica capillary column (30 m × 250 μm × 0.25 μm, Agilent). The injector temperature was kept at 300 °C. Initial oven temperature was set at 50 °C, then heated to 80 °C at the rate of 6 °C min^-1^, followed by a heating rate of 2 °C min^-1^ to 110 °C with 2 min and finished at 250 °C with a rate of 5 °C min^-1^, held there for 2 min. Helium at a constant flow rate of 1 mL min^-1^ was used as the carrier gas and the split ratio was 20:1. The separated compounds were analyzed by the mass spectrometer. The EI ionization energy was 70 eV, and the transfer line temperature was 300 °C. Ion source temperature was 230 °C and quadrupole temperature was 150 °C. The mass spectra were obtained from m/z 30 to 500, and solvent delay time was 3.9 min.

**1.5 TG analysis**

TG-DTG and differential scanning calorimeter (DSC) curves of the target compounds were detected by a simultaneous thermal analyzer (STA 449 F3, Netzsch, Germany). Every compound was preserved about 5 mg, and spectrally pure Al_2_O_3_ served as the standard. Each experiment was run in an air-conditioned environment at a flow rate of 60 mL min^-1^ and heated at a rate of 10 °C min^-1^ between 30 and 400 °C.

# 2. Optimization details

Under air atmosphere, methyl 1H-pyrrole-2-carboxylate **1a** (1.0 mmol), benzyl alcohol **2a** (0.2 mmol) and lipase (60 mg) were charged into a 50 mL sealable tube equipped with a magnetic stirring bar. After the addition of *n*-Hexane (10.0 mL), the resulting mixture was stirred at 50 °C for 24 h in in constant temperature water bath oscillator. After cooling down, the reaction mixture was diluted with 10 mL ethyl acetate and washed with 10 mL brine. The aqueous layer was extracted twice with ethyl acetate. The combined organic phase was dried over anhydrous Na_2_SO_4_, filtered and concentrated in *vacuo*. The residue was purified by flash column chromatography on silica gel (eluent: petroleum ether-EtOAc) to give the pure product **3a**.

# 3. General procedure for the transesterification

Under air atmosphere, pyrrole ester **1** (1.0 mmol), corresponding alcohols **2** (0.2 mmol, 1.0 equiv.) and lipase (60 mg) were charged into a 50 mL sealable tube equipped with a magnetic stirring bar. After the addition of *n*-Hexane (10.0 mL), the resulting mixture was stirred at 50 °C for 24 h in constant temperature water bath oscillator. After cooling down, the reaction mixture was diluted with 10 mL ethyl acetate and washed with 10 mL brine. The aqueous layer was extracted twice with ethyl acetate. The combined organic phase was dried over anhydrous Na_2_SO_4_, filtered and concentrated in *vacuo*. The residue was purified by flash column chromatography on silica gel (eluent: petroleum ether-EtOAc) to give the pure product **3** in moderate to good yields.

**Gram scale** **transesterification reaction:** Under air atmosphere, methyl 1H-pyrrole-2-carboxylate **1a** (50 mmol, 6.25 g), benzyl alcohol **2a** (10 mmol, 1.08 g), and lipase (300 mg) were charged into a 100 mL pressure tube equipped with a magnetic stirring bar. After the addition of *n*-Hexane (50.0 mL), the resulting mixture was stirred at 50 °C for 24 h in constant temperature water bath oscillator. After cooling down, the reaction mixture was diluted with ethyl acetate and washed with brine. The aqueous layer was extracted twice with ethyl acetate. The combined organic phase was dried over anhydrous Na_2_SO_4_, filtered and concentrated in *vacuo*. The residue was purified by flash column chromatography on silica gel (eluent: petroleum ether-EtOAc) to give the pure product **3a** in 88% yield (1.77 g).

# 4. Characterization data for the transesterification products

**benzyl 1H-pyrrole-2-carboxylate (3a)^2^**

Isolated as a yellow liquid, 37 mg, 92% yield; ^1^H NMR (400 MHz, CDCl_3_) δ 9.29 (s, 1H), 7.47 – 7.40 (m, 2H), 7.40 – 7.35 (m, 2H), 7.33 (ddd, *J* = 7.0, 3.4, 1.5 Hz, 1H), 7.00 – 6.95 (m, 1H), 6.95 – 6.91 (m, 1H), 6.26 (dt, *J* = 3.6, 2.6 Hz, 1H), 5.31 (s, 2H); ^13^C NMR (100 MHz, CDCl_3_) δ 161.01, 136.18, 128.59, 128.23, 128.18, 123.11, 122.59, 115.67, 110.54, 66.01; HRMS (ESI) calcd. for C_12_H_12_NO_2_: [M+H]^+^: 202.0868, found: 202.0869. CAS Number: 35889-87-3.

**4-methoxybenzyl 1H-pyrrole-2-carboxylate (3b)**

Isolated as a yellow liquid, 40.6 mg, 88% yield; ^1^H NMR (400 MHz, CDCl_3_) *δ* 9.14 (s, 1H), 7.36 (d, *J* = 8.68 Hz, 2H), 6.98-6.92 (m, 2H), 6.91-6.84 (m, 2H), 6.25 (q, *J* = 6.16, 2.72 Hz, 1H), 5.24 (s, 2H), 3.81 (s, 3H).; ^13^C NMR (100 MHz, CDCl_3_) *δ* 160.97, 159.65, 130.09, 128.27, 122.84, 122.75, 115.52, 113.96, 110.50, 65.86, 55.31. IR (KBr) ν_max_ 3361 (pyrrole), 2987, 2956, 2921, 1781 (O=C-O), 1615, 1599, 1570, 1275 (CH₃O-) and 791 (phenyl) cm^-1^; HRMS (ESI) calcd. for C_13_H_13_NO_3_Na: [M+H]^+^: 232.0974, found: 232.0972. CAS Number: 1881320-48-4.

**4-fluorobenzyl 1H-pyrrole-2-carboxylate (3c)**

Isolated as a yellow solid, m. p. 53.2 – 54.3 ^o^C, 33 mg, 75% yield; ^1^H NMR (400 MHz, CDCl_3_) δ 9.36 (s, 1H), 7.39 (dd, *J* = 8.2, 5.6 Hz, 2H), 7.05 (t, *J* = 8.6 Hz, 2H), 6.95 (d, *J* = 2.5 Hz, 2H), 6.33 – 6.18 (m, 1H), 5.27 (s, 2H); ^13^C NMR (100 MHz, CDCl_3_) δ 162.65 (d, *J* = 245.3 Hz), 161.02, 132.02(d, *J* = 3.3 Hz), 130.18 (d, *J* = 8.2 Hz), 123.30, 122.44, 115.75, 115.50 (d, *J* = 21.3 Hz), 110.57, 65.31. IR (KBr) ν_max_ 3435, 2907, 1772, 1632, 1612, 1587, 1574, 1556, and 891 cm^-1^; HRMS (ESI) calcd. for C_12_H_11_FNO_2_: [M+H]^+^: 220.0774, found: 220.0773.

**3-methylbenzyl 1H-pyrrole-2-carboxylate (3e)**

Isolated as a yellow liquid, 39 mg, 91% yield; ^1^H NMR (400 MHz, CDCl_3_) δ 9.35 (s, 1H), 7.30 – 7.23 (m, 1H), 7.21 (d, *J* = 7.7 Hz, 2H), 7.14 (d, *J* = 7.3 Hz, 1H), 6.99 – 6.95 (m, 1H), 6.95 – 6.91 (m, 1H), 6.26 (dt, *J* = 3.6, 2.6 Hz, 1H), 5.27 (s, 2H), 2.36 (s, 3H); ^13^C NMR (100 MHz, CDCl_3_) δ 161.11, 138.29, 136.10, 129.00, 128.94, 128.50, 125.28, 123.14, 122.62, 115.68, 110.49, 66.09, 21.40; HRMS (ESI) calcd. for C_13_H_14_NO_2_: [M+H]^+^: 216.1025, found: 216.1028. CAS Number: 2383442-46-2.

**3-bromobenzyl 1H-pyrrole-2-carboxylate (3f)**

Isolated as a yellow solid, m. p. 66.1 – 67.2 ^o^C, 50 mg, 90% yield; ^1^H NMR (400 MHz, CDCl_3_) *δ* 9.19 (s, 1H), 7.57 (d, *J* = 1.44 Hz, 1H), 7.46 (d, *J* = 8.00 Hz, 1H), 7.34 (d, *J* = 7.92 Hz, 1H), 7.27-7.20 (m, 1H), 6.98 (tq, *J* = 2.81, 1.44 Hz, 2H), 6.28 (td, *J* = 3.64, 2.60 Hz, 1H), 5.27 (s, 2H); ^13^C NMR (100 MHz, CDCl_3_) *δ* 160.69, 138.46, 131.31, 131.05, 130.15, 126.62, 123.27, 122.61, 122.30, 115.85, 110.67, 64.99. IR (KBr) ν_max_ 3217, 2887, 1704, 1687, 1654, 1632, 1601, 1588, and 719 cm^-1^; HRMS (ESI) calcd. for C_12_H_11_BrNO_2_: [M+H]^+^: 279.9973, found: 279.9974.

**3-chlorobenzyl 1H-pyrrole-2-carboxylate (3g)**

Isolated as a yellow solid, m. p. 72.0 – 73.0 ^o^C, 40 mg, 86% yield; ^1^H NMR (400 MHz, CDCl_3_) δ 9.42 (s, 1H), 7.41 (s, 1H), 7.34 – 7.26 (m, 3H), 7.03 – 6.97 (m, 1H), 6.97 – 6.85 (m, 1H), 6.27 (dt, *J* = 3.6, 2.6 Hz, 1H), 5.27 (s, 2H); ^13^C NMR (100 MHz, CDCl_3_) δ 160.92, 138.20, 134.46, 129.89, 128.37, 128.11, 126.11, 123.50, 122.24, 115.94, 110.63, 65.09. IR (KBr) ν_max_ 3390, 2921, 1732, 1643, 1621, 1602, 1597, 1573, and 834 cm^-1^; HRMS (ESI) calcd. for C_12_H_11_ClNO_2_: [M+H]^+^: 236.0478, found: 236.0477.

**2-methylbenzyl 1H-pyrrole-2-carboxylate (3h)**

Isolated as a yellow solid, m. p. 74.3 – 75.4 ^o^C, 39 mg, 90% yield; ^1^H NMR (400 MHz, CDCl_3_) δ 9.31 (s, 1H), 7.43 – 7.33 (m, 1H), 7.30 – 7.23 (m, 1H), 7.23 – 7.17 (m, 2H), 6.94 (ddt, *J* = 6.8, 2.7, 1.4 Hz, 2H), 6.25 (dt, *J* = 3.7, 2.6 Hz, 1H), 5.31 (s, 2H), 2.39 (s, 3H); ^13^C NMR (100 MHz, CDCl_3_) δ 161.09, 137.09, 134.08, 130.41, 129.25, 128.55, 126.04, 123.13, 122.58, 115.62, 110.51, 64.57, 19.00. IR (KBr) ν_max_ 3543, 2978, 2906, 1733, 1690, 1677, 1643, 1621, 1578, and 822 cm^-1^; HRMS (ESI) calcd. for C_13_H_14_NO_2_: [M+H]^+^: 216.1025, found: 216.1024.

**1-phenylethyl 1H-pyrrole-2-carboxylate (3i)**

Isolated as a yellow liquid, 22 mg, 52% yield; ^1^H NMR (400 MHz, CDCl_3_) δ 9.18 (s, 1H), 7.42 (dd, *J* = 5.2, 3.5 Hz, 2H), 7.39 – 7.32 (m, 2H), 7.32 – 7.26 (m, 1H), 6.98 (ddd, *J* = 3.8, 2.4, 1.5 Hz, 1H), 6.92 (td, *J* = 2.7, 1.5 Hz, 1H), 6.26 (dt, *J* = 3.7, 2.6 Hz, 1H), 6.07 (q, *J* = 6.6 Hz, 1H), 1.64 (d, *J* = 6.6 Hz, 3H); ^13^C NMR (100 MHz, CDCl_3_) δ 160.50, 141.87, 128.52, 127.84, 126.02, 123.01, 122.88, 115.34, 110.43, 72.23, 22.39; HRMS (ESI) calcd. for C_13_H_14_NO_2_: [M+H]^+^: 216.1025, found: 216.1027. CAS Number: 2384913-36-2.

**benzhydryl 1H-pyrrole-2-carboxylate (3j)^3^**

Isolated as a yellow liquid, 26 mg, 80% yield; ^1^H NMR (400 MHz, CDCl_3_) δ 9.46 (s, 1H), 6.95 (t, *J* = 2.9 Hz, 2H), 6.27 (dd, *J* = 5.8, 3.0 Hz, 1H), 4.10 (d, *J* = 7.2 Hz, 2H), 1.28 – 1.16 (m, 1H), 0.64 – 0.54 (m, 2H), 0.40 – 0.28 (m, 2H); ^13^C NMR (100 MHz, CDCl_3_) δ 161.49, 122.94, 122.90, 115.29, 110.36, 69.09, 10.02, 3.34; HRMS (ESI) calcd. for C_9_H_12_NO_2_: [M+H]^+^: 166.0868, found: 166.0872. CAS Number: 685563-23-9.

**butyl 1H-pyrrole-2-carboxylate (3k)^4^**

Isolated as a yellow solid, 30 mg, 89% yield; ^1^H NMR (400 MHz, CDCl_3_) δ 9.28 (s, 1H), 6.95 (dd, *J* = 4.0, 2.6 Hz, 1H), 6.94 – 6.87 (m, 1H), 6.26 (dd, *J* = 6.2, 2.6 Hz, 1H), 4.27 (t, *J* = 6.7 Hz, 2H), 1.71 (dt, *J* = 14.6, 6.8 Hz, 2H), 1.51 – 1.39 (m, 2H), 0.96 (t, *J* = 7.4 Hz, 3H); ^13^C NMR (100 MHz, CDCl_3_) δ 161.40, 122.99, 122.76, 115.09, 110.36, 64.21, 30.88, 19.21, 13.75; HRMS (ESI) calcd. for C_9_H_14_NO_2_: [M+H]^+^: 168.1025, found: 168.1028. CAS Number: 121530-00-5.

**pentyl 1H-pyrrole-2-carboxylate (3l)^5^**

Isolated as a yellow liquid, 33 mg, 90% yield; ^1^H NMR (400 MHz, CDCl_3_) δ 9.38 (s, 1H), 6.95 (td, *J* = 2.7, 1.5 Hz, 1H), 6.92 (ddd, *J* = 3.8, 2.4, 1.5 Hz, 1H), 6.26 (dt, *J* = 3.7, 2.6 Hz, 1H), 4.26 (t, *J* = 6.7 Hz, 2H), 1.79 – 1.67 (m, 2H), 1.47 – 1.31 (m, 4H), 0.92 (t, *J* = 7.1 Hz, 3H); ^13^C NMR (100 MHz, CDCl_3_) δ 161.45, 122.99, 122.82, 115.11, 110.34, 64.50, 28.53, 28.14, 22.36, 13.98; HRMS (ESI) calcd. for C_10_H_16_NO_2_: [M+H]^+^: 182.1181, found: 182.1177. CAS Number: 1312441-57-8.

**isobutyl 1H-pyrrole-2-carboxylate (3m)^6^**

Isolated as a yellow liquid, 33 mg, 98% yield; ^1^H NMR (400 MHz, CDCl_3_) δ 9.46 (s, 1H), 6.96 (td, *J* = 2.7, 1.5 Hz, 1H), 6.93 (ddd, *J* = 3.8, 2.4, 1.5 Hz, 1H), 6.26 (dt, *J* = 3.7, 2.6 Hz, 1H), 4.05 (d, *J* = 6.7 Hz, 2H), 2.04 (dp, *J* = 13.4, 6.7 Hz, 1H), 0.99 (d, *J* = 6.7 Hz, 6H); ^13^C NMR (100 MHz, CDCl_3_) δ 161.51, 122.94, 115.13, 110.32, 70.37, 27.96, 19.15; HRMS (ESI) calcd. for C_13_H_14_NO_2_: [M+H]^+^: 216.1025, found: 216.1024; HRMS (ESI) calcd. for C_9_H_14_NO_2_: [M+H]^+^: 168.1025, found: 168.1028. CAS Number: 864497-13-2.

**cyclohexyl 1H-pyrrole-2-carboxylate (3n)^3^**

Isolated as a yellow liquid, 28 mg, 72% yield; ^1^H NMR (400 MHz, CDCl_3_) δ 9.22 (s, 1H), 6.94 (td, *J* = 2.7, 1.6 Hz, 1H), 6.93 – 6.89 (m, 1H), 6.26 (dt, *J* = 3.6, 2.6 Hz, 1H), 5.00 – 4.92 (m, 1H), 1.98 – 1.87 (m, 2H), 1.84 – 1.72 (m, 2H), 1.62 – 1.47 (m, 3H), 1.47 – 1.36 (m, 2H), 1.36 – 1.27 (m, 1H); ^13^C NMR (100 MHz, CDCl_3_) δ 160.73, 123.46, 122.51, 114.93, 110.30, 72.56, 31.80, 25.45, 23.77; HRMS (ESI) calcd. for C_11_H_16_NO_2_: [M+H]^+^: 194.1181, found: 194.1184. CAS Number: 685563-26-2.

**2-methylallyl 1H-pyrrole-2-carboxylate (3o)**

Isolated as a yellow liquid, 23 mg, 71% yield; ^1^H NMR (400 MHz, CDCl_3_) δ 9.40 (s, 1H), 6.97 (t, *J* = 2.5 Hz, 2H), 6.27 (dd, *J* = 6.1, 2.6 Hz, 1H), 5.05 (s, 1H), 4.96 (s, 1H), 4.70 (s, 2H), 1.81 (s, 3H); ^13^C NMR (100 MHz, CDCl_3_) δ 160.97, 140.13, 123.12, 122.61, 115.46, 112.82, 110.47, 67.41, 19.52; HRMS (ESI) calcd. for C_9_H_12_NO_2_: [M+H]^+^: 166.0868, found: 166.0863. CAS Number: 1933575-18-8.

**benzyl 1H-indole-2-carboxylate (3p)^7^**

Isolated as a yellow liquid, 41 mg, 81% yield; ^1^H NMR (400 MHz, CDCl_3_) δ 8.95 (s, 1H), 7.68 (d, *J* = 8.1 Hz, 1H), 7.46 (d, *J* = 6.7 Hz, 2H), 7.40 (dd, *J* = 7.9, 6.0 Hz, 3H), 7.36 (dd, *J* = 9.5, 2.6 Hz, 1H), 7.34 – 7.29 (m, 1H), 7.28 (d, *J* = 1.1 Hz, 1H), 7.15 (t, *J* = 7.5 Hz, 1H), 5.39 (s, 2H); ^13^C NMR (100 MHz, CDCl_3_) δ 171.22, 161.80, 136.94, 135.76, 128.67, 128.42, 128.29, 127.45, 127.08, 125.51, 122.63, 120.86, 111.90, 109.19, 66.66, 60.42, 31.59, 22.66, 21.05, 18.38, 14.19, 14.12; HRMS (ESI) calcd. for C_16_H_14_NO_2_: [M+H]^+^: 252.1025, found: 252.1024. CAS Number: 78277-27-7.

# 5.Reference

1.Feng YZ, Cai Y, Fu X, Zheng L, Xiao ZB, Zhao MM. Comparison of aroma-active compounds in broiler broth and native chicken broth by aroma extract dilution analysis (AEDA), odor activity value (OAV) and omission experiment. Food Chem. 2018;265:274–280. <https://doi.org/10.1016/j.foodchem.2018.05.043>.

2.Schnegotzki, Romina; Wiebach, Vincent; Sanchez-Hidalgo, Marina; Tietzmann, Marcel; zur Bonsen, Andreas B.; Genilloud, Olga; Suessmuth, Roderich D. Total synthesis and biosynthesis of cyclodepsipeptide cochinmicin I. Org. Lett. 2022, 24, 12, 2344–2348.

<https://doi.org/10.1021/acs.orglett.2c00525>

3.Silvestri, Romano; Artico, Marino; La Regina, Giuseppe; De Martino, Gabriella; La Colla, Massimiliano; Loddo, Roberta; La Colla, Paolo. Anti-HIV-1 activity of pyrryl aryl sulfone (PAS) derivatives: synthesis and SAR studies of novel esters and amides at the position 2 of the pyrrole nucleus. IL Farmaco. (2004), 59, (3), 201-210.

<https://doi.org/10.1016/j.farmac.2003.11.004>

4.Raja, S.; Xavier, N.; Arulraj, S. J. Action of boron trifluoride etherate and stannic chloride on heterocyclic aromatic acetals. Indian J. Chem. B. (1989), 28B, (8), 687-689.

5.Wischang, Diana; Hartung, Jens. Parameters for bromination of pyrroles in bromoperoxidase-catalyzed oxidations. Tetrahedron (2011), 67, (22), 4048-4054.

<https://doi.org/10.1016/j.tet.2011.04.010>

6.Troegel, Benjamin; Lindel, Thomas. Microwave-Assisted Fluorination of 2-Acylpyrroles: Synthesis of Fluorohymenidin. Org. Lett. (2012), 14, (2), 468-471.

<https://doi.org/10.1021/ol2029993>

7. Hatano, Manabu; Tabata, Yuji; Yoshida, Yurika; Toh, Kohei; Yamashita, Kenji; Ogura, Yoshihiro; Ishihara, Kazuaki. Metal-free transesterification catalyzed by tetramethylammonium methyl carbonate. Green Chem. (2018), 20, (6), 1193-1198.

<https://doi.org/10.1039/C7GC03858E>

# 6. Copy of ^1^H and ^13^C NMR spectra


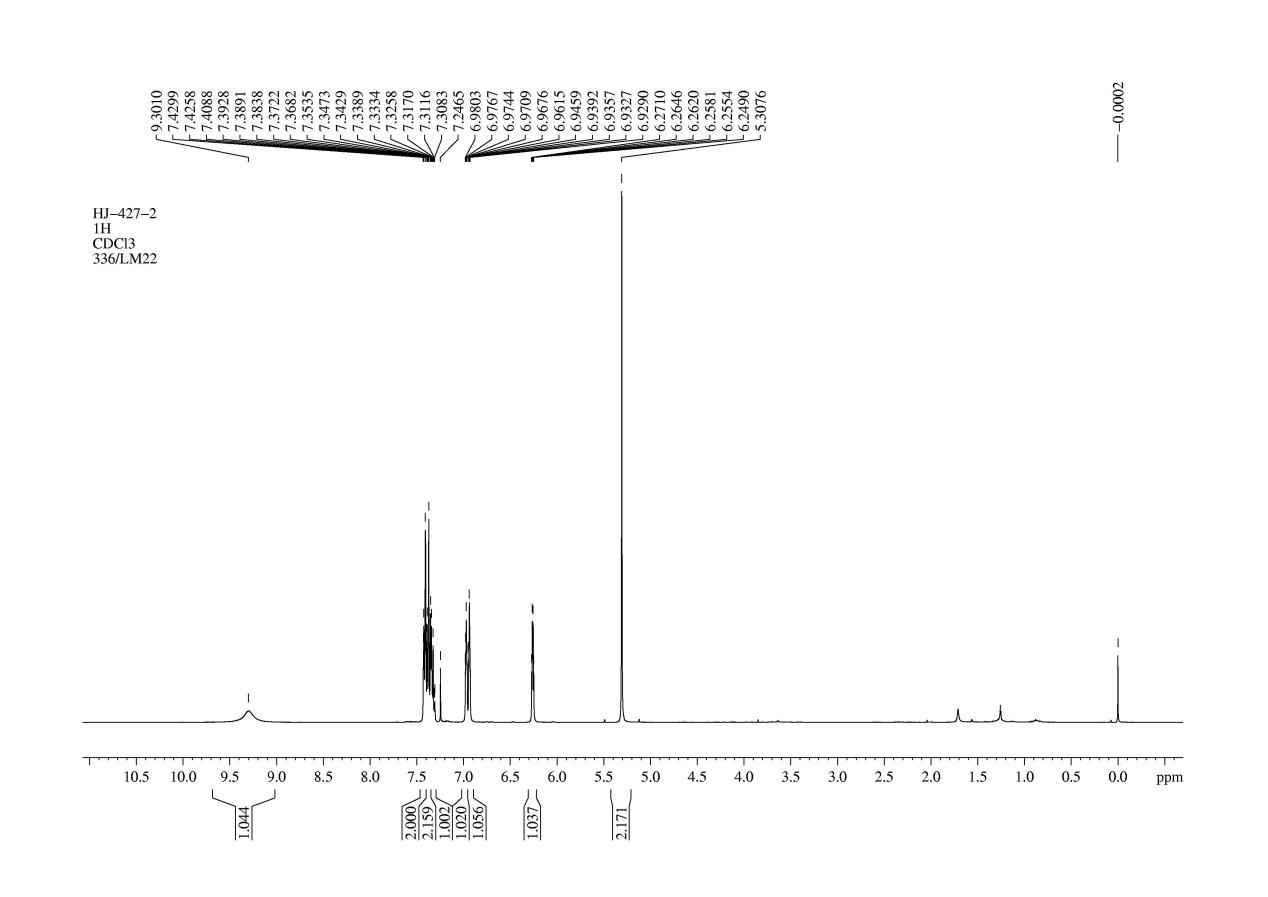


**Figure S1:** ^1^H NMR spectrum of compound **3a**

**
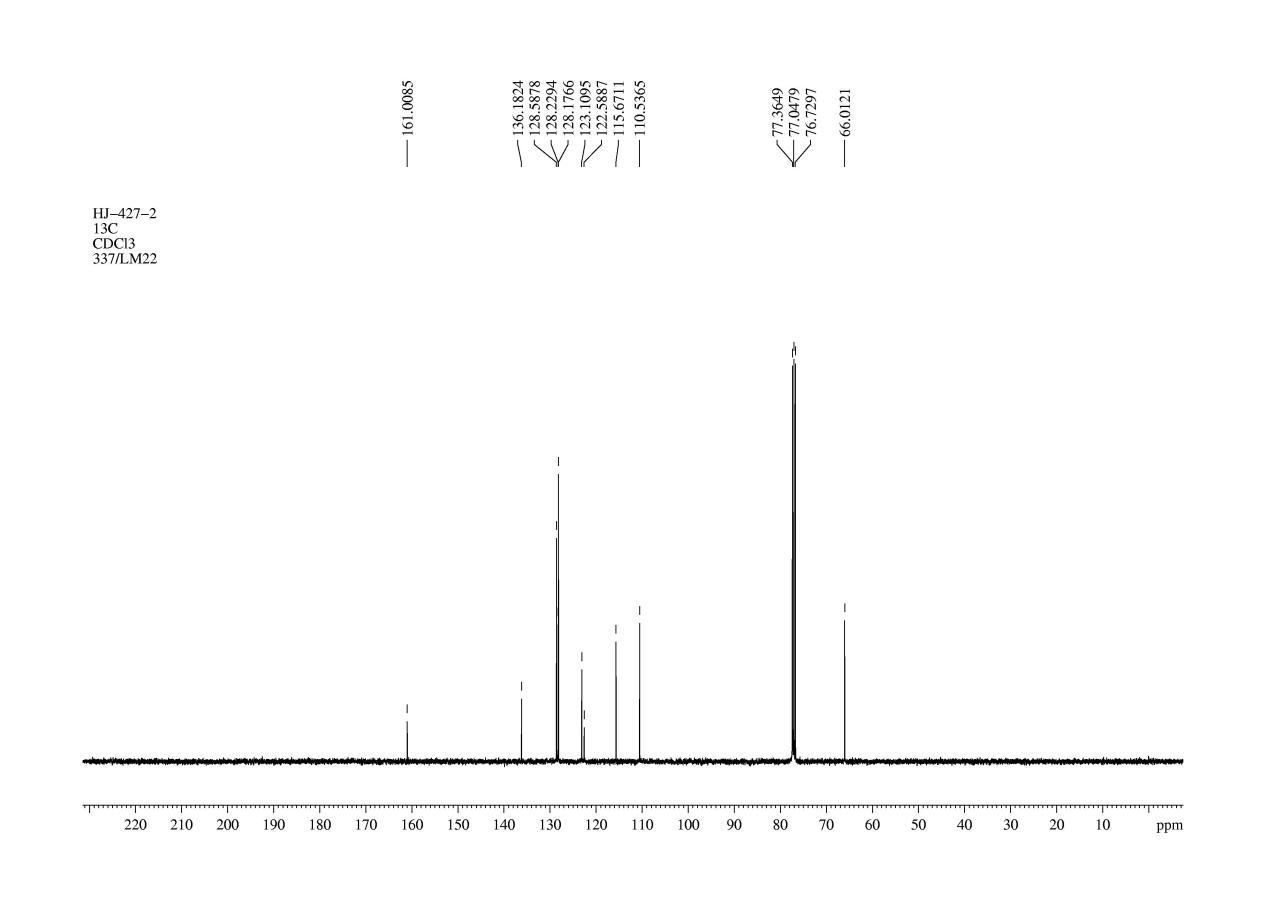
**

**Figure S2:** ^13^C NMR spectrum of compound **3a**


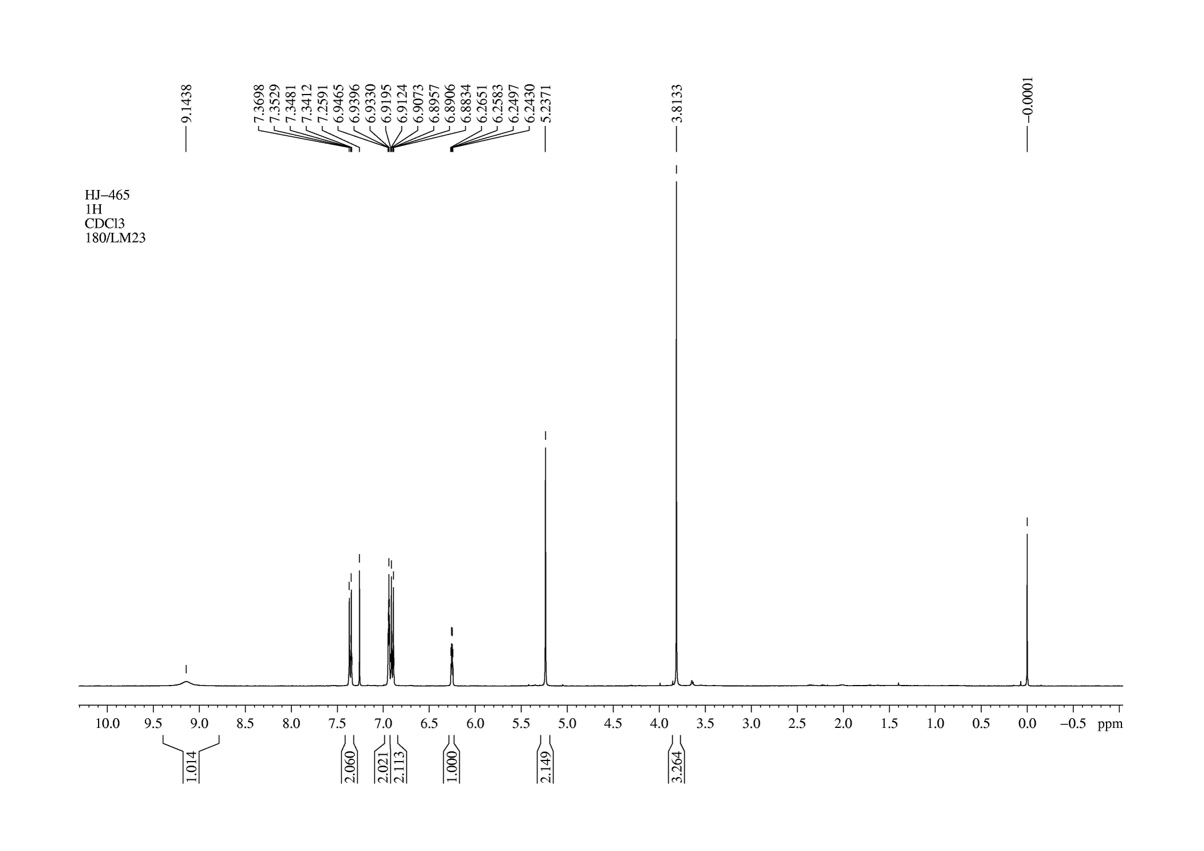


**Figure S5:** ^1^H NMR spectrum of compound **3b**


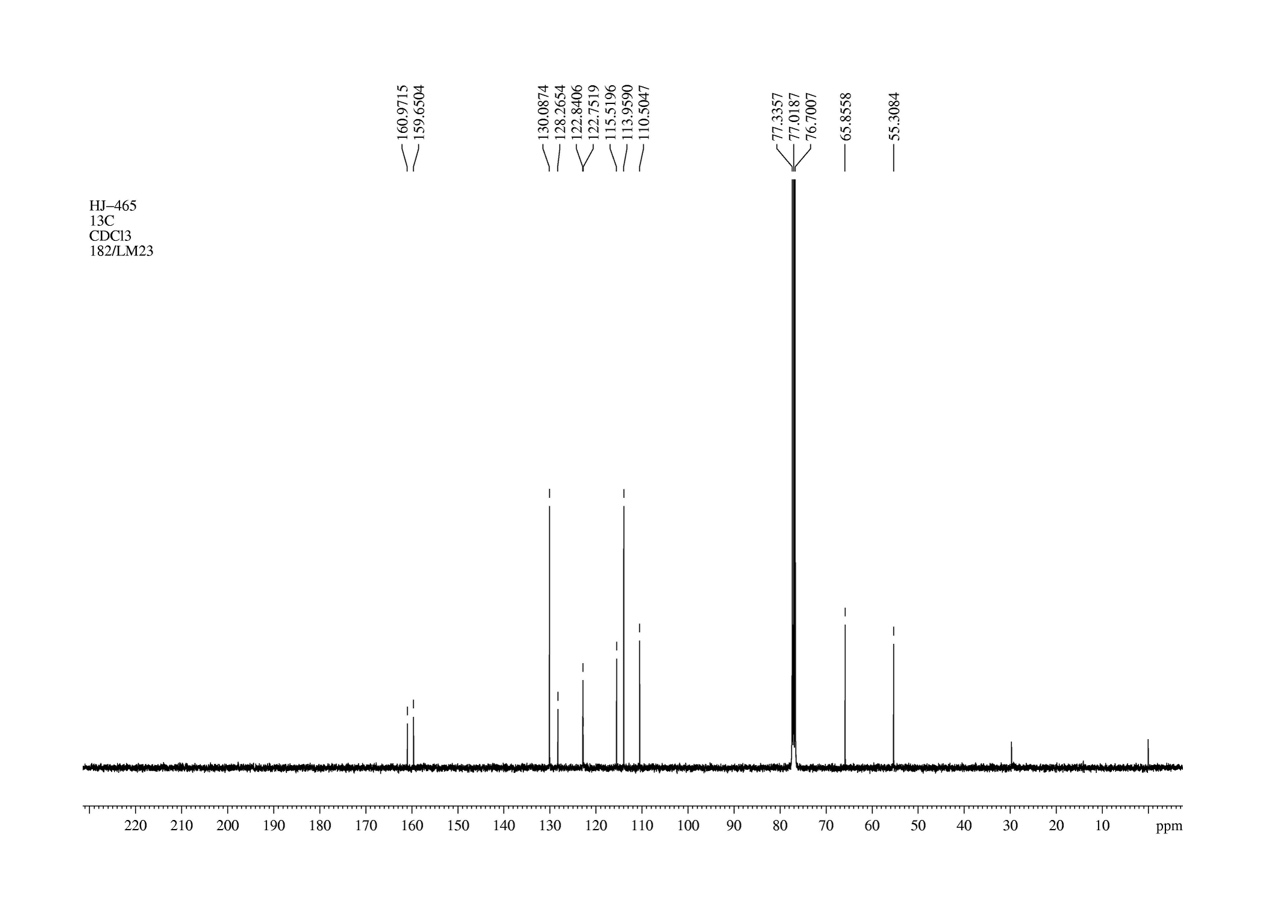


**Figure S6:** ^13^C NMR spectrum of compound **3b**


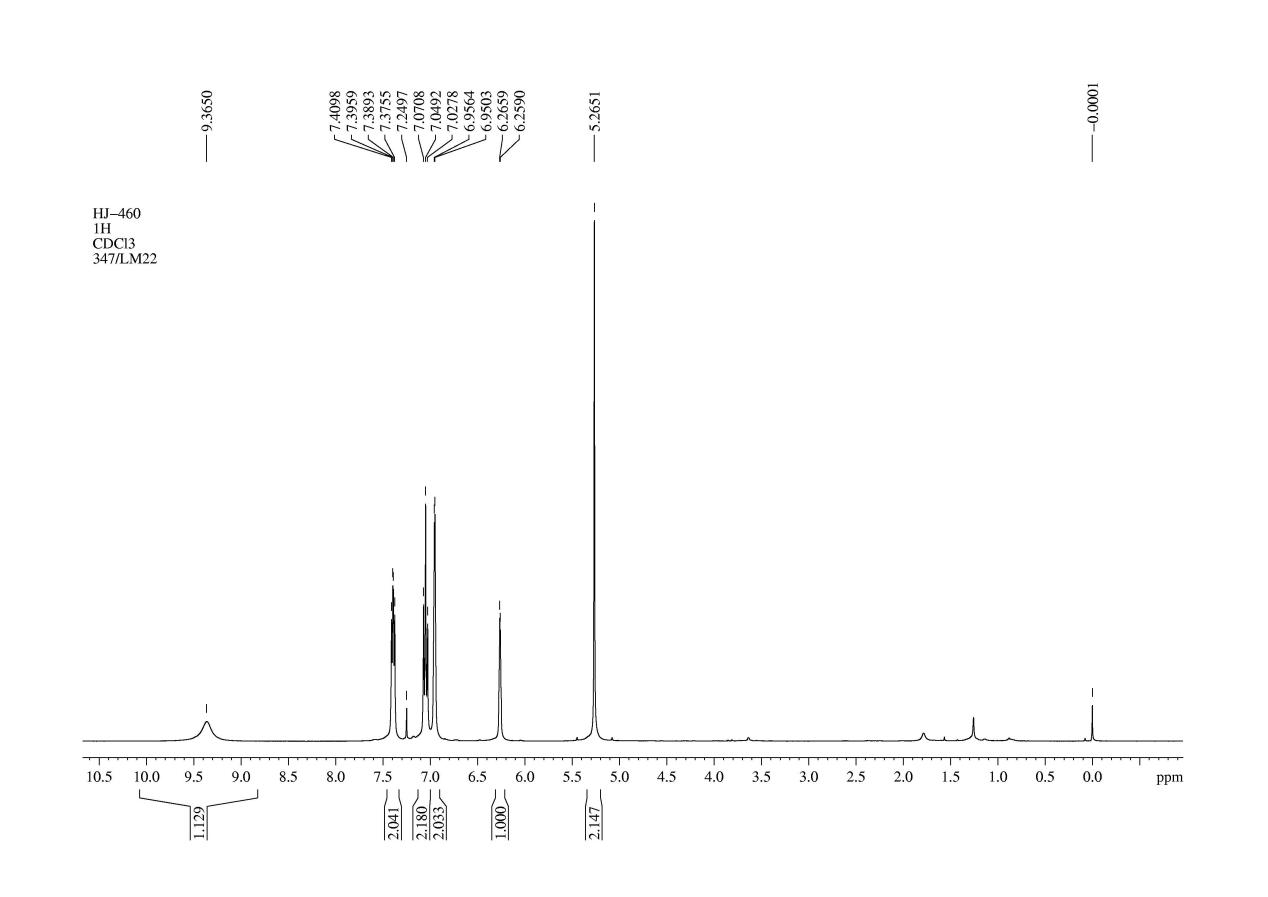


**Figure S3:** ^1^H NMR spectrum of compound **3c**

**
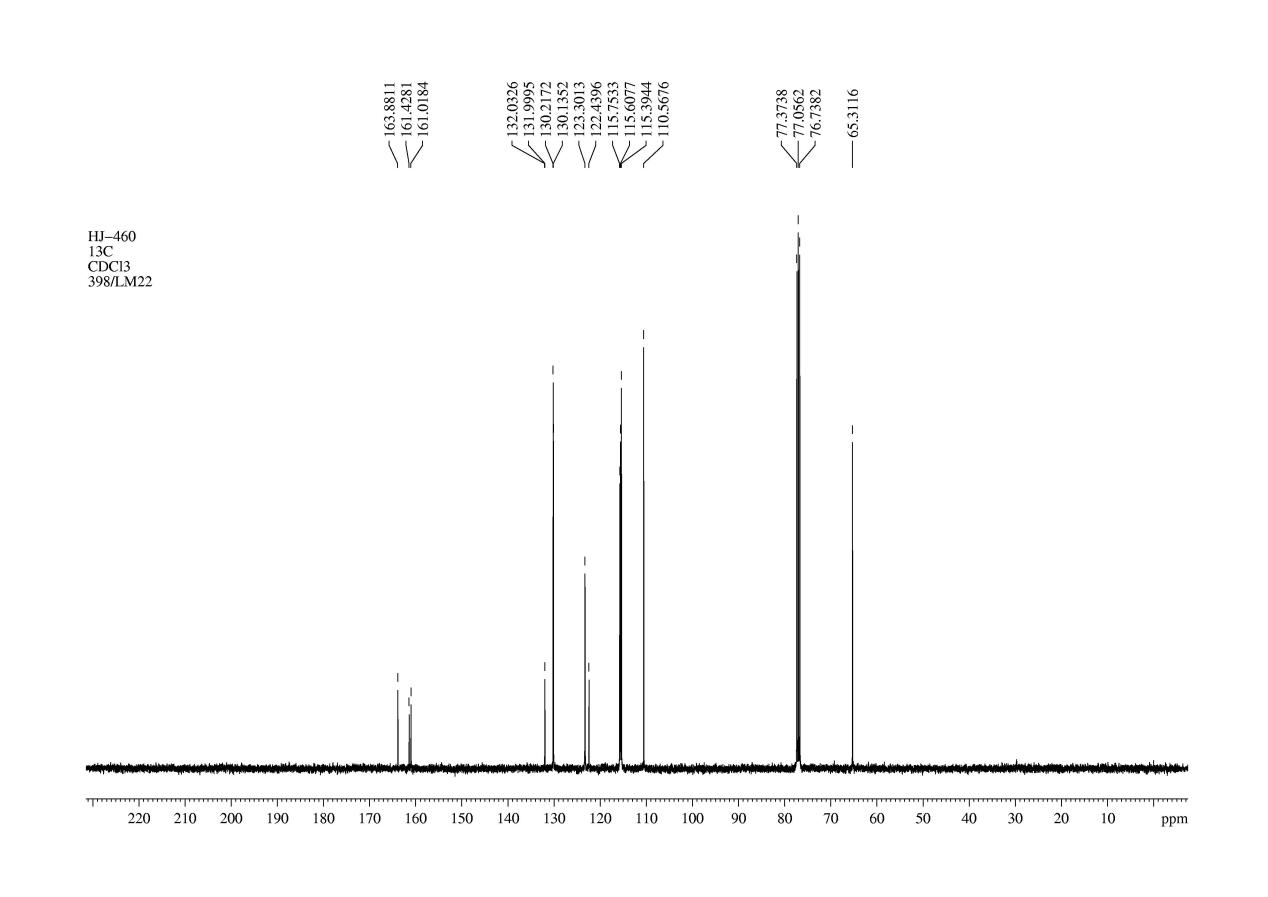
**

**Figure S4:** ^13^C NMR spectrum of compound **3c**


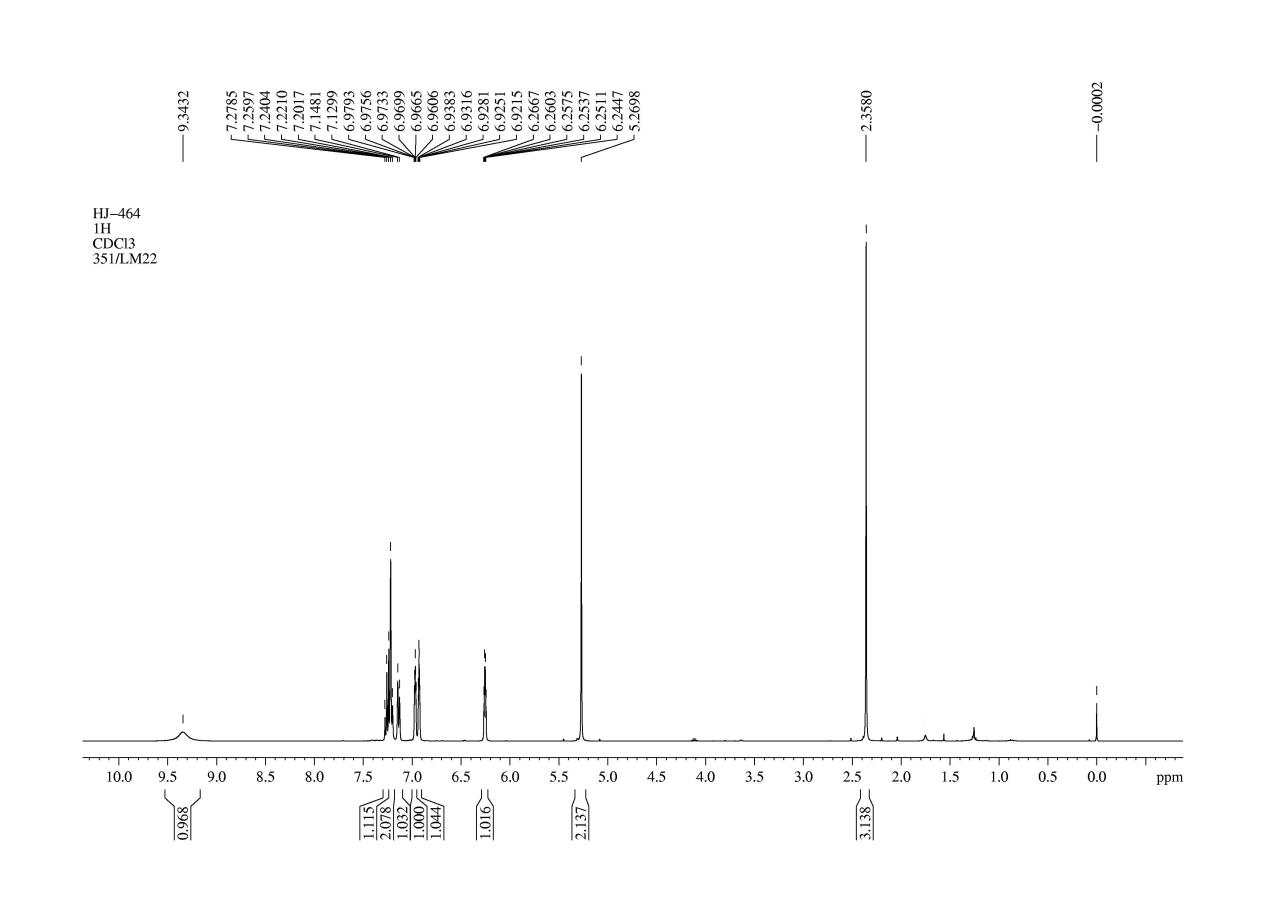


**Figure S7:** ^1^H NMR spectrum of compound **3e**

**
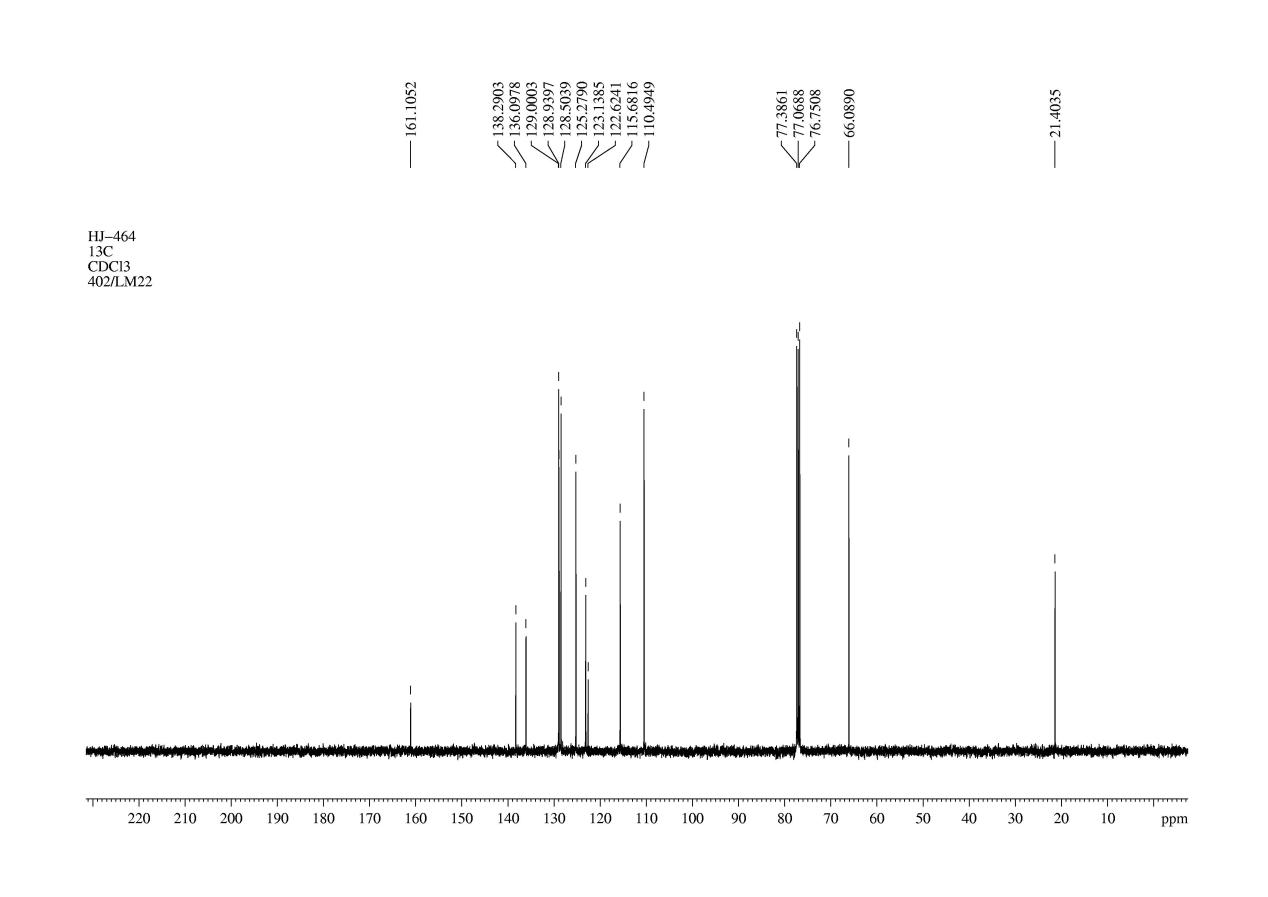
**

**Figure S8:** ^13^C NMR spectrum of compound **3e**


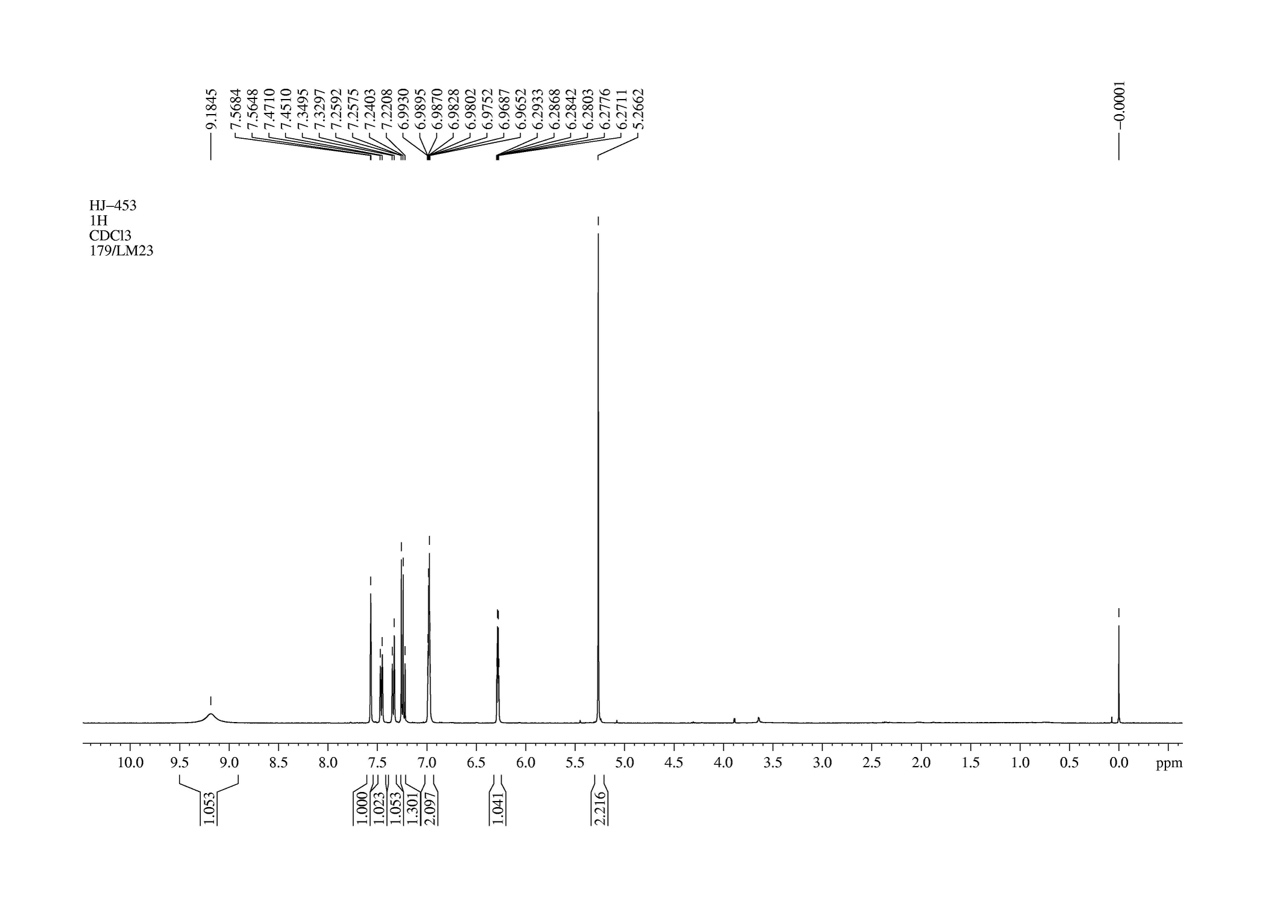


**Figure S9:** ^1^H NMR spectrum of compound **3f**

**
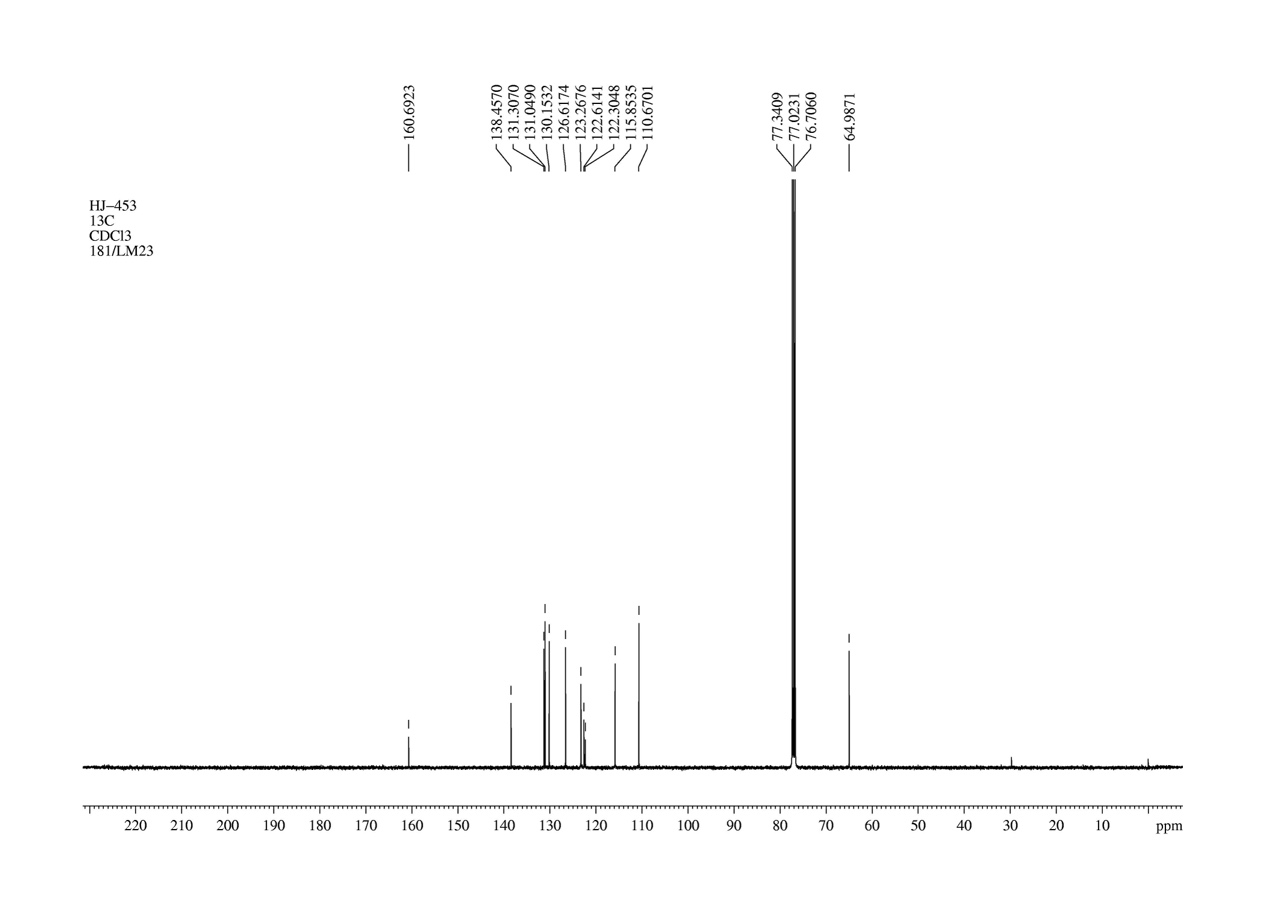
**

**Figure S10:** ^13^C NMR spectrum of compound **3f**


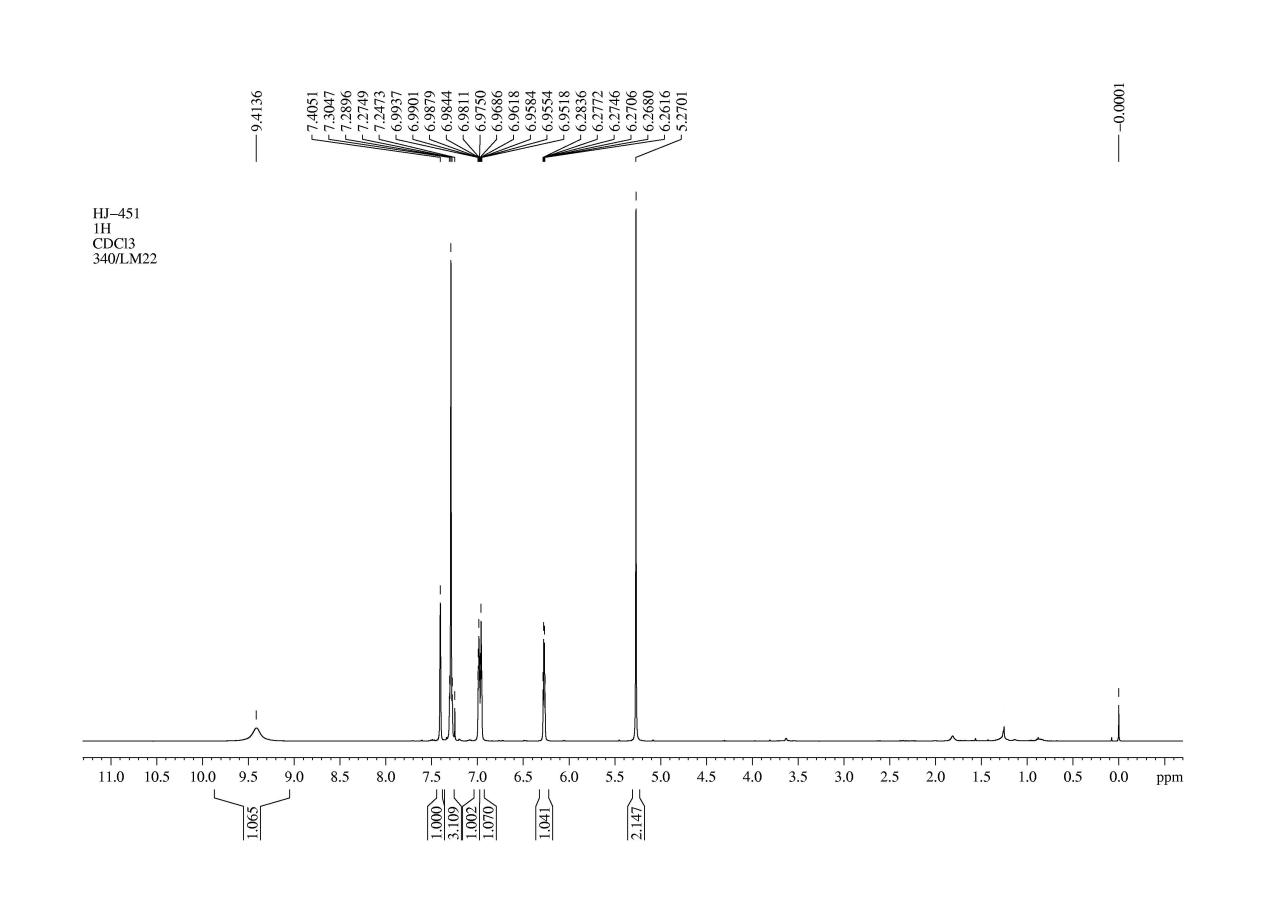


**Figure S11:** ^1^H NMR spectrum of compound **3g**

**
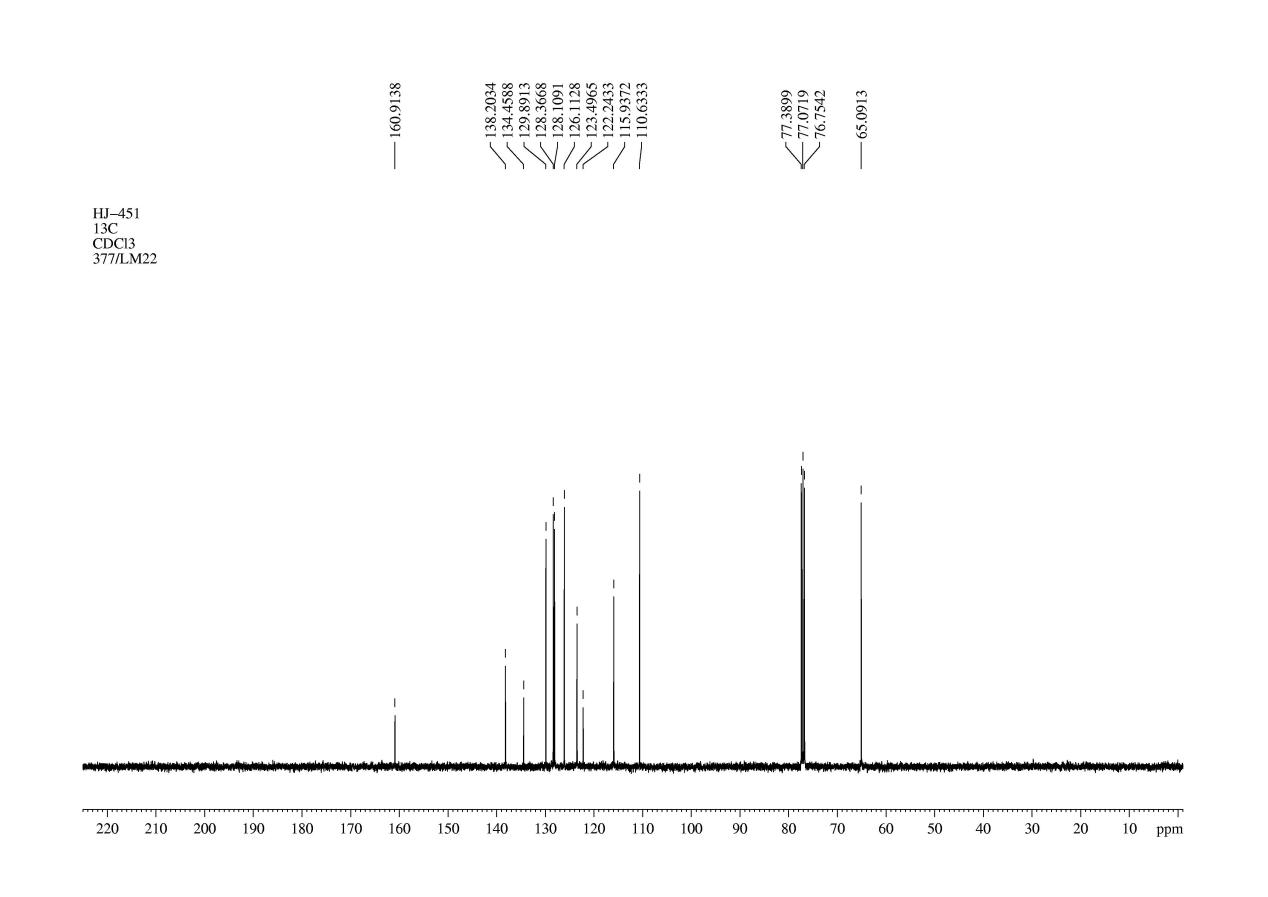
**

**Figure S12:** ^13^C NMR spectrum of compound **3g**


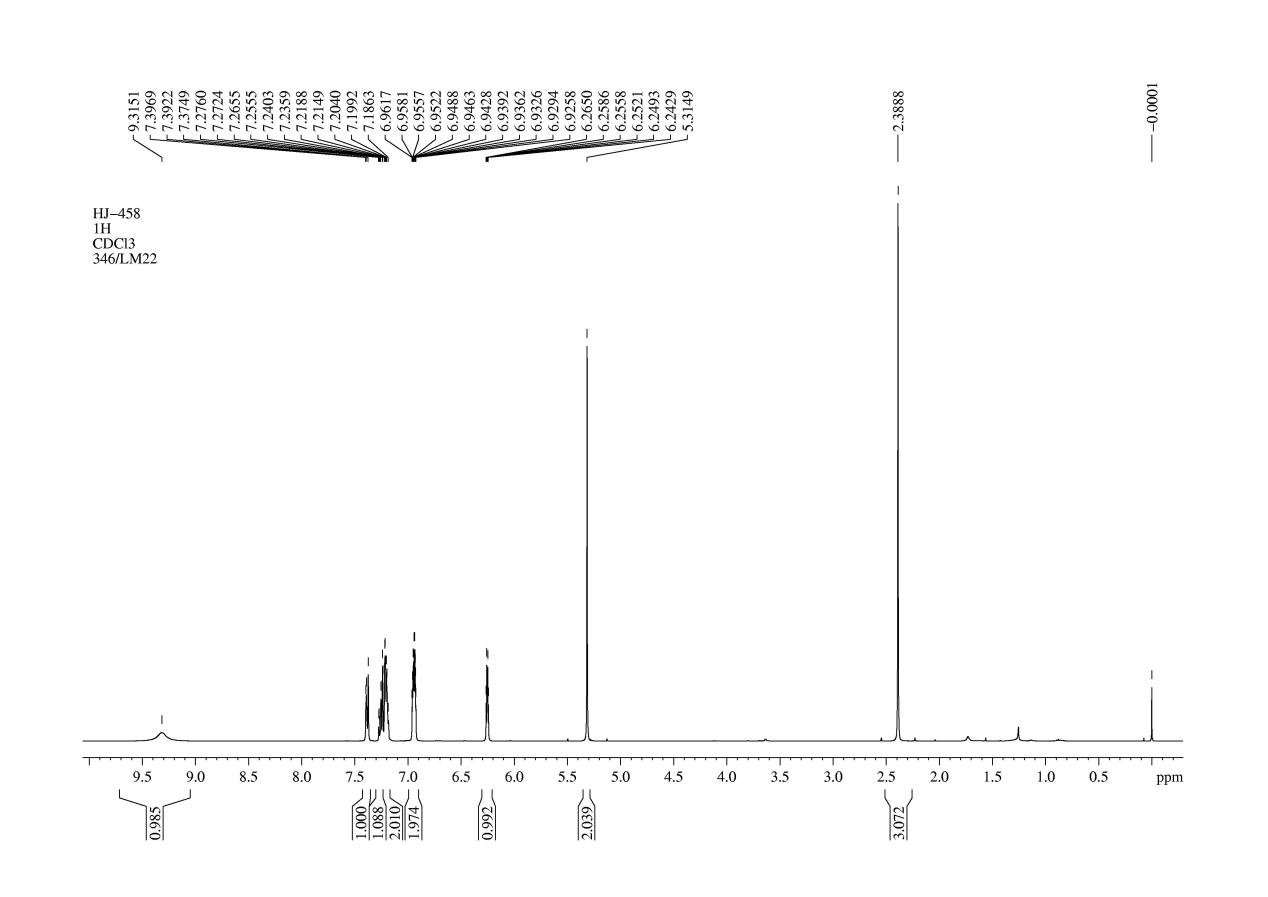


**Figure S13:** ^1^H NMR spectrum of compound **3h**

**
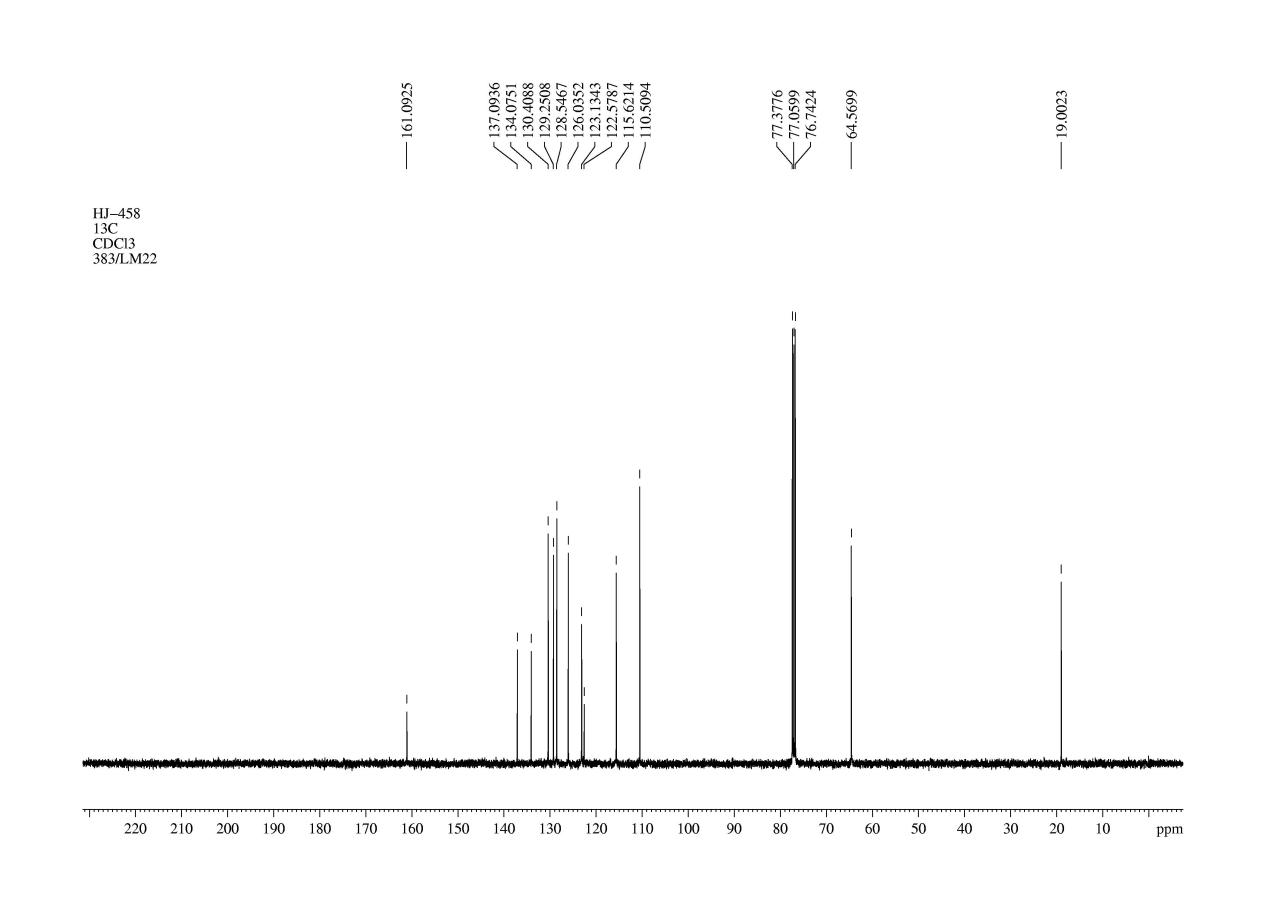
**

**Figure S14:** ^13^C NMR spectrum of compound **3h**

1
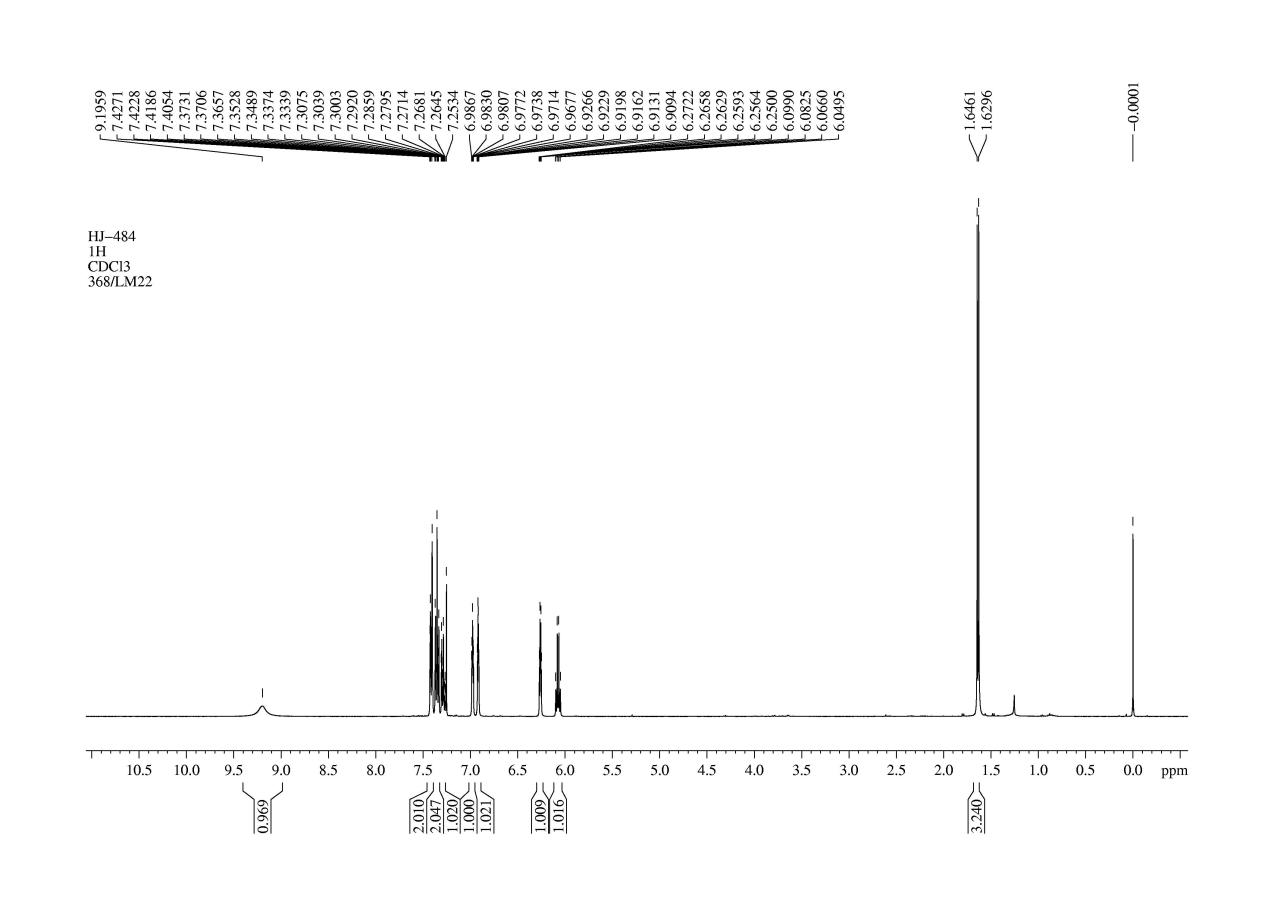
 **Figure S15:** ^1^H NMR spectrum of compound **3i**

**
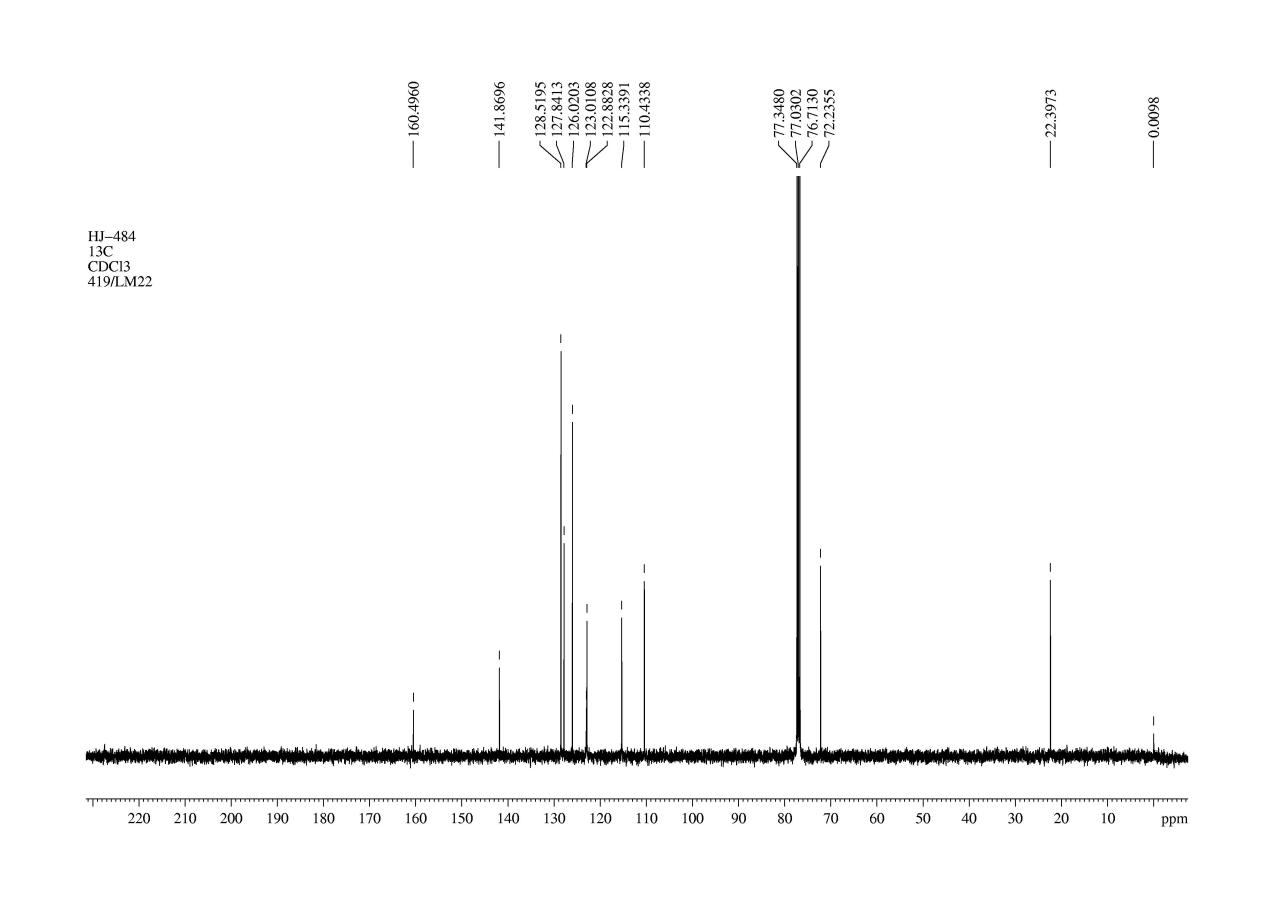
**

**Figure S16:** ^13^C NMR spectrum of compound **3i**


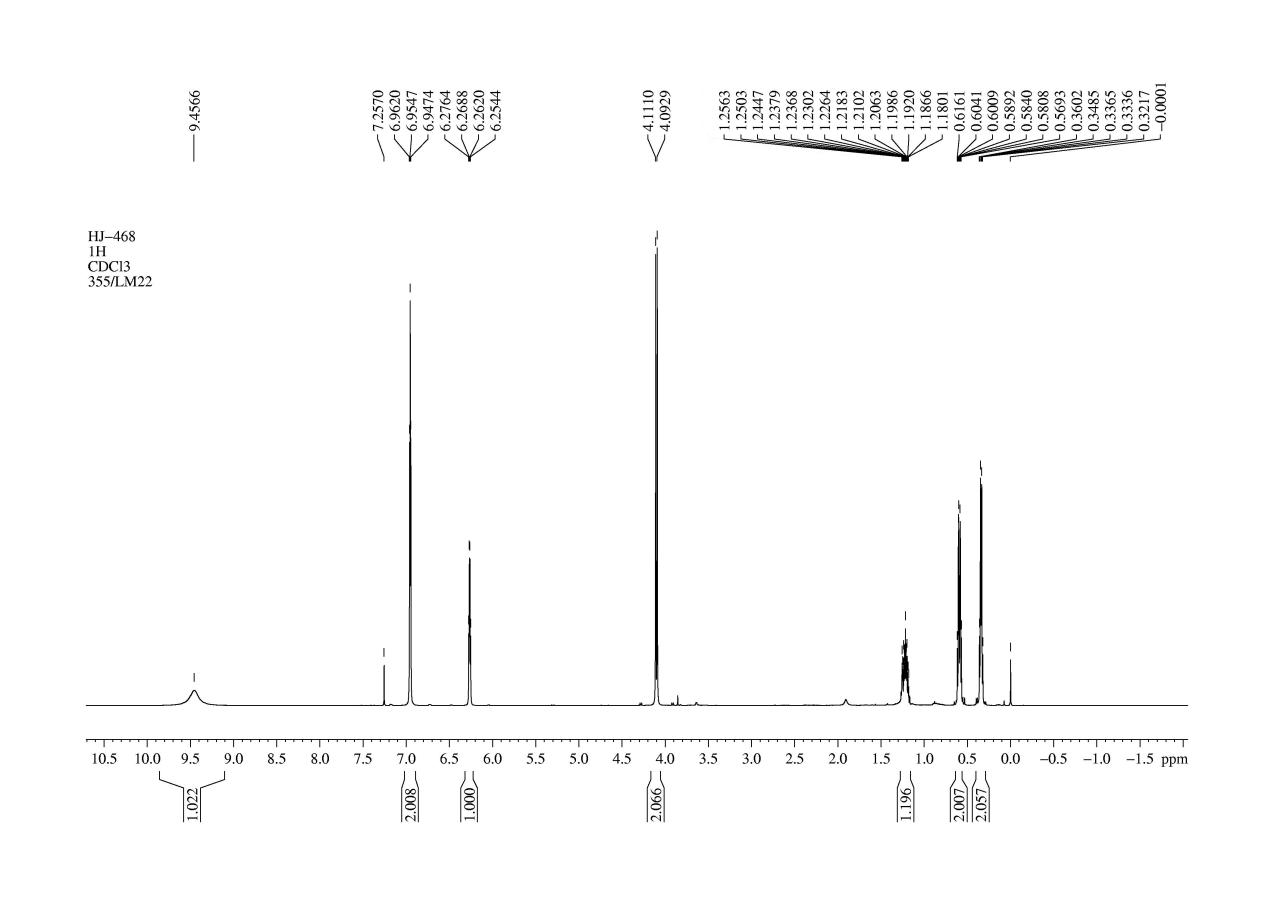


**Figure S17:** ^1^H NMR spectrum of compound **3j**

**
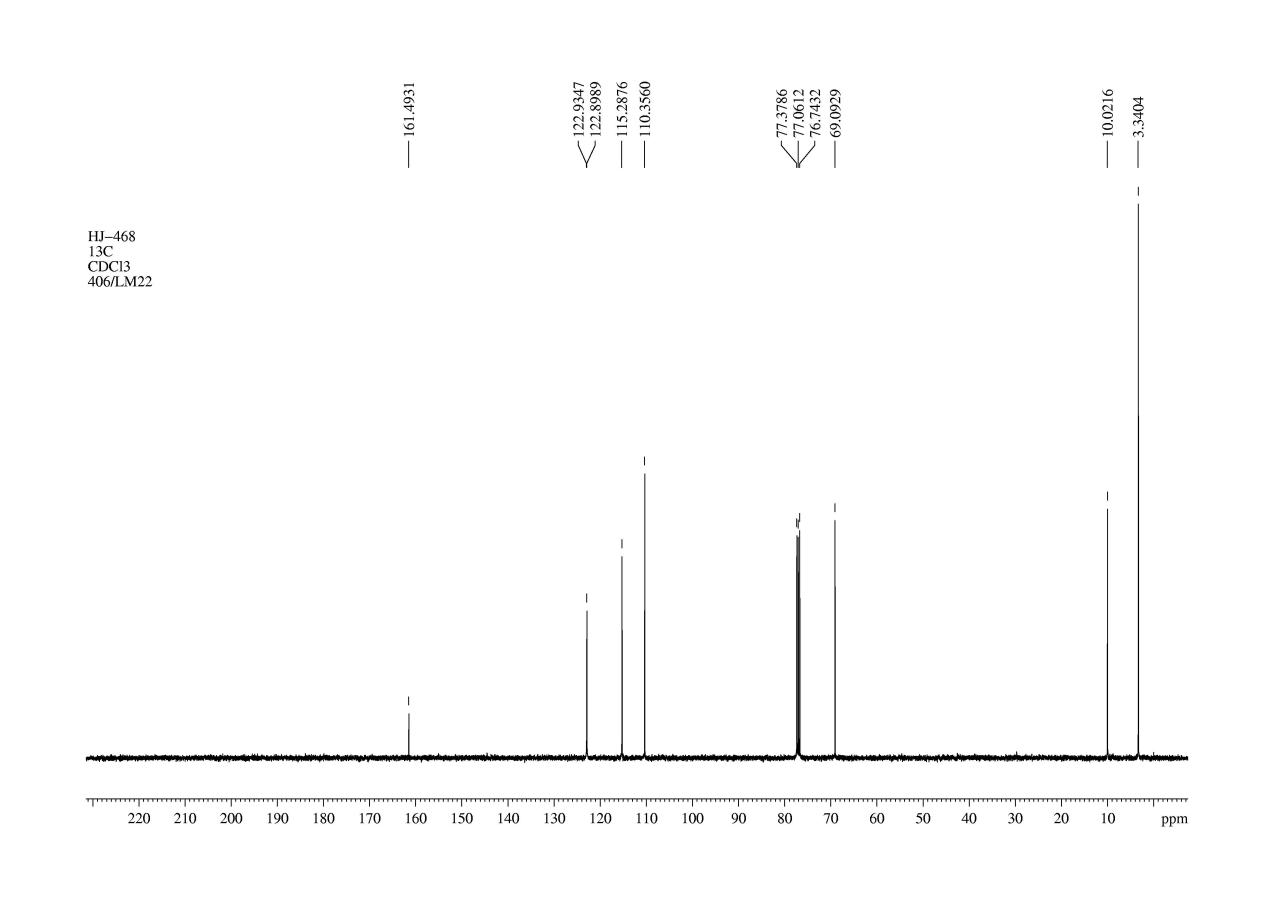
**

**Figure S18:** ^13^C NMR spectrum of compound **3j**


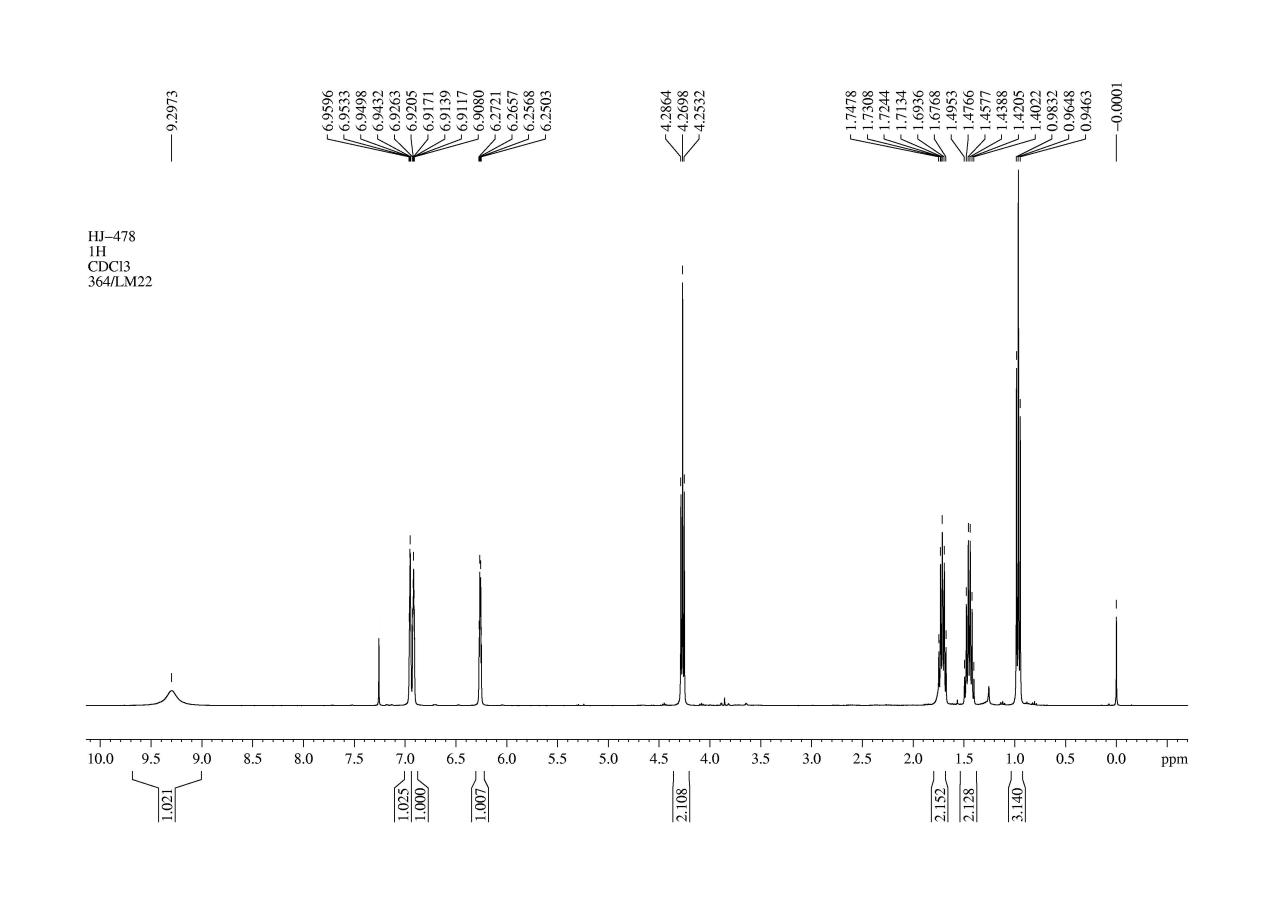


**Figure S19:** ^1^H NMR spectrum of compound **3k**

**
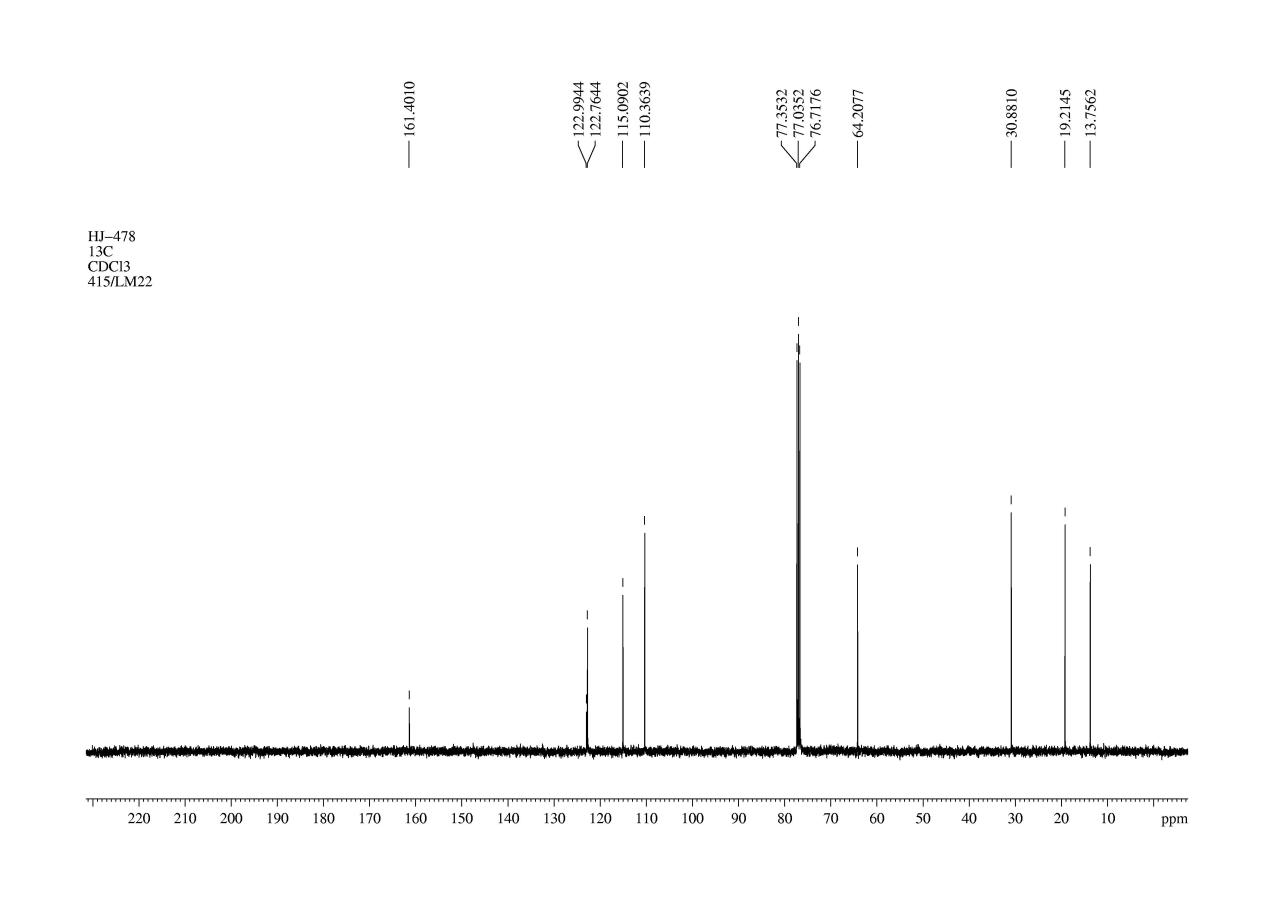
**

**Figure S20:** ^13^C NMR spectrum of compound **3k**


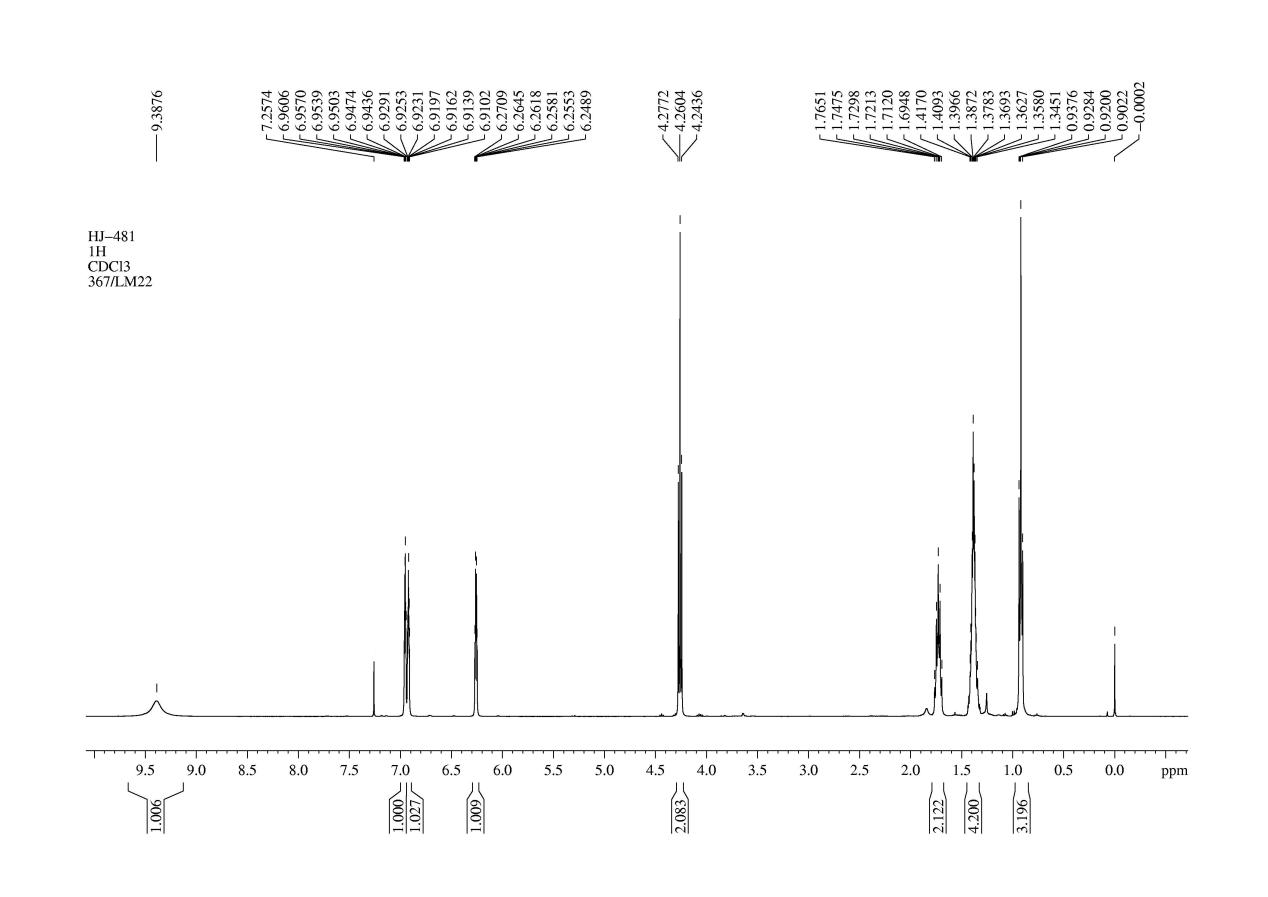


**Figure S21:** ^1^H NMR spectrum of compound **3l**

**
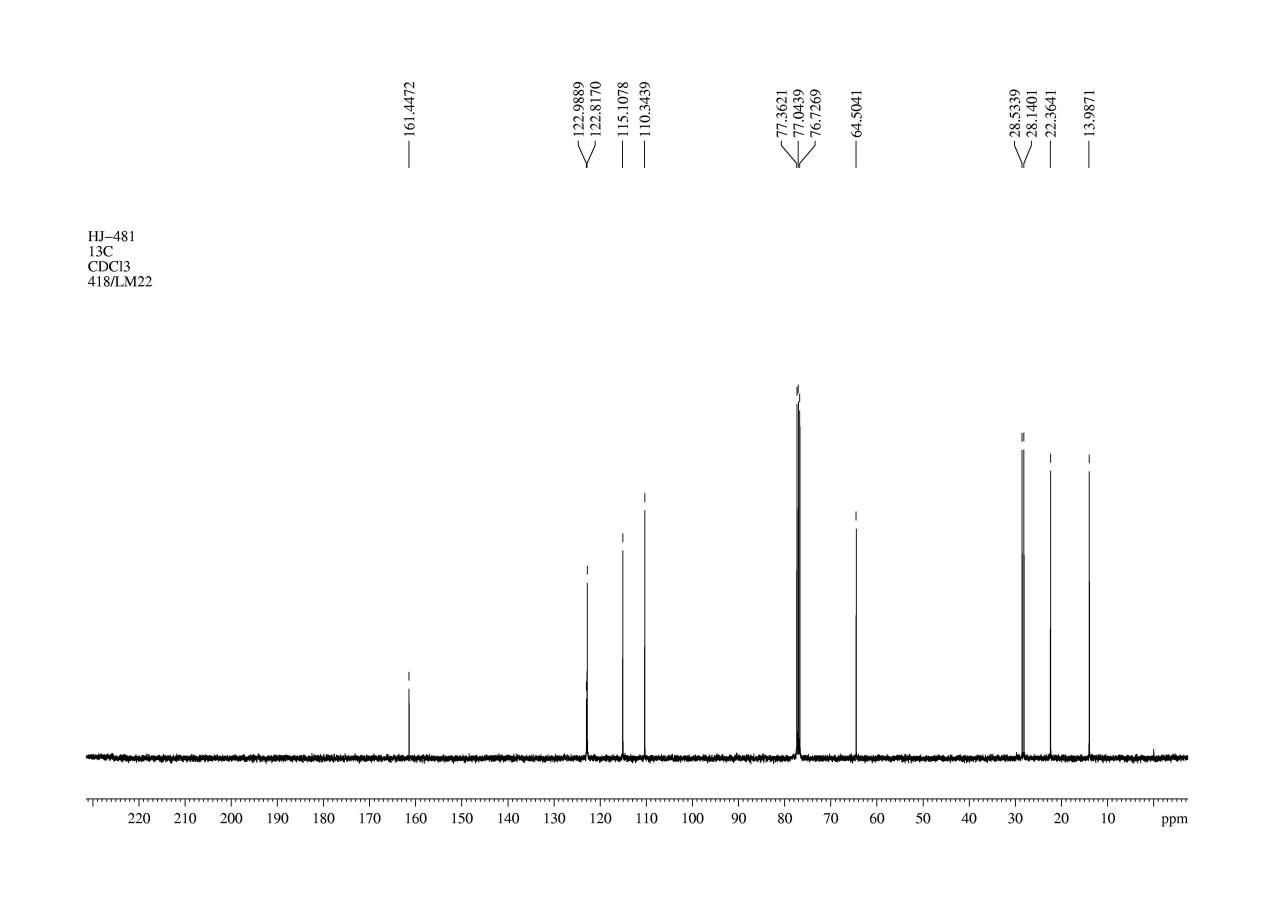
**

**Figure S22:** ^13^C NMR spectrum of compound **3l**


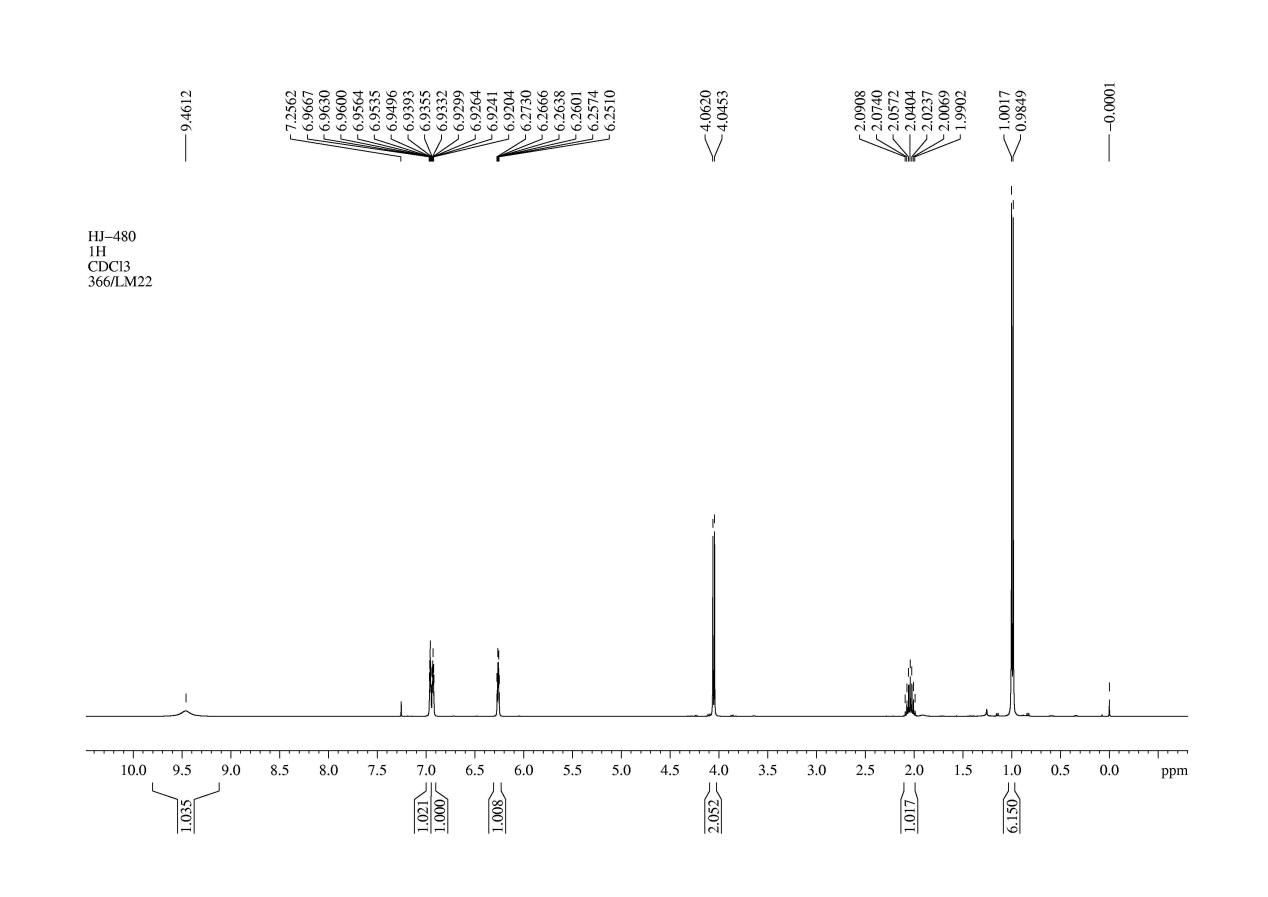


**Figure S23:** ^1^H NMR spectrum of compound **3m**

**
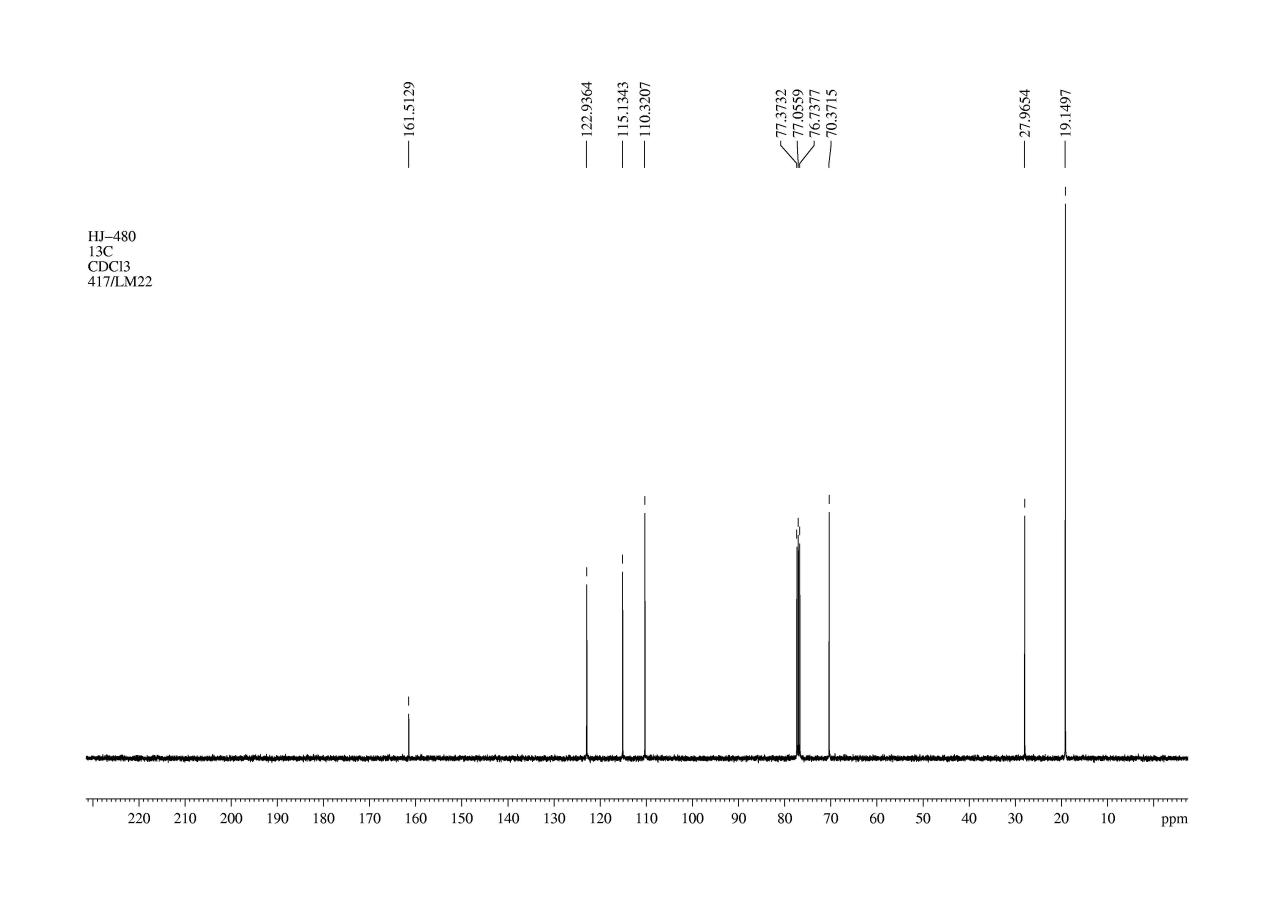
**

**Figure S24:** ^13^C NMR spectrum of compound **3m**


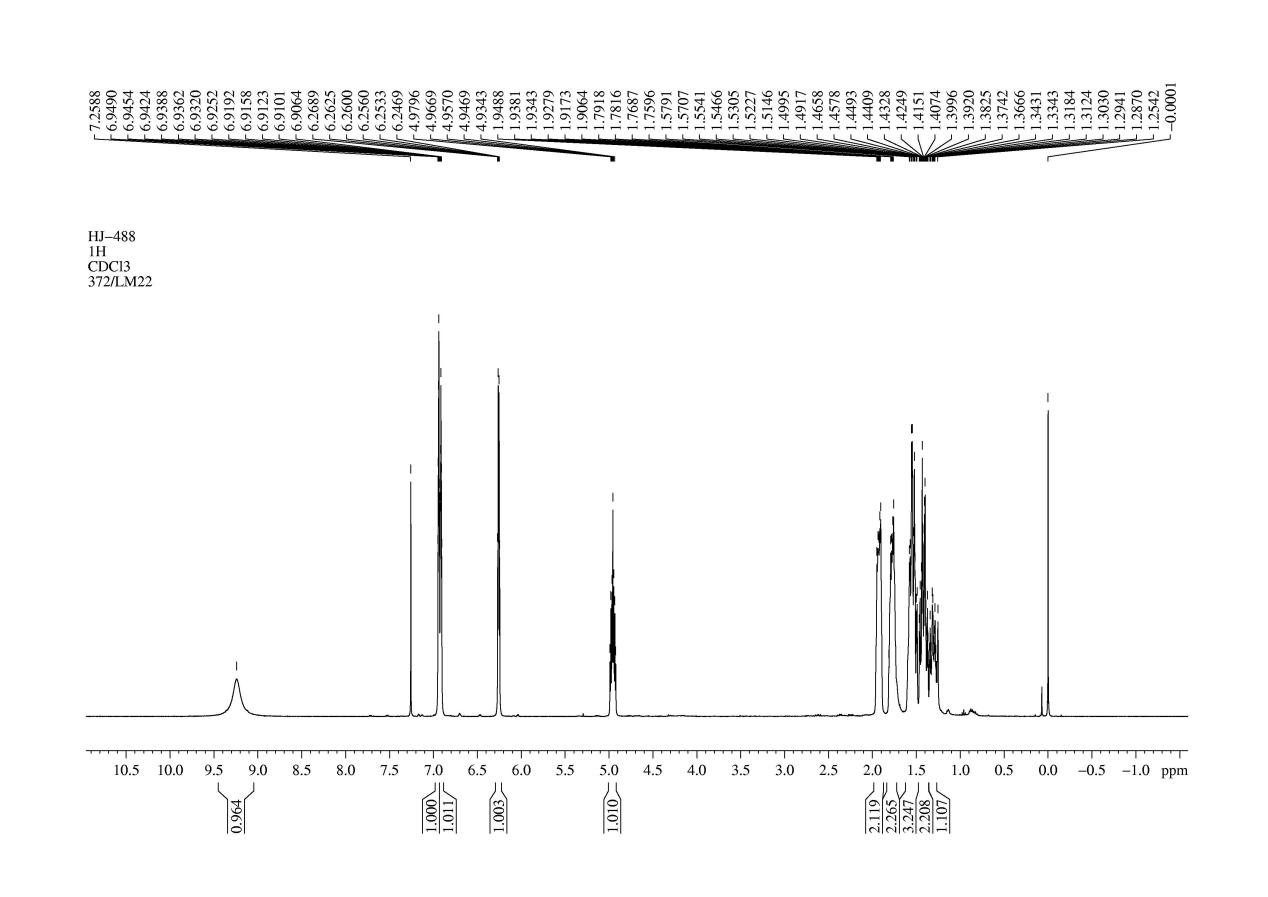


**Figure S25:** ^1^H NMR spectrum of compound **3n**

**
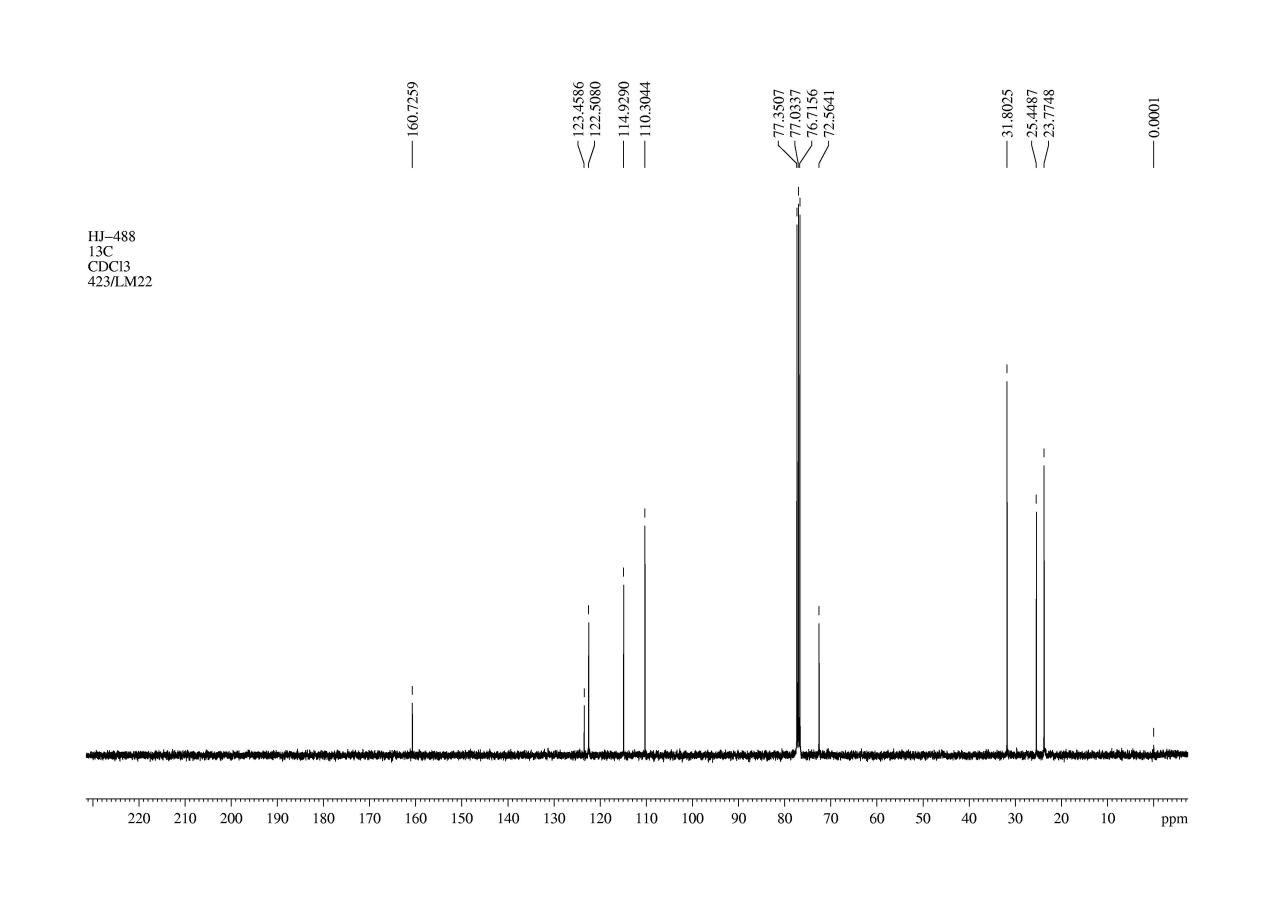
**

**Figure S26:** ^13^C NMR spectrum of compound **3n**


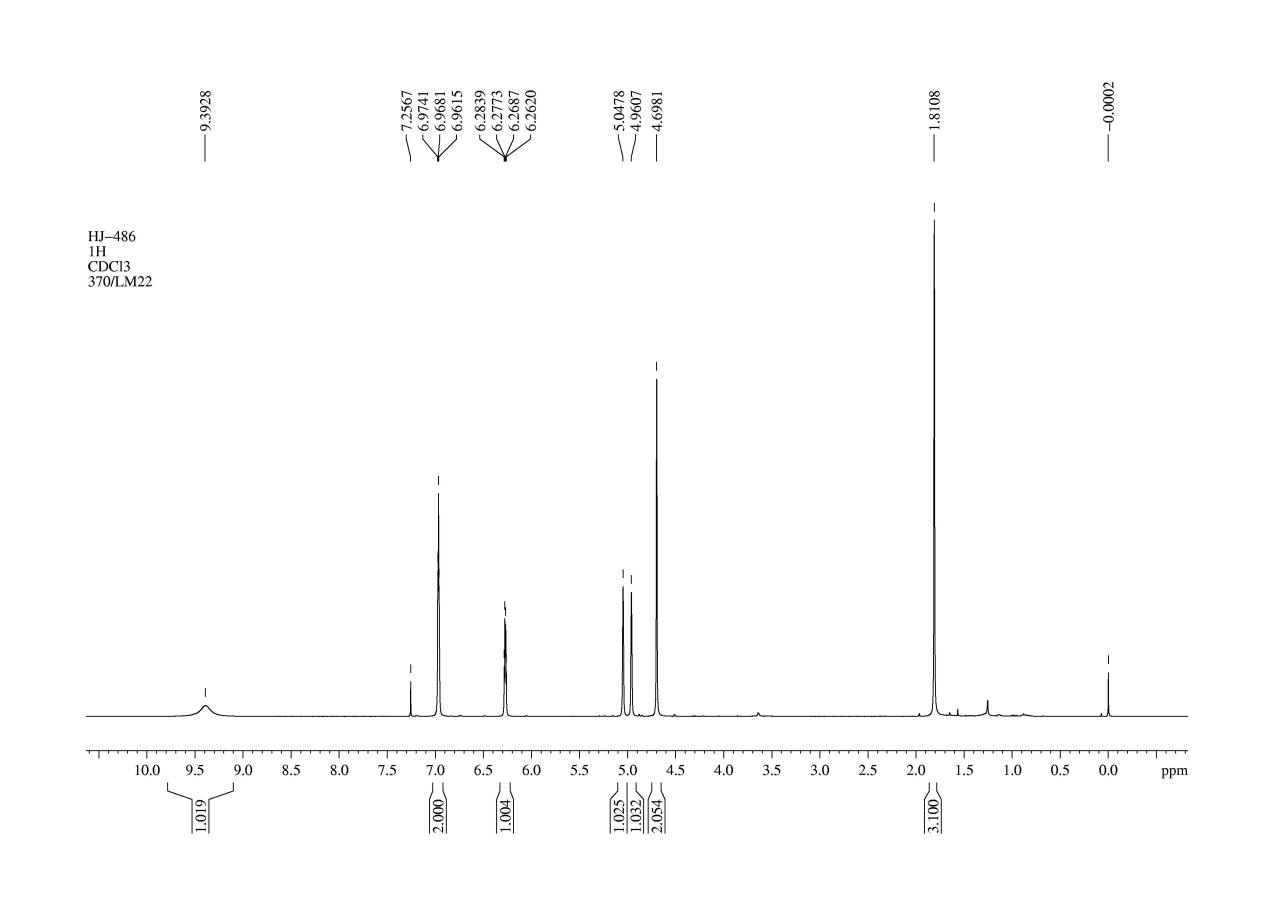


**Figure S27:** ^1^H NMR spectrum of compound **3o**

**
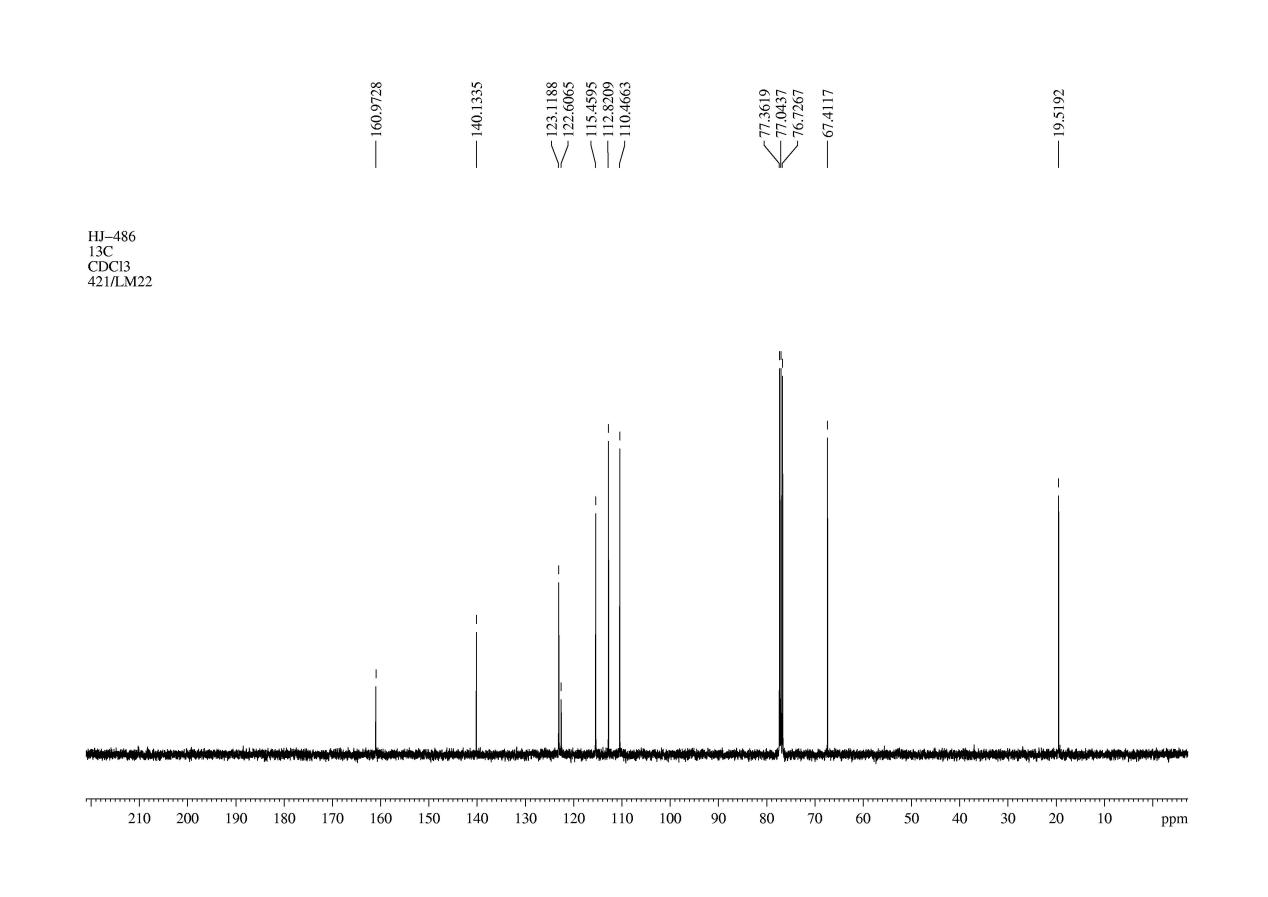
**

**Figure S28:** ^13^C NMR spectrum of compound **3o**


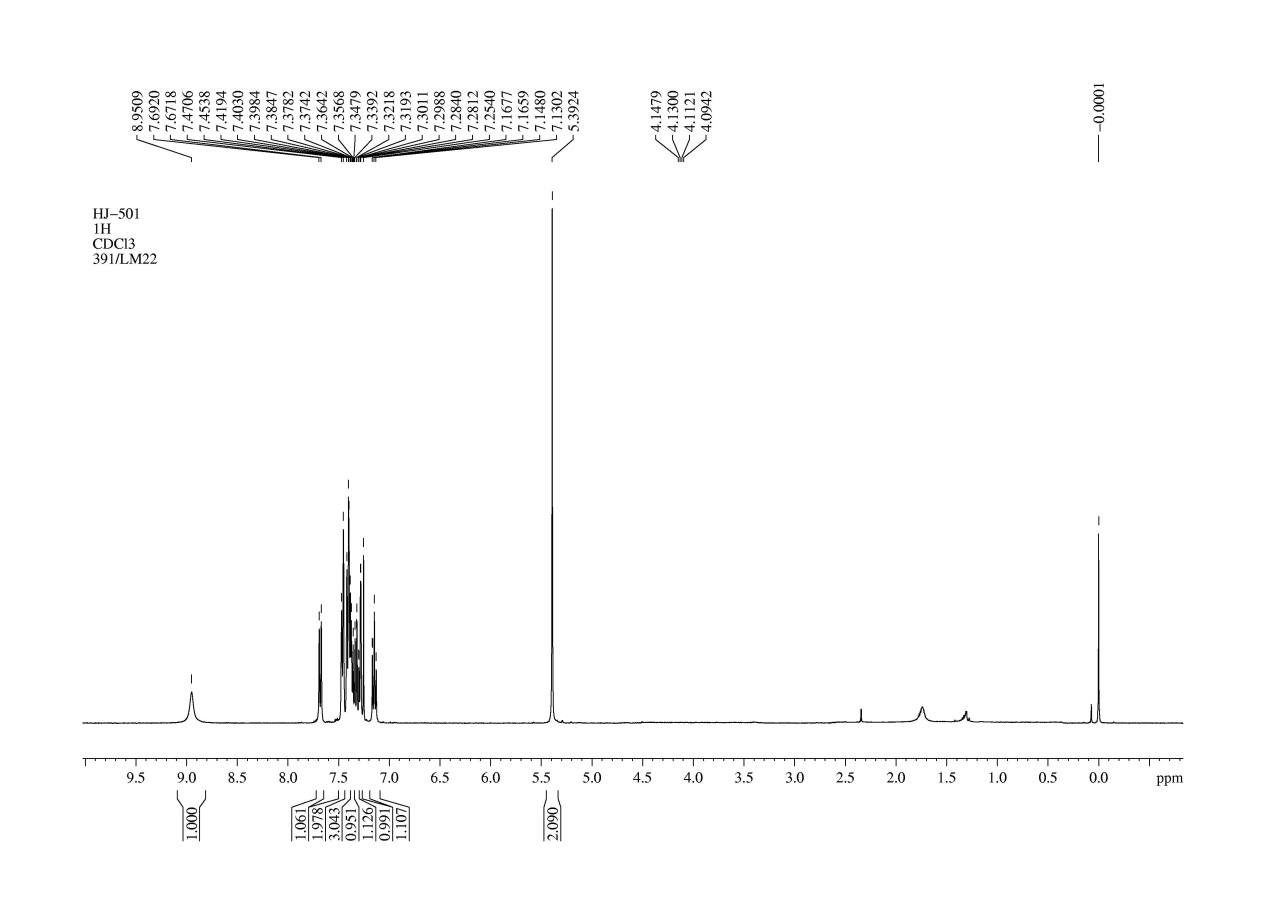


**Figure S29:** ^1^H NMR spectrum of compound **3p**

**
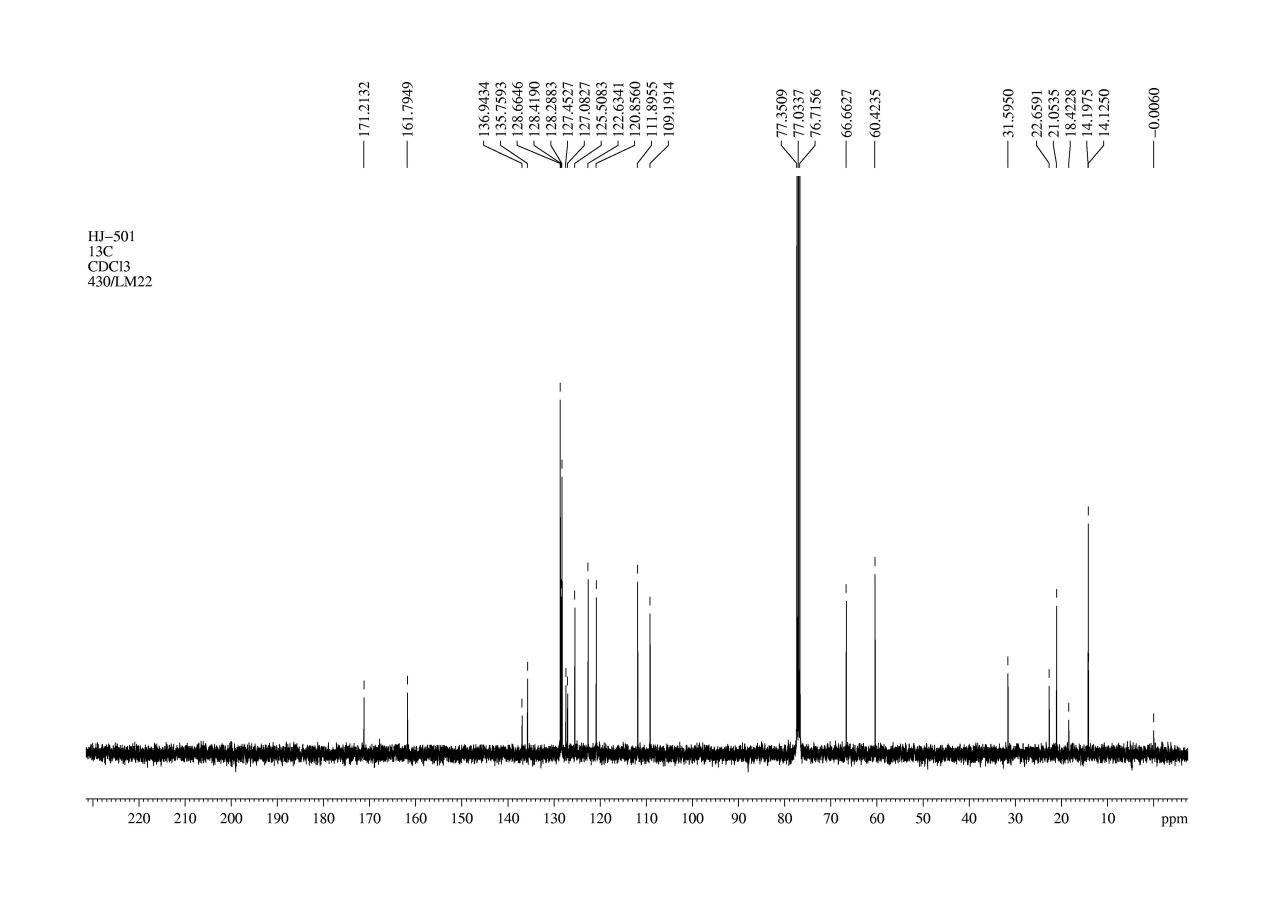
**

**Figure S30:** ^13^C NMR spectrum of compound **3p**

# 7. Copy of Gas chromatography-mass spectrometry ion flow chromatogram

**
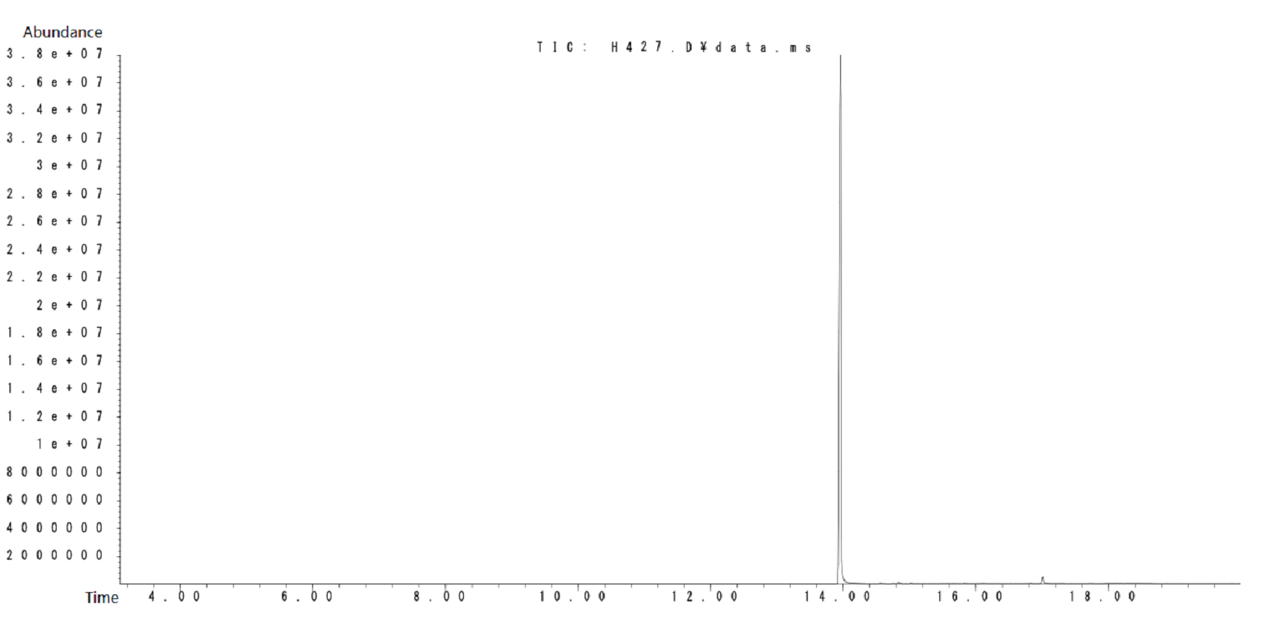
Figure S31:** GC-MS ion flow chromatogram of compound **3a**

**
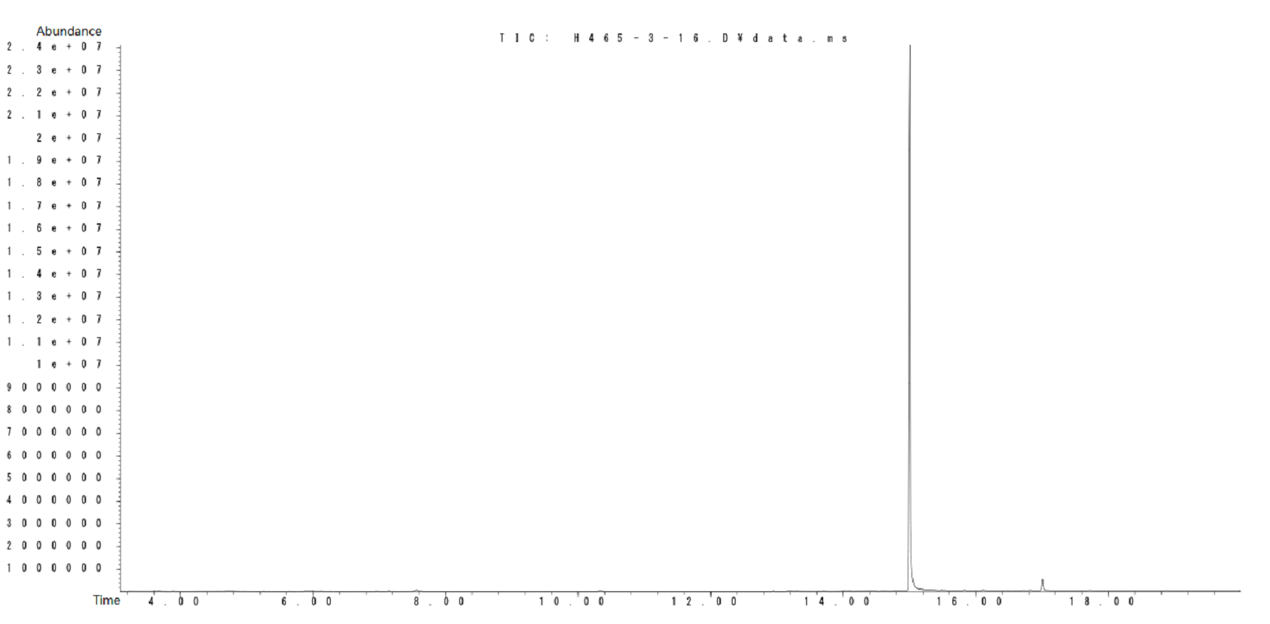
**

**Figure S33:** GC-MS ion flow chromatogram of compound **3b**

**
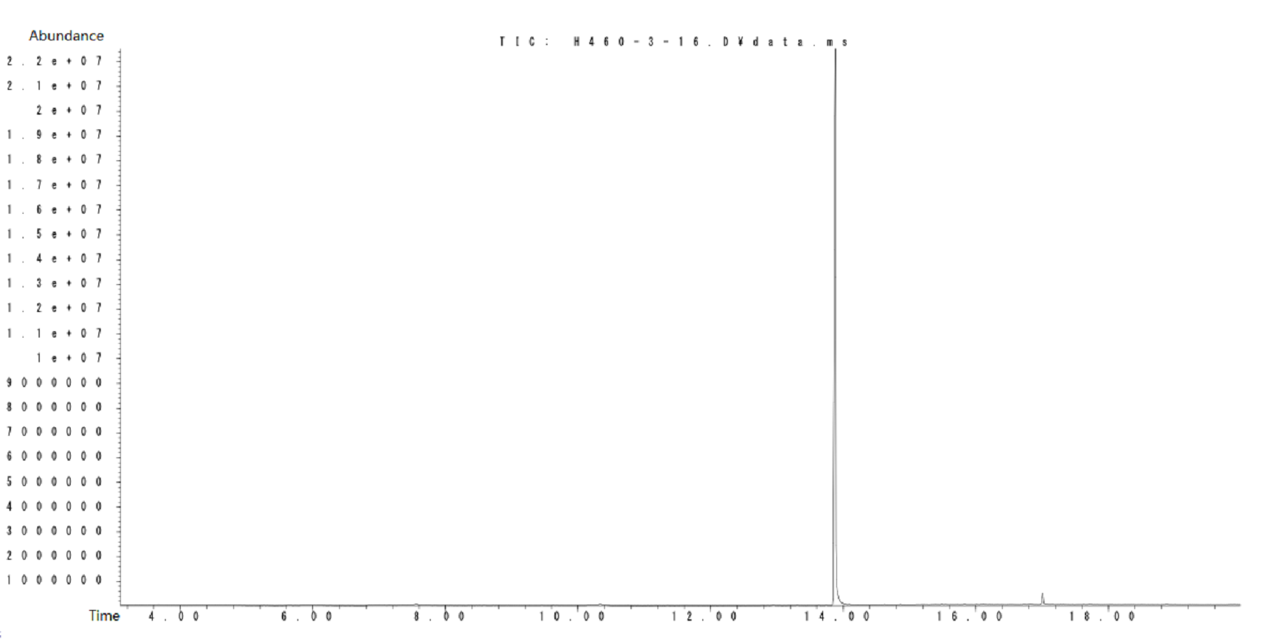
**

**Figure S32:** GC-MS ion flow chromatogram of compound **3c**

**
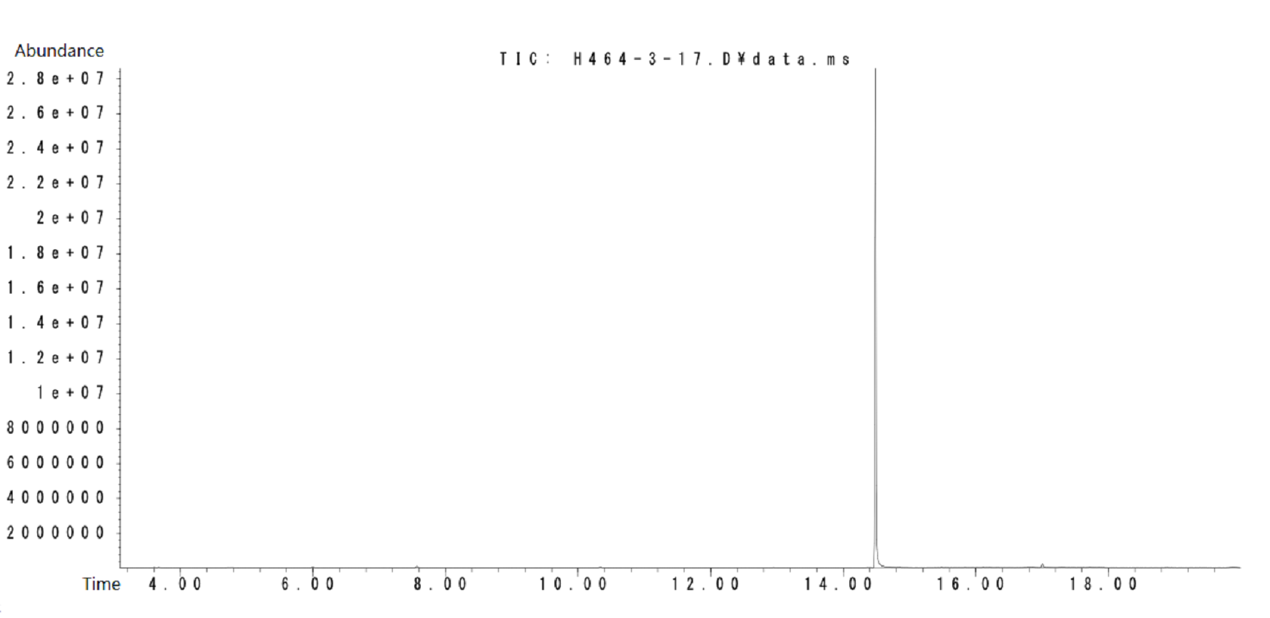
**

**Figure S34:** GC-MS ion flow chromatogram of compound **3e**

**
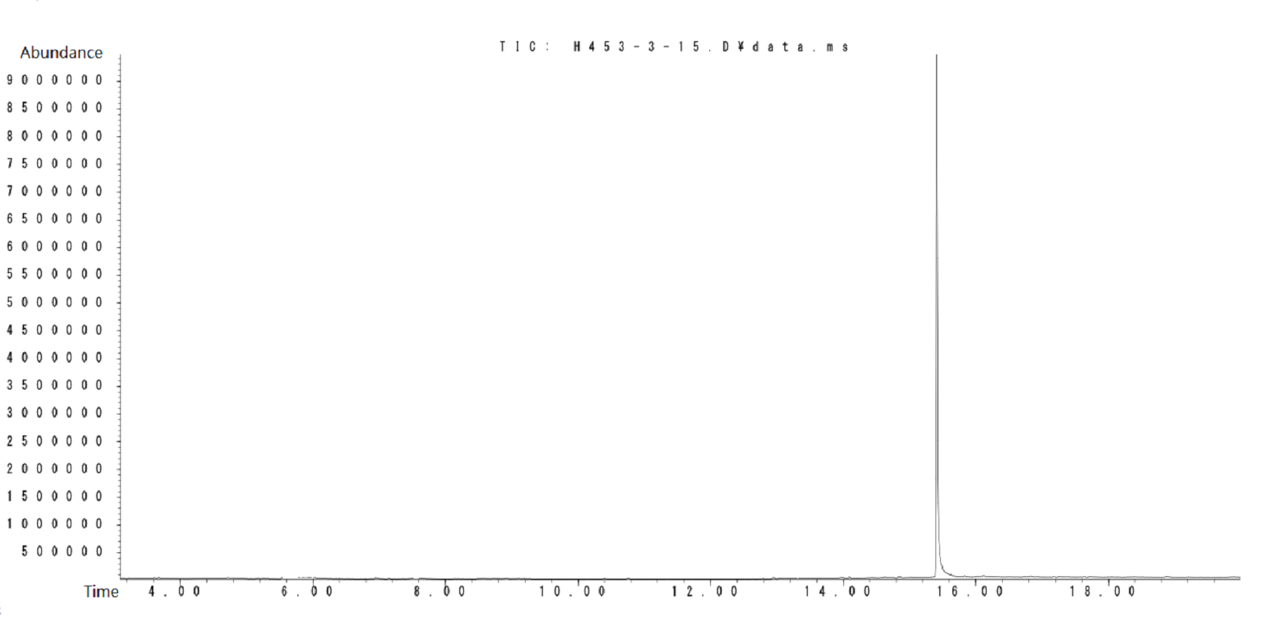
**

**Figure S35:** GC-MS ion flow chromatogram of compound **3f**

**
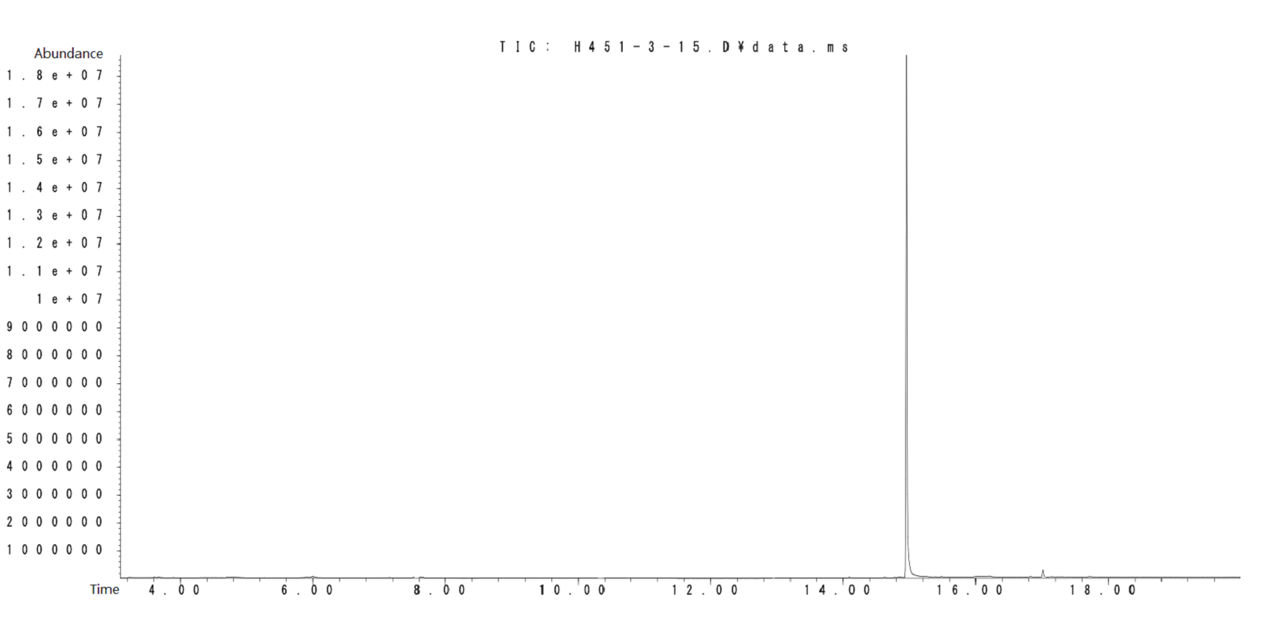
**

**Figure S36:** GC-MS ion flow chromatogram of compound **3g**

**
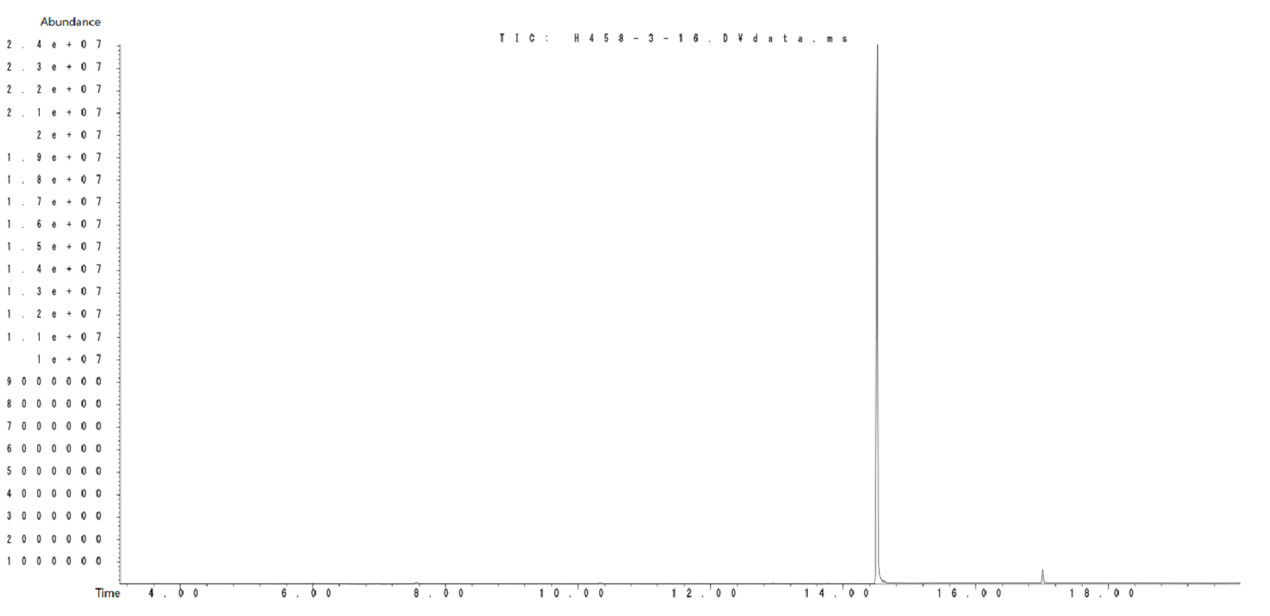
**

**Figure S37:** GC-MS ion flow chromatogram of compound **3h**

**
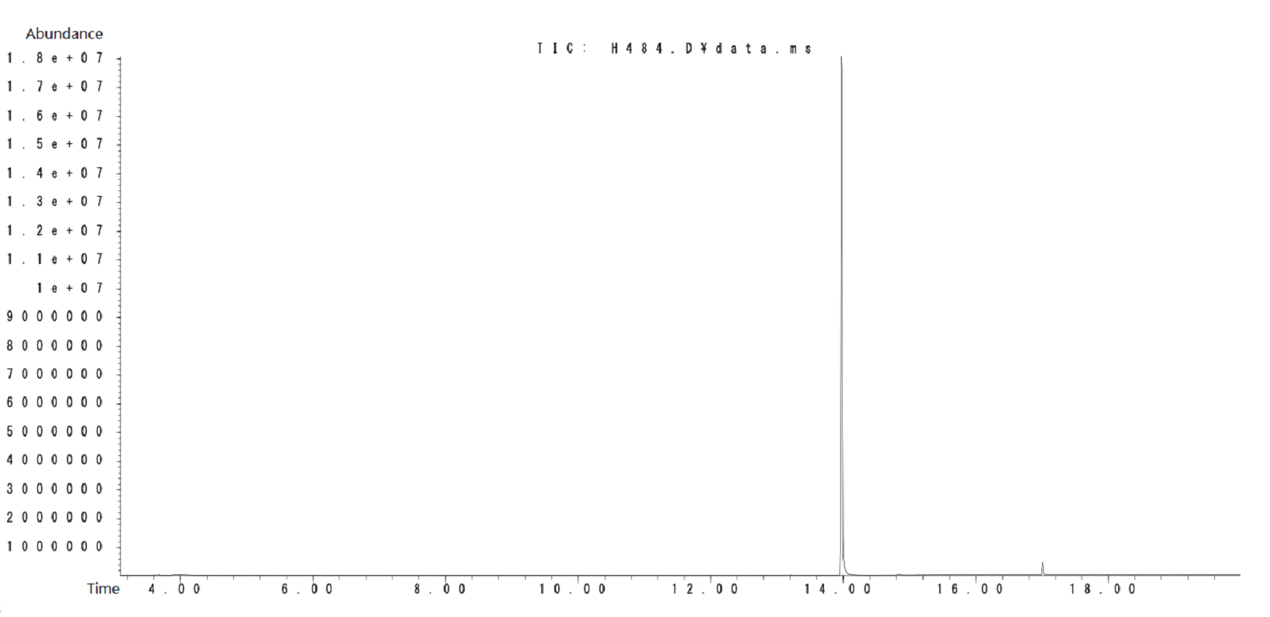
**

**Figure S38:** GC-MS ion flow chromatogram of compound **3i**

**
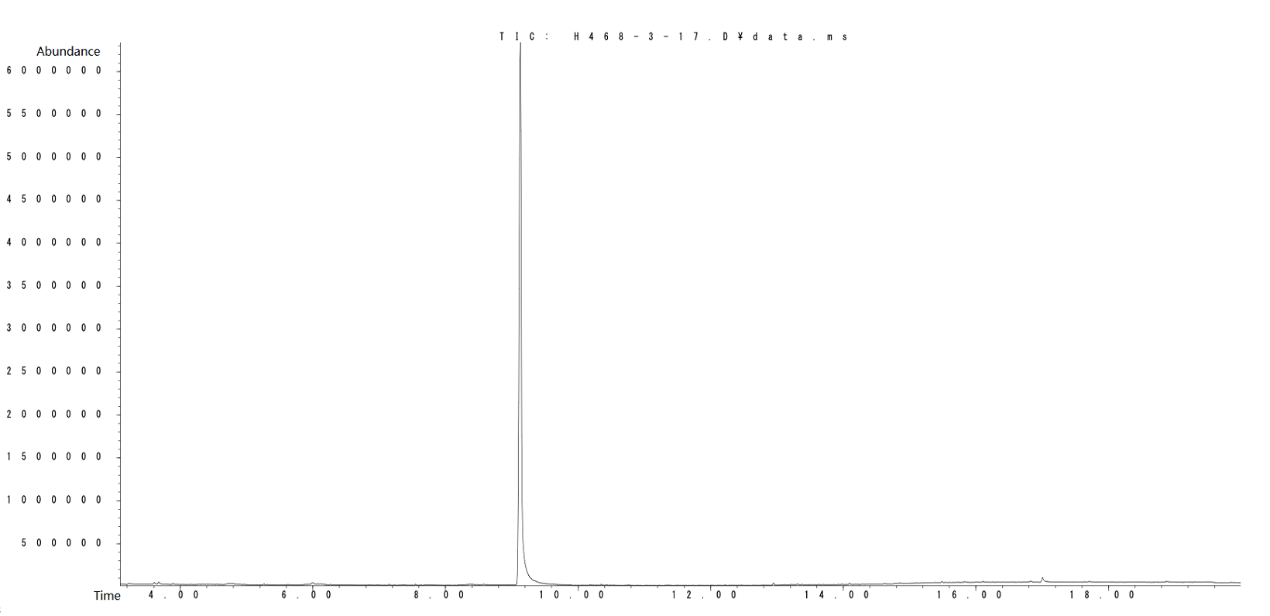
**

**Figure S39:** GC-MS ion flow chromatogram of compound **3j**

**
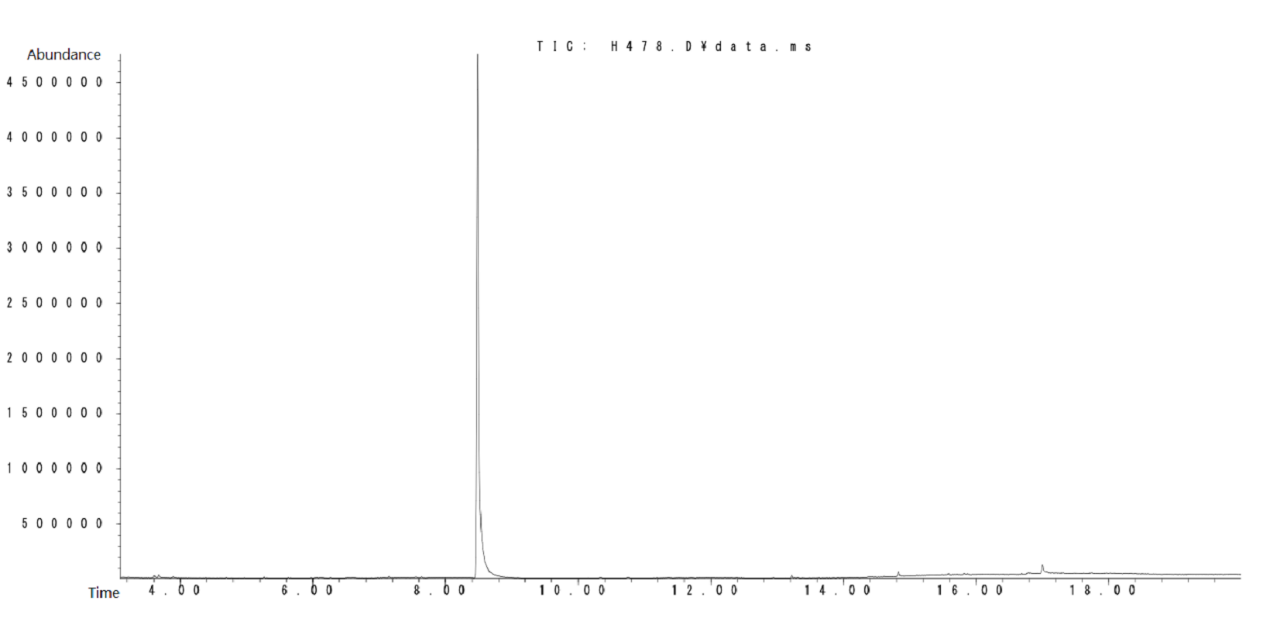
**

**Figure S40:** GC-MS ion flow chromatogram of compound **3k**

**
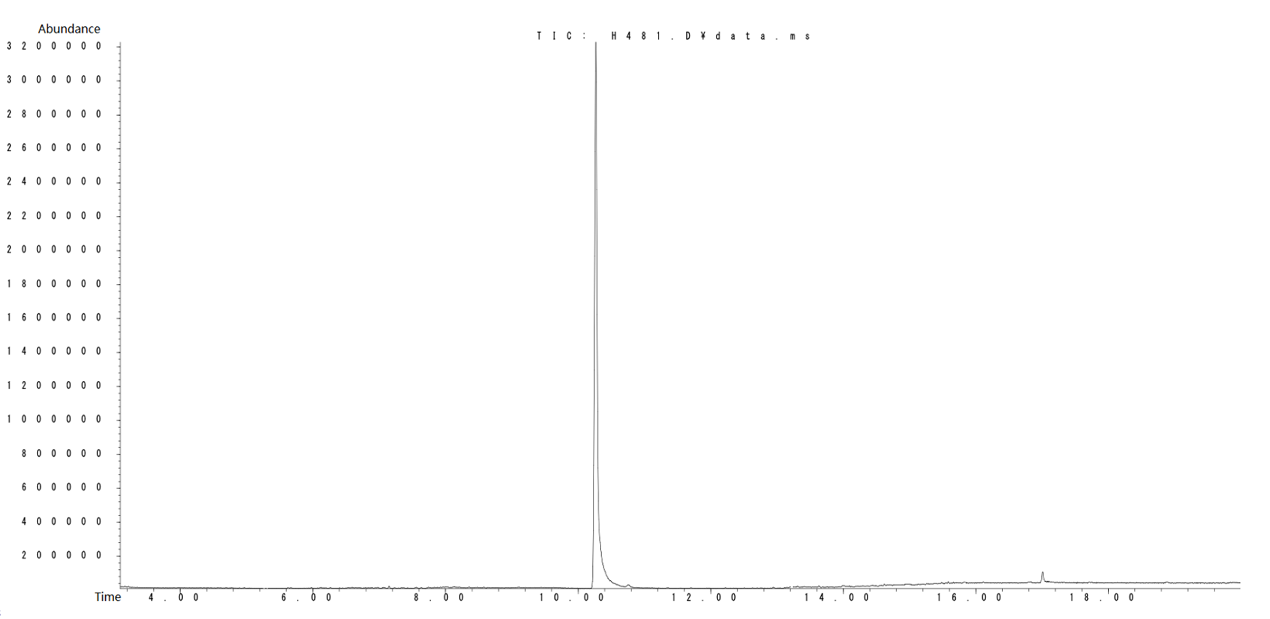
**

**Figure S41:** GC-MS ion flow chromatogram of compound **3l**

**
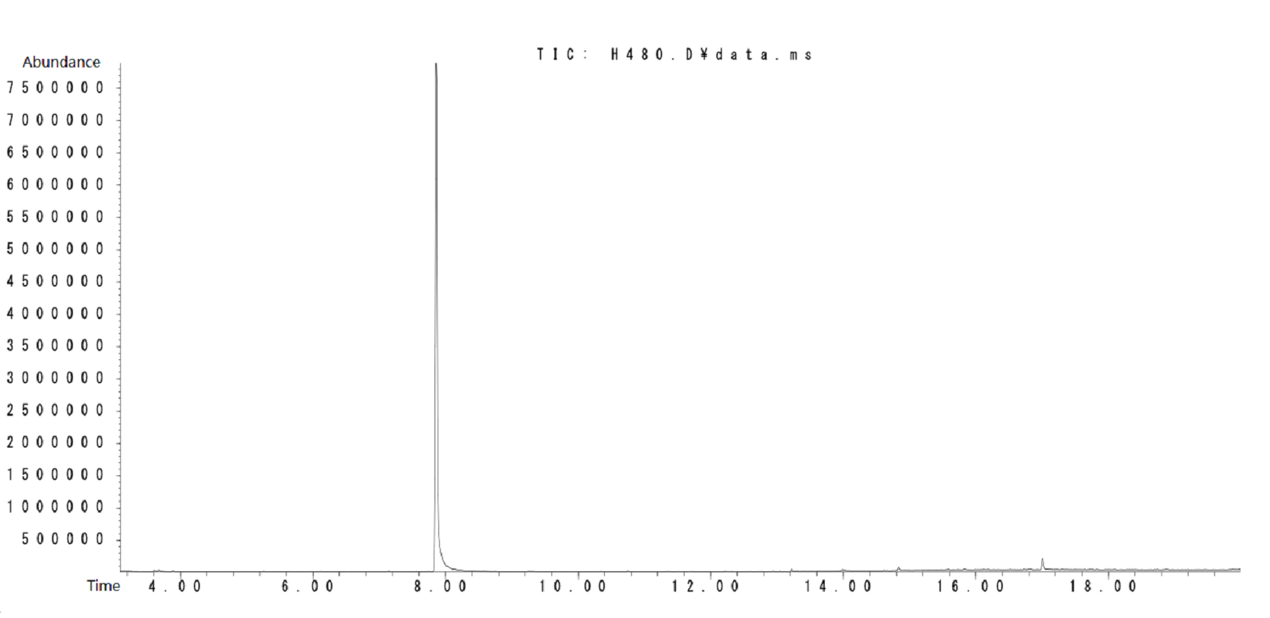
**

**Figure S42:** GC-MS ion flow chromatogram of compound **3m**

**
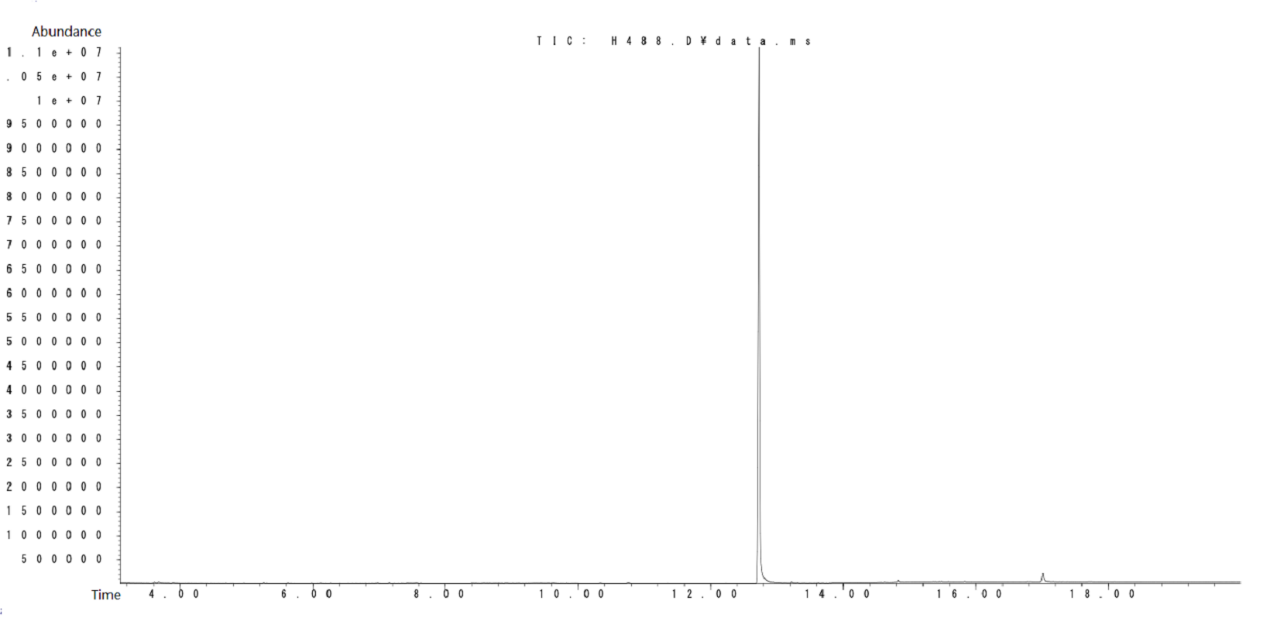
**

**Figure S43:** GC-MS ion flow chromatogram of compound **3n**

**
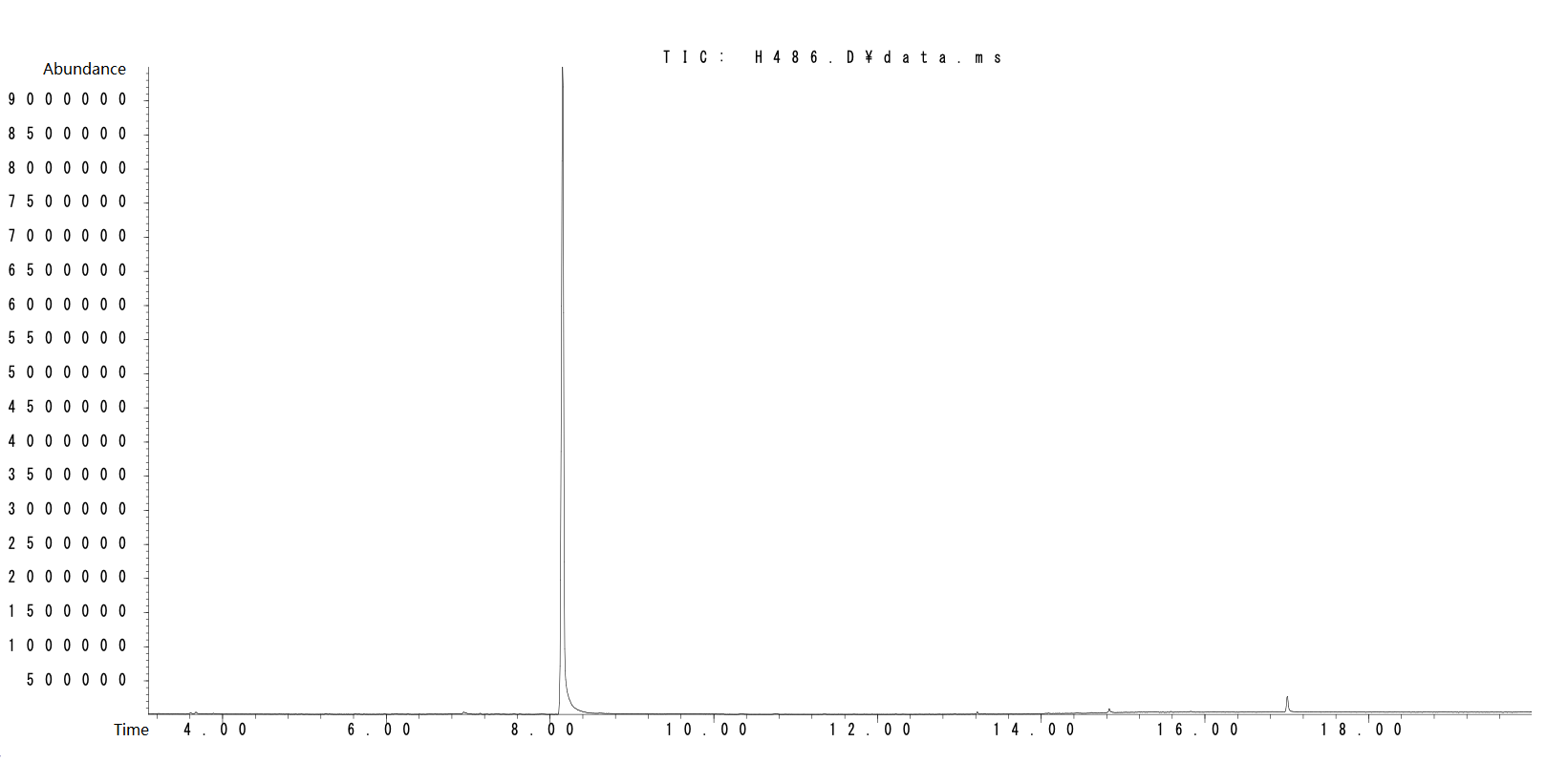
**

**Figure S44:** GC-MS ion flow chromatogram of compound **3o**

**
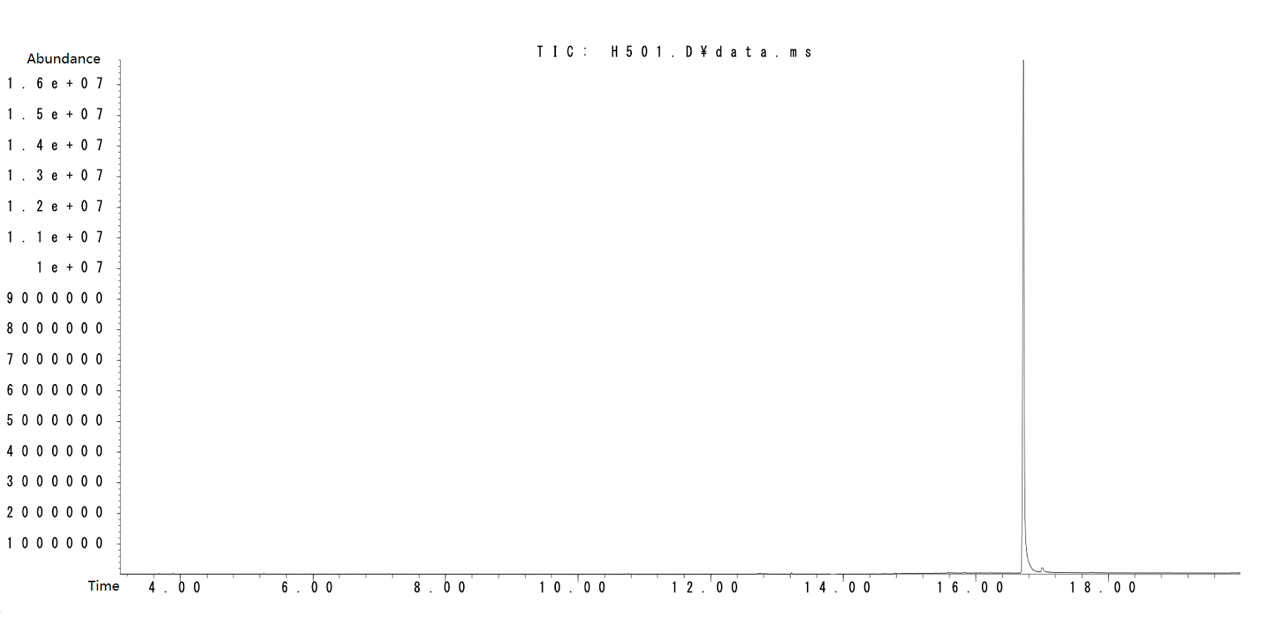
**

**Figure S45:** GC-MS ion flow chromatogram of compound **3p**

1. * Correspondence: [laimiao@henau.edu.cn](mailto:laimiao@henau.edu.cn)

   Flavors and Fragrance Engineering & Technology Research Center of Henan Province, College of Tobacco Science, Henan Agricultural University, Zhengzhou 450002, P. R. China.

   ^2^ Technology Center, China Tobacco Hebei Industrial Co.,Ltd., Shijiazhuang, 050051, P. R. China.

   ^3^ Technology Center, China Tobacco Shanxi Industrial Co., Ltd., Xian 710065, P. R. China. [↑](#footnote-ref-1)
